# Online Multimodal HILIC–ESI-HRMS/ICP-MS/MS Speciation Reveals Remodeling of Sulfur- and Phosphorus-Containing Metabolites Associated with Gold Nanoparticle Exposure in Transgenic (*cp*4 EPSPS Gene) Soybean Callus

**DOI:** 10.1021/acs.jafc.6c09256

**Published:** 2026-10-06

**Authors:** Lilian Seiko Kato, Elisânia Kelly Barbosa Fonseca, Vinnícius Henrique Cerqueira da Silva, Diego Campaci de Andrade, Guilherme Crispim de Faria Cruz, Jorge Henrique Pedrobom, Marco Aurélio Zezzi Arruda

**Affiliations:** † Spectrometry, Sample Preparation and Mechanization Group, Institute of Chemistry and National Institute of Science and Technology in Bioanalytics − Lauro Kubota, Institute of Chemistry, 28132 University of Campinas (Unicamp), Campinas, São Paulo 13083-970, Brazil; ‡ Institutional Mass Spectrometry and Chromatography Laboratory, Institute of Chemistry, 28132 University of Campinas (Unicamp), Campinas, São Paulo 13083-970, Brazil; § Nova Analítica Company, Diadema, São Paulo 09941-202, Brazil

**Keywords:** Top-down metabolite profiling, metal-based nanoparticles (MNPs), plant metallomics, speciomics, nanobiotechnology, redox-active sulfur pathways, Au NPs, *Glycine max*

## Abstract

A multimodal HILIC–ESI-HRMS/ICP-MS/MS workflow profiled sulfur- and phosphorus-containing metabolites in transgenic soybean (*Glycine max*) calli exposed to 100 μg L^–1^ gold nanoparticles (Au NPs). Calli were cultured on proliferation medium for 30 days, with or without Au NPs. Thirty-eight analytically supported P- or S-containing features corresponded to 28 putative annotations. Exploratory analysis showed lower relative signals for reduced sulfur metabolites in Au NP-treated calli: methionine (log_2_FC = −5.20) and l-homomethionine (log_2_FC = −4.10). Oxidized sulfur metabolites (methionine sulfoxide, cysteine sulfinic and sulfonic acids, cystine, and sulfolactate) were detected only in the Au NP-treated profile. At the same time, thiamine showed a higher relative signal (log_2_FC = +2.13) in the treated sample. Phosphorylcholine showed a lower relative signal (log_2_FC = −6.21), whereas LPC(16:0) showed a higher relative signal (log_2_FC = +1.82) in the Au NP-treated profile. These patterns are consistent with differences in sulfur redox, thiamine-associated, nucleotide-sugar, and phospholipid metabolism.

## Graphical abstract

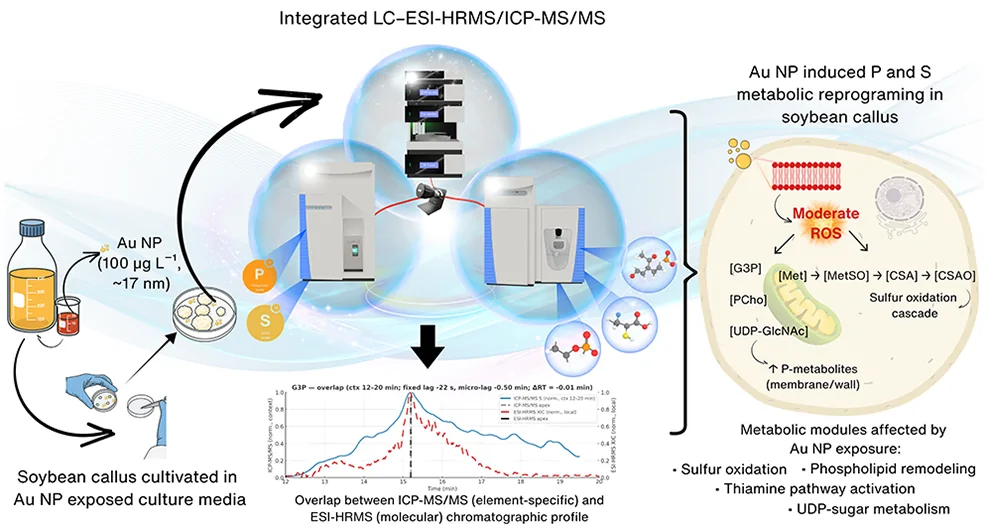

## 1 Introduction

The application of specific metal-based nanoparticles (MNPs) in agriculture can benefit plants,^1^ including increased rates of germination, enhanced growth, greater crop biomass, improved yield, and overall productivity.^2^ Additionally, those NPs can help protect plants against biotic (organism-related) and abiotic (environmental) stresses.^1^ Although the mechanisms by which MNPs exert these positive effects and the metabolic pathways involved are not yet fully understood, the positive outcomes have been demonstrated in numerous studies.^1,3–7^

Gold nanoparticles (Au NPs) can interact with plants following root or foliar exposure, while their uptake and translocation depend strongly on particle size and surface or coating chemistry.^5,8,9^ Reported biological responses also vary substantially with dose, exposure duration, and plant species. In *Arabidopsis thaliana*, exposure to 24 nm Au NPs at 10 and 80 μg mL^–1^ increased total fresh weight by 3.68- and 6.3-fold, respectively, after 7 days, while 10 μg mL^–1^ increased total seed yield threefold.^7^ In soybean, exposure to approximately 19 nm citrate-stabilized Au NPs at 160 mg L^–1^ for 21 days increased fresh biomass by approximately 24% in leaves, 20% in stems, and 33% in roots, and plant height by 13%.^5^ In contrast, foliar application of 0.5 mg L^–1^ Au NPs to lentil leaves produced time-dependent redistribution of Au signals over 96 h but no persistent changes in the spatial distribution of P or other essential nutrients.^9^ At the molecular and cellular levels, Au NP exposure has been associated with altered microRNA expression, antioxidant activity, photosynthetic function, and cell-wall properties.^7,10,11^ However, Au NPs are not universally growth-promoting: non-optimal exposures may induce oxidative stress and toxicity, with responses depending on particle properties, dose, exposure duration, plant species, and culture conditions.^11,12^

In plant biology, the term “callus” (plural “calli”) originally referred to the massive growth of cells associated with wounding, but actually refers more broadly to disorganized cell masses. A callus may arise from a single plant cell that has reverted to an undifferentiated state, and some of these cells retain totipotency, meaning they can give rise to a whole plant.^13^ The advantages of cultivating callus through plant tissue culture include high productivity and micropropagation of endangered or economically valuable plants^12,14^ and the production of virus-free plants.^13^ This approach enables the regeneration of whole plants from tiny tissue fragments, which is why callus culture is commonly used in transformation workflows and toxicology studies.^14^ It also has applications in biotechnology and research, since callus serves as a research tool for studying plants’ cellular behavior and responses to stress, hormonal interactions, and metabolic changes.^12–15^

MNPs can assist in growing plant calluses^14,16^ thereby enhancing the production of antioxidants and secondary metabolites.^12,16^ In *Prunella vulgaris* callus cultures monitored over 49 days, Au NPs produced higher biomass accumulation during the 35–42-day logarithmic growth phase, whereas Ag/Au NP formulations at different Ag:Au ratios, alone or combined with 2.0 mg L^–1^ NAA, increased callus proliferation to 100%, compared with 95% in the control; the 3:1 Ag/Au formulation without NAA produced antioxidant activity of 87.85%.^16^ More recently, transgenic soybean calli exposed to Cu NPs at 10, 50, and 100 μg L^–1^ for 30 days exhibited concentration-dependent chemical remodeling, with the most pronounced alterations occurring at 100 μg L^–1^; the first two principal components explained 77.4% of the total chemical-profile variance.^17^ When exposed to NPs, plant calli create more free radicals and reactive oxygen species (ROS). In response, they produce more antioxidants, which interest the pharmaceutical and nutraceutical industries.^11,12,14^ When applied to plant callus, Au NPs have been reported to influence growth and several cellular features, including ploidy, methylation patterns, and cell wall properties.^10,11,18^ They have also been used as hormone carriers^18^ and can increase metabolite accumulation.^16^ However, these effects depend strongly on particle size, concentration, and loading conditions, and researchers must avoid issues such as mixoploidy, chimerism, or toxicity. Addressing these factors is crucial to developing reproducible tools in plant biotechnology.^11,12,14^

Sulfur (S) is a required macronutrient in soybean plants, and its absence is quickly reflected in their development. S is involved in many metabolic processes, such as the synthesis of amino acids and vitamins, and in the amount and composition of seed proteins.^20,21^ Phosphorus (P) is another essential macronutrient for plants, participating directly in root formation, seed production, and nitrogen fixation.^22^ P deficiency severely limits global crop productivity by altering plant morphology, delaying reproductive development, and restricting photosynthetic capacity through stomatal constraints and biochemical limitations such as reduced Rubisco activity.^23^ In this scenario, soybeans tend to adjust their membrane composition, where phospholipids may be replaced by sulfolipids or galactolipids, for example, a strategy that helps preserve membrane structure.^22^

The use of MNPs may affect P and S dynamics in plants, with effects varying by NP type, dosage, application method, and plant species.^5,9,12^ Recent studies illustrate this context dependence: Au NP exposure altered nutrient homeostasis in soybean, whereas low-dose foliar exposure produced no persistent changes in the spatial distribution of P or other nutrients in lentil leaves.^5,9^

Successfully identifying S- and P-containing metabolites in plants can be challenging.^23,24^ Raab, Ronzan, and Feldmann (2017) conducted a pivotal global S speciation study, uncovering up to 54 sulfur compounds in *Allium sativum*. They used a multimodal approach combining high-performance liquid chromatography (HPLC), inductively coupled plasma tandem mass spectrometry (ICP-MS/MS), and electrospray ionization high-resolution mass spectrometry (ESI-HRMS).^24^ This combined approach offers advantages for identifying P- and S-containing metabolites in plants, enhancing sensitivity and enabling element-specific detection. Thus, combining ICP-MS (for element specificity) and ESI-HRMS (for structural specificity) significantly increases confidence in determining both known and unknown compounds, thereby reducing reliance on authentic standards for putative annotation, although standards remain necessary for definitive identification and absolute quantification.^17,25,26^

ICP-MS/MS is effective in reducing isobaric interferences that complicate the detection of P and S, since P is measured as ^31^P^16^O^+^ (m/z 47), and S as ^32^S^16^O^+^ (m/z 48). This coupling can also detect compounds that may escape ESI analysis or be missed in conventional HRMS workflows.^17,25^ The complete online coupling (HILIC–ESI-HRMS/ICP-MS/MS) prevents degradation and ensures analyte integrity, compared, for example, with the offline setup.^25,26^ Kato et al. (2024) recently demonstrated this approach to identify P-, As-, and S-based biomolecules in sea turtle liver samples.^25^ More recently, the same integrated strategy was applied to profile element-containing chemical species in transgenic soybean callus exposed to copper nanoparticles.^17^ Thus, this type of multimodal setup enables the collection of elemental and molecular information in a single workflow. This integration benefits metallomics and speciomics studies.^25,27^

Despite the recognized roles of P- and S-containing metabolites in plant biochemical regulation, detailed molecular and element-specific information on how nanoparticle exposure alters these metabolites remains limited. Conventional LC–HRMS provides molecular and structural information but lacks direct elemental specificity, whereas standalone ICP-MS/MS detects elemental signatures without resolving the corresponding molecular structures.^25^ Although a recent online multimodal speciation study characterized element-containing chemical species in transgenic soybean callus exposed to Cu NPs, comparable information on P- and S-containing metabolites in Au NP-exposed soybean callus remains unavailable.^17^

Taken together, the studies, including recent experimental investigations of Au NP effects on in vitro proliferation, somatic embryogenesis, ionic regulation, and nutrient homeostasis, demonstrate that research on Au NPs in plant biotechnology has continued beyond the earlier literature.^5,9,10,28,29^ Recent reviews further emphasize that the molecular mechanisms underlying these context-dependent responses remain incompletely understood, particularly in plant tissue-culture systems.^11,30^ Accordingly, Au NPs were selected in the present study as a well-characterized model nanomaterial for probing P- and S-associated biochemical responses under controlled callus conditions rather than as a proposed agricultural input. We used an online multimodal HILIC–ESI-HRMS/ICP-MS/MS workflow to correlate molecular ions with their corresponding P and S elemental signatures in transgenic soybean callus exposed to 100 μg L^–1^ Au NPs for 30 days, and compared them with untreated controls. We used this workflow for qualitative and relative metabolite profiling rather than absolute quantification. We evaluated whether Au NP exposure was associated with differences in the relative profiles of P- and S-containing metabolites. To our knowledge, this study provides the first element-resolved molecular characterization of P- and S-containing metabolic profiles in Au NP-exposed soybean callus. By linking molecular ions with element-specific P and S signals, this study demonstrates the value of multimodal speciomics for resolving element-associated metabolic patterns. It provides an analytical basis for future mechanistic studies of plant–nanoparticle interactions.

## 2 Materials and Methods

### 2.1 Chemical Reagents

All solutions, dilutions, and standards were prepared using high-purity deionized water (18.2 MΩ·cm) from a Milli-Q system (Millipore, Bedford, USA) and analytical-grade reagents. Standard solutions used for experiments were prepared from appropriate arsenobetaine (AsB) C_5_H_11_AsO_2_ (Sigma-Aldrich) dilutions. All HPLC mobile phases were prepared from high-purity solid ammonium formate (99.995% purity) (Sigma-Aldrich, Germany) dissolved in ultrapure water. HPLC-grade methanol and acetonitrile (Merck, Germany) were used for the mobile phase. High-purity argon 4.8 was used as the ICP-MS/MS nebulizer, auxiliary gas, and plasma gas (White Martins, São Paulo, Brazil). High-purity oxygen 6.0 (White Martins, São Paulo, Brazil) was used as an additional collision cell gas. High-purity nitrogen 5.0 was used as the nebulizer and desolvation gas in the ESI source, and as the collision gas in the high-energy collision dissociation cell (HCD).

### 2.2 Soybean Callus Cultivation

Soybean seeds of the transgenic cultivar RR (Roundup Ready) were placed in 50 g of a mixture containing an equal percentage of a commercial pine-bark-based horticultural substrate (BasaPlant, Base Substratos, Holambra, São Paulo, Brazil) and expanded vermiculite (Vermfloc Agro, Brazil) and grown in a BOD (Biochemical Oxygen Demand) germination chamber with a 12-hour light/12-hour dark photoperiod and 26 °C temperature for 21 days. The plants were watered daily with 10 mL of Milli-Q water. After this period, soybean plants with expanded stems, leaves, and cotyledons were excised. The explants were sterilized by immersing the tissues in three ethanol solutions (70%), with a 1 min dwell time in each solution, followed by an 8 min dwell time in a sodium hypochlorite solution (2%), and then washed with running water. After that, all the tissues were cut into 0.3–0.5 cm long pieces and placed in the callus induction medium, Murashige and Skoog (MS basal salts, 3% sucrose, 150 mg/L casein hydrolyzate, and 0.8% agar supplemented with 2,4-dichlorophenoxyacetic acid, pH 5.7), and maintained at 25 °C under white light and a 16-hour day/8-hour night cycle. After 4 weeks, callus was excised from the tissue pieces, plated on callus proliferation medium (MS basal salts, 10 mL of vitamins −0.1 g thiamine and 10 g Myo-inositol in 1 L solution, 3% sucrose, 0.8% agar supplemented with 2,4-dichlorophenoxyacetic acid, pH 5.6) and kept in an incubator at 25 °C in the dark, being subcultured every 3 weeks to a fresh medium^31^ (Figure S1 in the Supporting Information). This study reports only callus generated from soybean cotyledons.

### 2.3 Anatomical Analyses of Callus through Light and Fluorescence Microscopy

For anatomical analyses conducted before Au NP exposure, the method described by Ferrari et al. (2021) was applied.^32^ The proliferated soybean callus obtained from the cotyledon was fixed in FAA 50 solution (5:5:90 formaldehyde:acetic acid:50% ethanol). A vacuum pump was then used to remove the air from the tissues. The samples were dried in an ethanol series and then embedded in plastic resin (Leica Historesin, Leica Microsystems, Wetzlar, Germany) according to the manufacturer’s instructions. The samples were sectioned using a Leica RM2245 manual rotary microtome. Sections (5 μm thick) were stained with toluidine blue 0.05% in phosphate and citrate buffer, pH 4.5, and mounted in Entellan (Merck KGaA, Darmstadt, Germany) synthetic resin. The imaging was performed using an Olympus DP71 video camera attached to an Olympus BX51 microscope.

### 2.4 Addition of Au NPs in the Soybean Calluses and Sample Preparation for Analysis

Au NPs were synthesized and characterized as described previously by Fonseca et al. (2026)^5^ with a hydrodynamic diameter of 17.08 nm, a mean TEM size of 16.8 ± 2.1 nm (n = 3), a zeta potential of −37.18 mV, and a spherical shape. Each experimental condition comprised ten Petri dishes containing approximately seven calli per dish. For the treated condition, Au NPs were added to the callus proliferation medium at a concentration of 100 μg L^–1^. Treated and control calli were maintained in the dark for 30 days under otherwise identical conditions. After incubation, we combined all calli from the ten dishes in each condition and ground them under liquid nitrogen. We extracted 200 mg from each homogenized pool with 1.5 mL of extraction solution. Additional sample-preparation details are provided in Section 1.2 of the Supporting Information.

### 2.5 Total Elemental Determination of P, S, and Au

Total P, S, and Au concentrations were determined using the microwave-assisted decomposition procedure previously described by Fonseca et al.^33^ Three independent samples from each experimental group were analyzed (n = 3). Results are expressed as mean ± standard deviation. Moisture content was determined separately, and the elemental concentrations were corrected and expressed as mg kg^–1^ on a dry-weight basis. The LOD and LOQ established for the measured solutions were 0.029 and 0.096 mg L^–1^ for P, 0.301 and 1.002 mg L^–1^ for S, and 0.04 and 0.13 μg L^–1^ for Au, respectively. Differences between untreated and Au NP-treated calli were evaluated using a two-sided Welch’s t-test, with p < 0.05 considered statistically significant.

### 2.6 Online Multimodal LC–ESI-HRMS/ICP-MS/MS Coupling

The LC-ESI-HRMS/ICP-MS/MS platform was configured as an online multimodal workflow, in which the HILIC effluent was split between the Orbitrap ESI-HRMS and the ICP-MS/MS detector. The multimodal coupling was optimized, configured, and performed according to previous work by Kato et al. (2024).^25^

#### 2.6.1 Detailed ICP-MS/MS Acquisition Modes and Instrument Parameters for Coupling

Table 1 presents the instrumental configurations employed in the iCAP-TQ Thermo ICP-MS/MS.

**Table 1:** ICP-MS/MS Instrument Parameters for the Organic Interface

Tune parameter	Chosen value
Torch position and CR-cell gas flow	Tuning in a standard mode
Cyclonic chamber temperature	–5 °C
Peristaltic pump rotations during tuning and performance	40 rpm
Plasma power	1550 W
Plasma gas	14 L min^–1^
Coolant gas	0.8 L min^–1^
Nebulizer gas	0.56 L min^–1^
Additional gas level (O_2_)	20%

#### 2.6.2 HPLC Conditions

An Accucore Amide HILIC column (250 × 2.1 mm, 2.6 μm) was used for separation. The mobile phases were acetonitrile (A) and 5 mM ammonium formate (pH 5.5) (B). The run was performed at 0.750 mL/min with the following gradient: 10% B (0–5 min), ramping to 50% B until 45 min, held to 50 min, increased to 65% B to 55 min, and then returned to 10% B for re-equilibration (55–60 min). The injection volume was 20 μL, and the column temperature was 35 °C.^25,34^

#### 2.6.3 Optimization of Flow Splitting and PEEK Lengths

A flux splitter (Series 600 valve, ASI Company, USA) simultaneously connected the LC with the ICP-MS/MS and high-resolution mass spectrometer with electrospray ionization Thermo Orbitrap QExactive (ESI-HRMS) detectors. Detailed optimization and parameters are described in Supporting Information, Section 1.6.

#### 2.6.4 Detailed ESI-HRMS Acquisition Modes and Instrument Parameters for Coupling

Tables S1 and S2 in the Supporting Information (Section 1.7) show the instrumental configurations of the ESI source and the FullMS-ddMS^2^ experiment (untargeted analysis).

### 2.7 Data Processing, Analysis, Quality Control and Metabolite Annotation

All MS^1^, MS^2^, and ICP-MS/MS data were processed using a custom Python workflow that integrated precursor detection, isotopic checks, MS^2^ confirmation, and HRMS × ICP-MS/MS co-elution analysis. The analytical workflow was designed for qualitative and relative metabolite profiling rather than absolute metabolite quantification. Normalized peak areas were used for descriptive comparisons of relative signal intensities between the single pooled untreated and Au NP-treated samples. For features detected in both conditions, log_2_FC was calculated as log_2_(Au NP-treated/control) from the normalized peak areas and was used descriptively to indicate the direction and magnitude of relative signal differences. Because compound-specific calibration curves and authentic standards were not employed, absolute metabolite concentrations were not determined. Arsenobetaine (AsB), added to the samples at 50 μg L^–1^ as an internal standard, was used to correct retention-time lag and as a quality-control marker to evaluate MS^1^ detection, MS^2^ confirmation, and chromatographic overlap with the ^75^As^16^O^+^ signal obtained by ICP-MS/MS. AsB met the established quality-control criteria in both the control and Au NP-treated datasets, confirming the performance of Scripts 1–3. Feature retention and annotation support were evaluated using the established mass-accuracy, fragmentation, and element-specific overlap procedures detailed in the Supporting Information (Section 1.5.2 and Table S7). Complete methodological and quality-control results are provided in Sections 1.5 and 2.2 of the Supporting Information. The complete processing pipeline (Scripts 1–4) is publicly available in Zenodo.^35^

### 2.8 Ethical Statement

This research did not involve human or animal participants.

## 3 Results and Discussion

### 3.1 Anatomical Analysis of Soybean Callus

Gross visual inspection after the 30-day incubation revealed no visible deterioration in the Au NP-treated calli, whereas the untreated control showed greater visible browning, consistent with an oxidized appearance (Figure S2). Figure S4 in the Supporting Information (Section 2.1) presents the baseline anatomical characterization of cotyledon-derived soybean callus before Au NP exposure. Histological sections revealed a heterogeneous and disorganized arrangement of loosely associated parenchymatous cells. The cells varied in size, cytoplasmic density, and degree of vacuolation. Darkly stained nuclei, sometimes accompanied by conspicuous nucleoli, were visible in several cells, whereas other cells were larger and highly vacuolated. Irregularly distributed intercellular spaces and limited cell-to-cell organization were also observed. Together, these characteristics are consistent with the heterogeneous morphology commonly reported for friable callus produced in vitro.^36^ Because microscopy was performed only before treatment, these images were used solely to characterize the starting biological material and were not used to assess Au NP-induced anatomical changes or post-exposure tissue injury.

### 3.2 Total P, S, and Au Concentrations in Soybean Callus

Total elemental analysis was performed to provide complementary quantitative context for the relative profiles of P- and S-containing metabolites. The untreated calli contained 4087.35 ± 14.91 mg kg^–1^ P and 5030.71 ± 417.60 mg kg^–1^ S on a dry-weight basis. In calli exposed to 100 μg L^–1^ Au NPs, the corresponding concentrations were 4651.88 ± 366.89 mg kg^–1^ P and 5091.93 ± 288.76 mg kg^–1^ S. Although the mean P concentration was numerically 13.8% higher in the treated group, this difference was not statistically significant (Welch’s t-test, p = 0.116). Mean S concentration differed by only 1.2% between groups and was also not statistically significant (p = 0.846). Total Au remained below the analytical LOQ in both untreated and Au NP-treated calli. Thus, exposure to 100 μg L^–1^ Au NPs did not produce a statistically demonstrable change in the bulk P or S concentrations and did not result in quantifiable total Au accumulation under the present analytical conditions.

The absence of quantifiable Au should not, however, be interpreted as evidence of complete absence of Au from the treated callus. The solution-based Au LOQ of 0.13 μg L^–1^ establishes only that the Au signal in the digested samples was below the validated quantification threshold. It cannot be used to calculate an uptake percentage or an upper tissue concentration without considering the dry sample mass, final digest volume, and dilution factor. In whole soybean plants exposed to 160 mg L^–1^ citrate-stabilized Au NPs, Fonseca et al. (2026)^5^ detected total Au only in roots (1.66 ± 0.27 mg kg^–1^ dry mass), while leaves and stems remained below the LOQ; the treatment nevertheless selectively altered nutrient homeostasis, whereas total S was not significantly affected. That exposure concentration was 1600-fold higher than the 100 μg L^–1^ used here, and that study did not determine P. Consequently, the studies cannot be compared quantitatively, but together they indicate that soybean responses to Au NP exposure may occur with restricted or analytically undetectable total Au accumulation. Da Silva and Arruda (2023)^37^ reported that soybean callus exposed to 200 μg L^–1^ citrate-stabilized Ag NPs accumulated only 4.1 ± 0.1 ng g^–1^ Ag, corresponding to 0.06 ± 0.01% of the tracked amount, and that almost all Ag detected in the callus was present in ionic form. The authors associated this limited uptake with nanoparticle agglomeration in the MS medium. Although Ag and Au NPs differ in their physicochemical behavior, this closely related callus model supports the possibility that aggregation in the culture medium limited nanoparticle bioavailability in the present study. However, because the residual medium was not analyzed, we cannot directly confirm Au retention in the medium. Future studies should therefore include multielemental analysis of both callus tissue and the residual culture medium to assess broader nutrient homeostasis and determine the fate of Au within the culture system.

The absence of significant differences in total P and S does not preclude exposure-associated differences in the relative signals of individual P- and S-containing metabolites. Total elemental determination integrates inorganic, protein-bound, lipid-associated, nucleotide-associated, and low-molecular-weight forms of each element and therefore does not resolve changes in their molecular distribution. Sulfur-speciation studies have similarly demonstrated that bulk elemental and species-resolved analyses provide complementary biochemical information.^24^ In the present study, the lower normalized signals of methionine and l-homomethionine, together with the putative detection of oxidized S-containing metabolites in the Au NP-treated callus, are consistent with a shift in the relative representation of reduced and oxidized S-containing forms rather than depletion of the total cellular S pool. Nevertheless, because these metabolite differences were based on relative signal comparisons, this interpretation should be regarded as hypothesis-generating. Since the present study focused specifically on P- and S-containing metabolites, the total elemental analysis was restricted to P, S, and Au and does not constitute a broader assessment of micronutrient homeostasis.

### 3.3 Multimodal Annotation of P- and S-Containing Features

Tables S5–S7 in the Supporting Information (Section 2.3) summarize the successive stages of the multimodal workflow: detection of candidate P- and/or S-containing molecular features from MS^1^ data using Script 1, evaluation of structural support from MS^2^ spectra using Script 2, and assessment of chromatographic overlap with P or S elemental traces obtained by ICP–MS/MS using Script 3, respectively. Features detected in the analytical blank or failing the established criteria for MS^2^ support or elemental co-elution were excluded from the final dataset and are reported in Table S8 (Supporting Information, Section 2.3).

Figure 1 illustrates the complementary analytical evidence supporting the putative annotation of LPC(16:0) in untreated soybean callus extracts. The positive-ion MS^2^ spectrum of the protonated precursor at m/z 496.3397 shows the diagnostic phosphocholine product ion at m/z 184.0738 and the choline-related ion at m/z 104.1073, with mass errors below 5 ppm. The product ion at m/z 478.3308 corresponds to the neutral loss of 18.011 Da (H_2_O) from the precursor.

**Figure 1:**
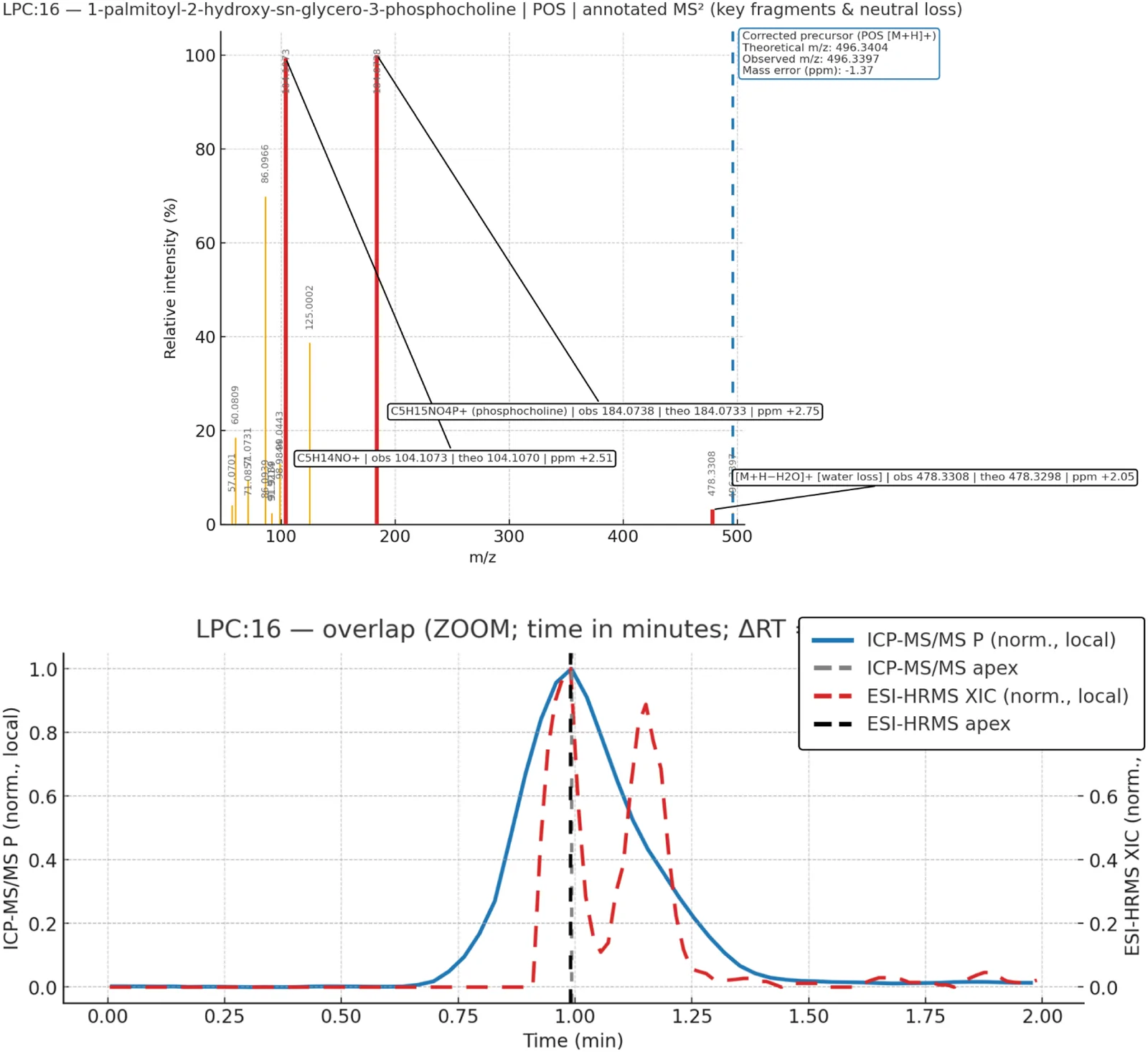
Multimodal analytical support for the putative annotation of LPC(16:0) by MS^2^ and LC–HRMS/ICP-MS/MS co-elution. (A) The annotated MS^2^ spectrum of LPC(16:0) putatively annotated in the untreated soybean callus sample is presented in positive ionization mode. The blue dashed line indicates the corrected precursor ion, while the red vertical lines highlight the characteristic product ions, including key fragments and neutral losses, used to support the structural annotation. All of these fall within an acceptable mass error tolerance (<5 ppm). (B) Chromatographic overlap between ESI-HRMS (XIC) and the ICP-MS/MS P signal, exhibiting a co-eluting peak at the same retention time. This co-elution supports the association of the HRMS ion with the P-containing chromatographic feature, although co-elution alone does not establish definitive molecular identity.

After correction for the 22 s interdetector lag established for the untreated sample, an LPC(16:0) XIC peak apex aligned with the phosphorus signal monitored by ICP–MS/MS at approximately 1.0 min. This co-elution provided element-specific support for associating the HRMS feature with a P-containing chromatographic species but did not, by itself, establish definitive molecular identity. The remaining retained features were evaluated using the same complementary MS^2^ and elemental-overlap workflow. Complete annotated MS^2^ spectra and HRMS–ICP-MS/MS overlap plots for each retained putative annotation are provided in the Supporting Information (Section 2.3, Figures S7–S43).

As described above, retention of a putative metabolite annotation for comparative analysis required three complementary lines of evidence: (i) accurate mass and plausible adduct formation in MS^1^, (ii) structurally diagnostic information from MS^2^, and (iii) chromatographic co-elution with P or S elemental traces monitored by ICP–MS/MS. Together, these criteria increased confidence in the molecular assignments but, without authentic standards, did not constitute definitive compound identification. Integration of the HRMS and ICP–MS/MS data was not always straightforward because P- and S-containing features exhibited complex chromatographic behavior under HILIC conditions, including subtle differences in retention profiles and peak shapes between the two detection systems.^38^ These effects occasionally produced temporal misalignment or reduced correlation between chromatographic traces, even when the proposed annotation was supported by MS^2^ fragmentation patterns. Accordingly, the retained features are reported as high-confidence putative metabolite annotations, and their normalized peak areas were used solely for relative comparisons between treatment groups rather than for absolute quantification.

Limited signal intensity and low structural information content in some fragmentation spectra, especially for very small or structurally simple molecules, prevented unambiguous confirmation of some initial annotations.^38^ In other cases, informative MS^2^ spectra were accompanied by weak or inconsistent elemental signals, reflecting differences in the operating principles, sensitivities, and signal responses of the two detection systems.^26^ Candidate features that did not satisfy the combined analytical criteria were excluded. The final dataset comprised 38 analytically supported ion/adduct features corresponding to 28 unique high-confidence putative metabolite annotations, which were carried forward for relative comparison and biological interpretation.

### 3.4 Shared P- and S-Containing Putative Metabolite Annotations and Relative Abundance Patterns

Across both experimental groups, the integrated LC–ESI-HRMS/ICP-MS/MS workflow retained 38 analytically supported ion/adduct features corresponding to 28 unique high-confidence putative metabolite annotations. The untreated callus yielded 15 ion/adduct features, seven S-containing and eight P-containing, corresponding to 14 unique annotations. The Au NP-treated callus yielded 23 features, 12 S-containing, 10 P-containing, and one containing both elements, corresponding to 22 unique annotations. The groups shared eight unique annotations. Figure S45 in the Supporting Information (Section 3) presents the relative signal intensities of the retained features, expressed as log_10_-transformed normalized LC–HRMS peak areas, in untreated calli and calli exposed to 100 μg L^–1^ Au NPs.

According to Figure S44, the analysis of metabolites detected in both control and Au NPs-treated soybean callus samples revealed a conserved core of S- and P-containing metabolites that are involved in essential cellular pathways. Common S-containing compounds in both groups include Cys-pro, l-homomethionine, methionine, and thiamine (vitamin B1). These molecules play fundamental roles in S metabolism, including amino acid biosynthesis, redox balance, methylation reactions, and regulation of cofactor-dependent energy metabolism.^39^ Cys-pro and methionine derivatives are precursors for thiol-containing antioxidants and serve as methyl donors.^40,41^ At the same time, thiamine is essential for carbohydrate metabolism and is known to accumulate during oxidative stress or metal exposure.^42^ l-Homomethionine can be produced through side-chain elongation or transamination reactions linked to the turnover of methionine and S-adenosylmethionine (SAM), processes that are active in proliferating tissues.^43^ Moreover, l-homomethionine has been identified as a minor intermediate in the conversion between methionine and S-methylmethionine (SMM). This metabolic cycle is crucial for S storage, methylation, and stress responses. This way, the presence of l-homomethionine in both samples highlights the dynamic nature of methionine metabolism in callus tissues.^43,44^

P-containing metabolites shared between the two groups include phosphorylcholine, uridine monophosphate (UMP), 1-palmitoyl-2-hydroxy-*sn*-glycero-3-phosphocholine (LPC:16), and glycerol-3-phosphate (G3P). These metabolites participate in key biochemical pathways, including nucleotide turnover, phospholipid metabolism, and energy regulation.^45–47^ Phosphorylcholine participates directly in the Kennedy pathway and contributes to the formation and maintenance of phosphatidylcholine, one of the major membrane lipids in plants.^48,49^ LPC:16 is a lysophospholipid formed during membrane turnover through phospholipase activity. Its presence is often linked to lipid recycling processes and signaling events that occur when cells experience metabolic or environmental stress.^50,51^ G3P lies at the intersection of glycolysis and glycerophospholipid biosynthesis, and also contributes to maintaining metabolic redox balance.^52^

To contextualize metabolic differences between groups, relative marker abundance was compared using log_2_FC values (Table S9) and the heatmap (Figure 2). In this framework, positive values indicate higher relative abundance in Au NP-treated samples, whereas negative values indicate lower relative abundance than in the control. Absolute log_2_FC values ≥ 1 correspond to differences of at least twofold in normalized signal intensity.^53^ These values were used descriptively to summarize the direction and magnitude of the observed relative signal differences.

**Figure 2:**
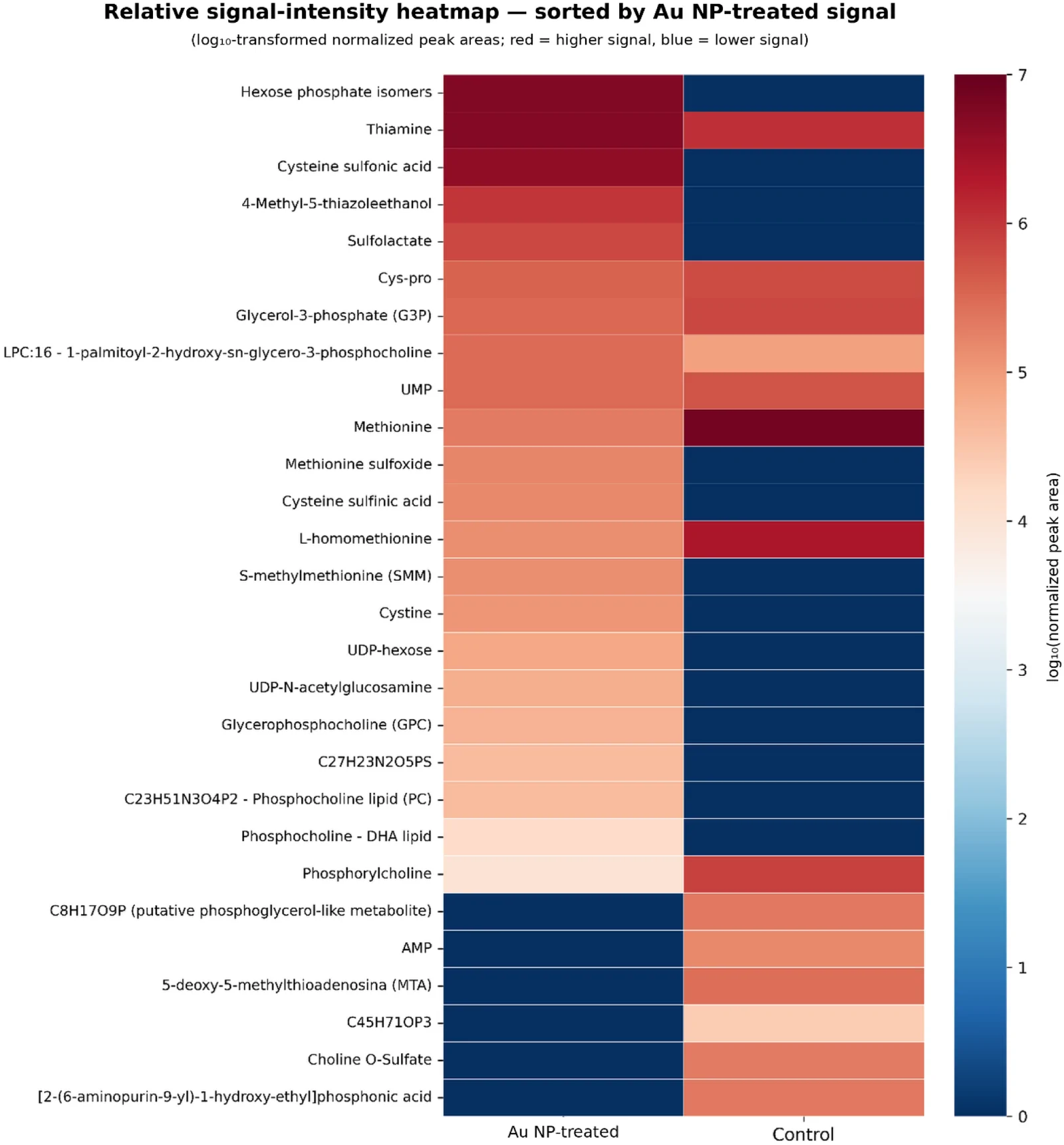
Heatmap of relative LC–HRMS signal intensities for the 28 unique putative S- and/or P-containing metabolite annotations detected in untreated and Au NP-treated soybean callus (100 μg L^–1^). Log_10_-transformed normalized peak areas were collapsed by compound using the maximum signal intensity across ionization modes. Blue shades indicate lower relative signals, whereas red shades indicate higher relative signals. Features that meet the workflow detection and filtering criteria in only one condition, a value of zero was assigned for visualization in the other condition; this does not demonstrate their absolute absence.

Among the features detected in both groups, several exhibited lower relative abundance in the Au NP-treated callus, particularly those putatively annotated as sulfur-containing metabolites. The largest negative log_2_FC values were observed for methionine (log_2_FC = −5.20), l-homomethionine (log_2_FC = −4.10), Cys-Pro (log_2_FC = −0.77), and shared phosphorus metabolites, including phosphorylcholine (log_2_FC = −6.21), G3P (log_2_FC = −1.02), and UMP (log_2_FC = −0.72). These directional changes may reflect greater utilization of reduced S pools and alterations in phospholipid- and nucleotide-related metabolism.^54^ Similar metabolic signatures have been reported in other systems during nanoparticle-induced or metal-mediated stress, where thiol-containing metabolites and membrane precursors participate in responses associated with reactive oxygen species (ROS) buffering and maintenance of cellular structure and function.^55–57^

Among the shared P-containing metabolite annotations, phosphorylcholine showed the largest negative shift in relative abundance (log_2_FC = −6.21). Because this compound feeds the Kennedy pathway, its lower normalized signal is consistent with altered phosphatidylcholine synthesis and membrane turnover in the Au NP-treated callus. Smaller negative shifts in relative abundance were observed for G3P (log_2_FC = −1.02) and UMP (log_2_FC = −0.72). These metabolites occupy key metabolic junctions: G3P links glycolysis and lipid metabolism, and UMP supports nucleotide turnover. Therefore, these directional patterns suggest broader adjustments in P-related metabolism under Au NP exposure.^58,59^ These observations represent qualitative and relative shifts in the metabolic profile and do not support absolute or strictly quantitative interpretation. Follow-up targeted experiments with controlled replication and absolute quantification will be required to confirm the metabolic trends identified here.

In contrast, thiamine showed a higher relative signal in the Au NP-treated callus (log_2_FC = +2.13). This positive shift is consistent with thiamine’s role in antioxidant responses and abiotic-stress tolerance.^60,61^ LPC:16 also showed a higher relative signal in the Au NP-treated callus (log_2_FC = +1.82), suggesting responses related to membrane remodeling and phospholipase-dependent phosphatidylcholine turnover.^50,51^ Taken together, these relative signal differences suggest that Au NP exposure was associated with changes in several core biochemical processes in soybean callus. Relative signals for reduced sulfur metabolites and key phospholipid precursors were lower, whereas thiamine and LPC:16 showed higher relative signals. This metabolite pattern suggests that the response was not restricted to a single pathway. Examining the compounds shared by both samples helps illustrate this response and provides insight into possible metabolic redirection in soybean callus under Au NP exposure.^7,62^

To our knowledge, the specific pattern of P- and S-containing relative signal differences observed here has not previously been described in Au NP-exposed soybean callus. Nevertheless, recent work in soybean showed that Au NP exposure altered root architecture and selectively reconfigured nutrient homeostasis despite limited Au accumulation.^5^ Similarly, Ag NP treatments in tomato increased foliar concentrations of N, P, and K and altered root architecture.^63^ Together, these findings support the interpretation that metal-based NPs can affect nutrient status and related physiological processes in plants. Figure S46 in the Supporting Information (Section 3) summarizes the principal relative signal patterns for the P- and S-containing putative metabolite annotations detected in untreated and Au NP-treated callus, together with the proposed metabolic modules associated with Au NP exposure.

Throughout the following sections, “detected only” indicates that a feature met the workflow detection and filtering criteria in one group but not in the other; it does not demonstrate the absolute absence of that metabolite from the comparison group.

### 3.5 Putatively Annotated P- and S-Containing Features Detected Only in the Control Soybean Callus

Six features with putative metabolite annotations met the detection and filtering criteria only in the control soybean callus, suggesting differences in the relative representation of the associated biochemical processes following Au NP exposure. AMP is a canonical adenylate involved in nucleotide turnover and purine salvage pathways, reflecting its role in energy metabolism and RNA-related processes in proliferating plant tissues.^64^ However, detecting it only under the control-condition criteria does not, by itself, establish reduced purine recycling capacity or decreased adenylate turnover in the Au NP-treated callus. Similarly, 5-deoxy-5-methylthioadenosine (MTA) is a well-established intermediate of the Yang cycle, which connects methionine regeneration with polyamine biosynthesis.^65^ Its detection only in the control profile suggests a difference in the relative representation of methionine-recovery intermediates but does not demonstrate suppression of the pathway after Au NP exposure. Choline O-sulfate is a sulfur-containing osmoprotectant involved in stress mitigation and sulfur storage in some plant species.^66^ Its condition-specific detection may therefore reflect differences in sulfur-associated osmoprotective chemistry, although the present data cannot establish reduced osmoprotection.

The C_8_H_17_O_9_P feature was putatively classified as a phosphoglycerol-like candidate because its molecular formula and MS^2^ behavior were compatible with phosphorylated glycerol derivatives associated with phospholipid assembly or turnover.^67^ To our knowledge, no metabolite with this exact composition and MS^2^ behavior has been reported in plant metabolomics studies or annotated in the PlantCyc and KNApSAcK databases, supporting its classification as a previously unreported putative candidate rather than a definitively identified metabolite.^68,69^ The C_45_H_71_OP_3_ feature may similarly represent a phospholipid-related species associated with membrane remodeling or phosphatidylcholine derivatives.^67,70^ However, because its precise molecular structure was not established, its detection only under the control-condition criteria cannot be interpreted as evidence of impaired phospholipid biosynthesis in the Au NP-treated callus.

The remaining feature was putatively matched to [2-(6-aminopurin-9-yl)-1-hydroxyethyl]­phosphonic acid, which contains a purine base but has no recognized connection to enzymatic pathways in plants.^71^ Although it was not detected in the procedural blank, we found no corresponding plant-metabolic entry in the databases examined.^68,69^ It was therefore treated cautiously as a possible non-biological or exogenous feature and excluded from pathway-level interpretation, without assigning a specific analytical origin.^72^ Overall, the six features detected only under the control-condition criteria may reflect differences in the relative representation of purine phosphate turnover, methionine recycling, sulfur-associated osmoprotection, and phospholipid-related intermediates. Nevertheless, their condition-specific detection alone does not demonstrate pathway suppression or reduced biochemical activity following Au NP exposure.

### 3.6 Putatively Annotated P- and S-Containing Features Detected Only in Au NP-Treated Soybean Callus

The integrated online LC–ESI-HRMS/ICP-MS/MS platform detected 14 S- or P-containing features that met the annotation and filtering criteria only in the Au NP-treated soybean callus under the present analytical conditions. These features were considered within four biochemical contexts: (i) oxidation-related changes in S-containing metabolites; (ii) thiamine/thiazole-associated metabolism; (iii) carbohydrate phosphorylation and nucleotide-sugar metabolism; and (iv) phospholipid-related metabolism. These groupings provide a framework for discussing the condition-specific annotations but do not establish pathway activation or physiological adaptation.

#### 3.6.1 Group 1: Oxidized Sulfur-Containing Metabolites

A notable feature of the Au NP-treated soybean callus profile was the detection of methionine sulfoxide, cysteine sulfinic acid, cysteine sulfonic acid, cystine, and sulfolactate only under the treated-condition criteria. These metabolites relate to known reactions in sulfur redox chemistry. Methionine sulfoxide can be formed through methionine oxidation and participates in reversible redox regulation mediated by methionine sulfoxide reductases.^54^ Cysteine sulfinic and sulfonic acids can result from progressive oxidation of cysteine, whereas cystine is formed through disulfide bond formation between cysteine molecules.^73^ Sulfolactate represents an additional sulfonated metabolite within the treated profile. Collectively, these annotations are consistent with a greater relative representation of oxidized S-containing forms in the Au NP-treated callus.^39,54,73^ However, because ROS levels, antioxidant-enzyme activities, and cellular redox status were not directly measured, this pattern does not demonstrate Au NP-induced ROS production or establish moderate oxidative stress.

Plant responses to metal-based nanoparticles depend on nanoparticle composition, dose, exposure conditions, and the biological system, so they cannot be characterized as uniformly mild or harmful. In soybean, Ag NP exposure has been associated with glutathione-mediated detoxification and changes in nitrogen assimilation.^74^ More recent experimental studies reported changes in redox-related and biochemical responses in Au NP-treated spinach under salinity stress^29^ and concentration-dependent chemical-profile remodeling in transgenic soybean callus exposed to Cu NPs at 10, 50, and 100 μg L^–1^ for 30 days.^17^ These studies provide relevant context for considering the sulfur-containing profile observed here as potentially stress-associated, but they do not provide direct evidence of ROS generation or antioxidant activation in the present experiment.

Because the present study evaluated only one Au NP concentration (100 μg L^–1^), these relative metabolic patterns cannot establish a dose–response relationship or define thresholds between adaptive and adverse effects. In addition, we included no ionic or bulk Au control; therefore, the observed differences cannot be attributed exclusively to the nanoscale form rather than to Au exposure more generally.

#### 3.6.2 Group 2: Thiamine- and Thiazole-Associated Metabolism

The putatively annotated thiazole-related metabolite 4-methyl-5-thiazoleethanol, which is associated with the thiazole moiety of vitamin B_1_ (thiamine) biosynthesis, met the detection and filtering criteria only in the Au NP-treated callus.^75,76^ Thiamine itself was detected in both conditions but showed a higher relative signal in the Au NP-treated callus (log_2_FC = +2.13). In plants, thiamine functions as an essential cofactor in central metabolism and has also been associated with antioxidant responses and tolerance to abiotic stress.^60,61,76^ The condition-specific detection of the thiazole-related feature, together with the higher relative signal of thiamine, is therefore consistent with differences in thiamine-associated metabolism following Au NP exposure. However, these relative data do not demonstrate activation of thiamine biosynthesis, increased regenerative capacity, or improved stress tolerance.

#### 3.6.3 Group 3: Hexose Phosphate- and UDP-Sugar-Associated Metabolism

Three P-containing features, putatively annotated as hexose phosphate isomers, UDP-hexose, and UDP-N-acetylglucosamine, met the detection and filtering criteria only in the Au NP-treated callus. These features are associated with central carbon metabolism, nucleotide-sugar formation, glycosylation, and cell-wall biosynthesis.^77,78^ Hexose phosphate intermediates can participate in glycolytic and pentose phosphate metabolism, including NADPH-generating reactions,^79^ whereas UDP-hexoses provide activated sugar donors for biosynthetic processes such as cell-wall formation.^77^ UDP-N-acetylglucosamine is an activated amino-sugar donor involved in protein and lipid glycosylation and cell-wall components in plants.^78^

In soybean, callus formation involves extensive transcriptional reprogramming of genes associated with cell-wall modification and the cell cycle, and changes involving cell-wall genes and composition have also been reported during callus development in other plant systems.^80,81^ Therefore, the condition-specific detection of these three features is consistent with differences in hexose phosphate- and UDP-sugar-associated metabolism following Au NP exposure. However, the present relative data do not establish increased pentose phosphate pathway flux, enhanced NADPH generation, greater glycosylation activity, cell-wall reinforcement, or increased cell division.

#### 3.6.4 Group 4: Membrane Phospholipid-Associated Metabolism

The P-containing features putatively annotated as a DHA-like phosphocholine lipid, PC-like phosphocholine derivatives, and glycerophosphocholine (GPC) met the detection and filtering criteria only in the Au NP-treated callus. In plants, phosphatidylcholine turnover can involve phospholipase-mediated cleavage and the formation of lysophospholipids and GPC.^51,82^ Changes in membrane lipid composition and unsaturation are also well-described components of plant stress responses and may influence membrane properties and lipid-derived signaling.^51,83^ Therefore, the condition-specific detection of these features is consistent with differences in phosphatidylcholine-associated metabolism and membrane phospholipid remodeling following Au NP exposure. However, these relative data do not demonstrate activation of specific phospholipases, increased production of lysolipids or GPC, changes in membrane fluidity, or generation of bioactive lipids.

#### 3.6.5 Unassigned Aromatic P- and S-Containing Feature (C_27_H_23_N_2_O_5_PS)

One P- and S-containing feature with the proposed molecular formula C_27_H_23_N_2_O_5_PS met the detection and filtering criteria only in the Au NP-treated callus. The precursor ion at m/z 519.114 co-eluted with both P and S ICP-MS/MS signals, while its high degree of unsaturation (DBE = 18) and MS^2^ fragmentation pattern were consistent with an aromatic structure (Figure S33 in the Supporting Information). However, the available data were insufficient to assign this feature to a known endogenous plant metabolite or to determine its source. Consistent with the recommended caution in interpreting unassigned LC–MS signals,^72,84^ this feature was classified as unassigned and excluded from pathway-level biological interpretation. Its detection only in the Au NP-treated sample does not show that Au NPs generated it or that it resulted from a surface-mediated chemical reaction. A schematic summary of the observed S- and P-containing metabolite signal patterns in soybean callus exposed to Au NPs, compared with the untreated control, and the proposed literature-informed mechanistic hypotheses is presented in Figure 3.

**Figure 3:**
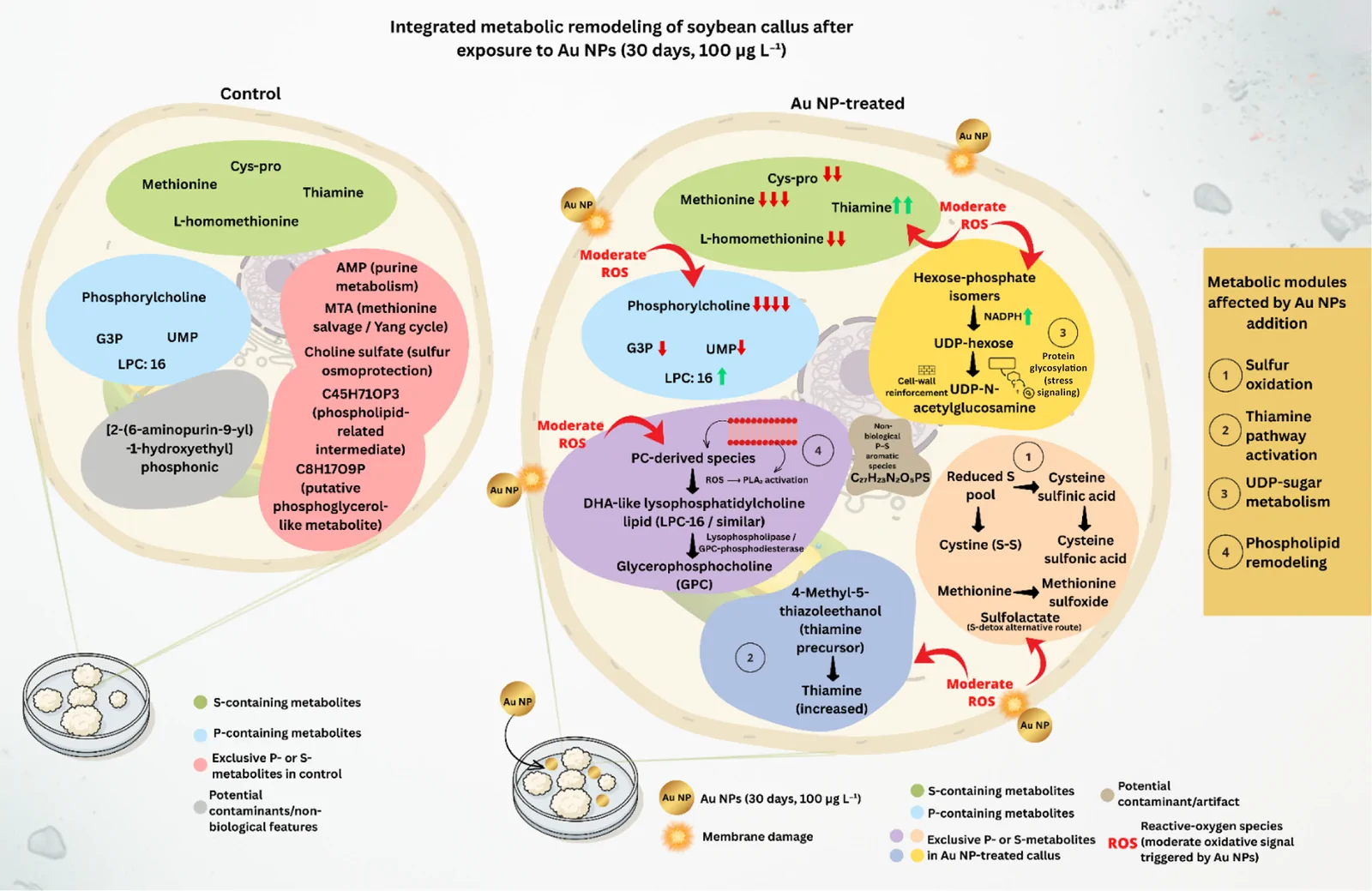
Schematic summary of S- and P-containing metabolite signal patterns in soybean callus associated with Au NP exposure (100 μg L^–1^, 30 days). Red and green arrows indicate, respectively, lower and higher relative LC–HRMS signals in the Au NP-treated analytical profile compared with the untreated control. Compounds shown in only one condition represent putative metabolite annotations detected only in that analytical profile, according to the established detection and filtering criteria. These relative signal differences and condition-specific detections were analytically observed. In contrast, the depicted relationships involving ROS, membrane effects, enzymatic activity, pathway activation, and metabolic flux are literature-informed mechanistic hypotheses proposed to contextualize the analytical findings; these processes were not directly measured and should not be interpreted as evidence of causality.

#### 3.6.6 Secondary Metabolites of Particular Significance

Among the features that met the detection and filtering criteria only in the Au NP-treated sample, those putatively annotated as 4-methyl-5-thiazoleethanol, S-methylmethionine (SMM), and PC-like/DHA-like lipids are of particular interpretive interest because they are associated with thiamine-related, sulfur-containing amino acid, and phospholipid metabolism, respectively.^43,51,75,76,83^ Considered together, their condition-specific detection is consistent with differences across these metabolic processes following Au NP exposure. However, these features should not be interpreted as established biomarkers of adaptive stress tolerance, membrane reinforcement, antioxidant preparedness, or improved resilience. Table S10 summarizes their putative biochemical classes, reported functions in plants, and possible metabolic associations.

Overall, the online multimodal HILIC–ESI-HRMS/ICP-MS/MS workflow provided complementary molecular and element-specific evidence for 38 ion/adduct features corresponding to 28 unique putative P- and/or S-containing annotations in soybean callus. MS^1^ and MS^2^ data supported the molecular annotation, while chromatographic co-elution with P- or S-ICP-MS/MS traces strengthened the elemental association. Because authentic standards were not used for most annotations, the assignments remain putative, and the profiles should be interpreted qualitatively and relatively rather than as definitive identifications or absolute quantification.

Across the two conditions, eight unique annotations were shared. In the Au NP-treated profile, methionine, l-homomethionine, and phosphorylcholine showed lower relative signals, whereas thiamine and LPC:16 showed higher relative signals. Oxidized S-containing metabolites and thiazole-, UDP-sugar-, PC-like-, and GPC-related features met the detection and filtering criteria only in the treated sample. Collectively, these patterns are consistent with exposure-associated differences in sulfur redox, thiamine-associated, nucleotide-sugar, and membrane phospholipid metabolism. They do not, however, establish pathway activation, metabolic flux, hormesis, enhanced antioxidant capacity, or improved stress tolerance.

Soybean callus provides a controlled cellular system for examining element-associated biochemical responses but lacks differentiated organs, root uptake, vascular transport, soil interactions, and whole-plant physiology. Moreover, the present study evaluated a single Au NP concentration, included no ionic or bulk Au control, and did not assess post-exposure anatomical or physiological end points. Consequently, these findings cannot be directly extrapolated to greenhouse- or field-grown soybean. Au NPs were therefore used here as a model nanomaterial for probing P- and S-associated metabolic patterns rather than as a proposed agricultural input. Future dose-response studies incorporating appropriate Au controls, post-exposure physiological measurements, and whole-plant systems will be required to evaluate the reproducibility and agricultural relevance of these observations.

## Supplementary Material



## Data Availability

The computational workflows used for MS^1^ screening, isotopic validation, MS^2^ confirmation, and LC–HRMS × ICP-MS/MS multimodal integration have been deposited in Zenodo at DOI: 10.5281/zenodo.20529691. Processed datasets supporting the findings of this study are provided in the Supporting Information. Additional data, materials, and computational resources are available from the corresponding author upon reasonable request.

## Notes

Additional experimental details, anatomical characterization of soybean callus, instrumental parameters, quality-control procedures, lag-correction methodology, blank analysis, annotated MS/MS spectra, HRMS × ICP-MS/MS chromatographic overlays, metabolite annotation-support tables, relative-signal summaries, and supporting discussion (Figures S1–S46; Tables S1–S10) (PDF)

## References

[1] Salam A., Khan A. R., Zeeshan M., Afridi M. S., Yang L., Zheng Q., Ulhassan Z., Wang R., Zhang Z., Zhao C. (2026). Nano-Enabled Solutions for Plant Abiotic Stress Tolerance and Soil
Contaminant Remediation: A Review. Plants.

[2] Francis D. V., Abdalla A. K., Mahakham W., Sarmah A. K., Ahmed Z. F. R. (2024). Interaction
of Plants and Metal Nanoparticles: Exploring Its Molecular Mechanisms
for Sustainable Agriculture and Crop Improvement. Environ. Int..

[3] Wei L., Liu J., Jiang G. (2024). Nanoparticle-Specific Transformations
Dictate Nanoparticle
Effects Associated with Plants and Implications for Nanotechnology
Use in Agriculture. Nat. Commun..

[4] Venzhik Y., Deryabin A., Naraikina N., Zhukova K., Dykman L. (2024). The Influence
of Au-Based Nanoparticles on Some Physiological, Biochemical and Molecular
Characteristics of Wheat Plants during Low Temperature Hardening. Plant Physiol. Biochem..

[5] Fonseca E. K. B., Kato L. S., de Barros A., de Andrade A. M., Mazali I. O., Arruda M. A. Z. (2026). Gold Nanoparticles Modulate Root
Architecture and Nutrient Homeostasis During Early Soybean Growth. J. Plant Growth Regul.

[6] Mahakham W., Theerakulpisut P., Maensiri S., Phumying S., Sarmah A. K. (2016). Environmentally
Benign Synthesis of Phytochemicals-Capped Gold Nanoparticles as Nanopriming
Agent for Promoting Maize Seed Germination. Sci. Total Environ..

[7] Kumar V., Guleria P., Kumar V., Yadav S. K. (2013). Gold Nanoparticle
Exposure Induces Growth and Yield Enhancement in Arabidopsis Thaliana. Sci. Total Environ..

[8] Avellan A., Yun J., Zhang Y., Spielman-Sun E., Unrine J. M., Thieme J., Li J., Lombi E., Bland G., Lowry G. V. (2019). Nanoparticle Size
and Coating Chemistry Control Foliar Uptake Pathways, Translocation,
and Leaf-to-Rhizosphere Transport in Wheat. ACS Nano.

[9] Nemček L., Šebesta M., Afzal S., Bahelková M., Vaculovič T., Kollár J., Mat́ko M., Hagarová I. (2026). Exploratory LA-ICP-MS Imaging of Foliar-Applied Gold
Nanoparticles and Nutrients in Lentil Leaves. Appl. Sci..

[10] Godel-Jędrychowska K., Milewska-Hendel A., Sala K., Barański R., Kurczyńska E. (2023). The Impact
of Gold Nanoparticles on Somatic Embryogenesis
Using the Example of Arabidopsis Thaliana. Int.
J. Mol. Sci..

[11] Rana, A. ; Dayal, S. ; Kumar, A. ; Tiwari, S. ; Siddiqui, M. A. Gold Nanomaterials in Plant Tissue Culture: Effects and Challenges. In Exploring Nanotechnology for In Vitro Plant Improvement: Nanoparticles in Plant Tissue Culture Systems, Husen, A. ; Pant, M. ; Springer Nature Singapore: Singapore, 2026; pp. 211–237. 10.1007/978-981-95-8765-0_9

[12] Semenova N. A., Zakharov D. A., Serov D. A., Gudkov S. V., Dorokhov A. S., Izmailov A. Y. (2026). Application of Nanoparticles in Plant In Vitro Culture
for Micropropagation and Secondary Metabolite Production: A Review. Plants.

[13] Ikeuchi M., Sugimoto K., Iwase A. (2013). Plant Callus: Mechanisms of Induction
and Repression. Plant Cell.

[14] Aurélio
Zezzi Arruda M., Beatriz Santos da Silva A., Seiko Kato L. (2023). There Is Plenty
of Room in Plant Science: Nanobiotechnology as an Emerging Area Applied
to Somatic Embryogenesis. J. Agric. Food Chem..

[15] Husak V., Faltus M., Bilavcik A., Narozhnyi S., Bobrova O. (2026). Nanoparticle Applications in Plant Biotechnology: A
Comprehensive Review. Plants.

[16] Fazal H., Abbasi B. H., Ahmad N., Ali M. (2016). Elicitation of Medicinally
Important Antioxidant Secondary Metabolites with Silver and Gold Nanoparticles
in Callus Cultures of Prunella Vulgaris L. Appl.
Biochem. Biotechnol.

[17] Gamela R. R., Barbosa Fonseca E. K., Cerqueira da Silva V.
H., Schmitt C. R., Arruda M. A. Z. (2026). Integrated Online HILIC-ESI-HRMS and ICP-MS/MS for
Chemical Species Profiling in Transgenic Soybean Callus Exposed to
Copper Nanoparticles. Anal. Bioanal. Chem..

[18] Kokina I., Jahundoviča I., Mickeviča I., Jermalonoka M., Strautiņš J., Popovs S., Ogurcovs A., Sledevskis E., Polyakov B., Gerbreders V. (2017). Target Transportation
of Auxin on Mesoporous Au/SiO2 Nanoparticles as a Method for Somaclonal
Variation Increasing in Flax (L. Usitatissimum L.). J. Nanomater.

[20] Khan K., Khan R., Liu Z., Ali S., Naseer M. A., Shah M. A., Ahmad H., Zhou X. B. (2025). Melatonin Mitigates
Nickel Oxide Nanoparticles Induced Phytotoxicity in Soybean by Reducing
Metal Accumulation, Enhancing Antioxidant Defense and Promoting Nitrogen
Assimilation. J. Hazard. Mater..

[21] Sirko A., Wawrzyńska A., Olszak M., Piotrowska J., Sieńko M. (2026). Hidden Players
in Plant Response to Sulfur Deficit
and beyond: Insights into the Function of the Proteins Sulfur Deficiency
Induced and Response to Low Sulfur. J. Exp.
Bot.

[22] Chen J., Zhou J., Li M., Li M., Hu Y., Zhang T., Shi L. (2022). Membrane Lipid Phosphorus
Reusing
and Antioxidant Protecting Played Key Roles in Wild Soybean Resistance
to Phosphorus Deficiency Compared with Cultivated Soybean. Plant Soil.

[23] Khan F., Siddique A. B., Shabala S., Zhou M., Zhao C. (2023). Phosphorus
Plays Key Roles in Regulating Plants’ Physiological Responses
to Abiotic Stresses. Plants.

[24] Raab A., Ronzan M., Feldmann J. (2017). Sulphur Fertilization
Influences
the Sulphur Species Composition in Allium Sativum: Sulphomics Using
HPLC-ICPMS/MS-ESI-MS/MS. Metallomics.

[25] Kato L. S., Cerqueira da Silva V. H., Campaci de Andrade D., Cruz G., Pedrobom J. H., Raab A., Feldmann J., Arruda M. A. Z. (2024). Multimodal Chemical Speciation Techniques
Based on
Simultaneous High Resolution Molecular/Atomic Mass Spectrometry Applied
to Online Target/Non-Target Analysis: A Tutorial Review. Anal. Chim. Acta.

[26] Feldmann J., Hansen H. R., Karlsson T. M., Christensen J. H. (2024). ICP-MS
As a Contributing Tool to Nontarget Screening (NTS) Analysis for Environmental
Monitoring. Environ. Sci. Technol..

[27] Arruda M. A. Z., de Jesus J. R., Blindauer C. A., Stewart A. J. (2022). Speciomics as a
Concept Involving Chemical Speciation and Omics. J. Proteomics.

[28] Joshi S., Dar A. I., Acharya A., Joshi R. (2022). Charged Gold Nanoparticles
Promote In Vitro Proliferation in Nardostachys Jatamansi by Differentially
Regulating Chlorophyll Content, Hormone Concentration, and Antioxidant
Activity. Antioxidants.

[29] Amir M., Raheem A., Yadav P., Kumar V., Tewari R. K., Jalil S. U., Danish M., Ansari M. I. (2024). Phytofabricated
Gold Nanoparticles as Modulators of Salt Stress Responses in Spinach:
Implications for Redox Homeostasis, Biochemical and Physiological
Adaptation. Front. Plant Sci..

[30] Alabdallah N. M. (2025). Do Gold
Nanoparticles Consistently Bene Fi t Crop Plants under Both Non-Stressed
and Abiotic Stress Conditions?. Nanotechnol.
Rev..

[31] Sultana M. S., Frazier T. P., Millwood R. J., Lenaghan S. C., Stewart C. N. (2019). Development
and Validation of a Novel and Robust Cell Culture System in Soybean
(Glycine Max (L.) Merr.) for Promoter Screening. Plant Cell Rep..

[32] Ferrari I. F., Marques G. A., Junior W. L. S., Biazotti B. B., Pena
Passos M., de Almeida J. A. S., Mondego J. M. C., Mayer J. L. S. (2021). Comparative
Ontogenesis of Coffea Arabica L. Somatic Embryos Reveals the Efficiency
of Regeneration Modulated by the Explant Source and the Embryogenesis
Pathway. Vitr. Cell. Dev. Biol. - Plant.

[33] Fonseca, E. K. B. ; Kato, L. S. ; de Barros, A. ; Mazali, I. O. ; Arruda, M. A. Z. Ionomic Response Increment on the Soybean Callus Exposed to Low Concentration of Gold Nanoparticles. In Book Of Abstracts, 21st Brazilian Meeting On Analytical Chemistry And 9th Ibero-American Congress Of Analytical Chemistry; Brazilian Chemical Society: Belém, 2024.

[34] Flis P., Ouerdane L., Grillet L., Curie C., Mari S., Lobinski R. (2016). Inventory of Metal
Complexes Circulating in Plant Fluids:
A Reliable Method Based on HPLC Coupled with Dual Elemental and High-Resolution
Molecular Mass Spectrometric Detection. New
Phytol..

[35] Kato, L. S. Multimodal LC–HRMS × ICP–MS/MS Data Processing Workflows (Scripts 1–3 and MS^2^ Batch Validation); zenodo.

[36] George, E. F. ; Hall, M. A. ; De Klerk, G.-J. The Anatomy and Morphology of Tissue Cultured Plants. In Plant Propagation by Tissue Culture vol 1, The Background George, E. F. ; Hall, M. A. ; Klerk, G.-J. De , Eds.; Springer Dordrecht: Netherlands, 2008; pp. 465–477. 10.1007/978-1-4020-5005-3_13

[37] da
Silva A. B. S., Arruda M. A. Z. (2023). Exploring Single-Particle ICP-MS
as an Important Tool for the Characterization and Quantification of
Silver Nanoparticles in a Soybean Cell Culture. Spectrochim. Acta - Part B At. Spectrosc..

[38] Hu Q., Tang H., Wang Y. (2020). Challenges in Analysis of Hydrophilic
Metabolites Using Chromatography Coupled with Mass Spectrometry. J. Anal. Test.

[39] Takahashi H., Marsolais F., Cuypers A., Kopriva S. (2023). Sulfur Metabolism:
Actions for Plant Resilience and Environmental Adaptation. J. Exper. Botany.

[40] Noctor G., Foyer C. H. (2016). Intracellular Redox Compartmentation and ROS-Related
Communication in Regulation and Signaling. Plant
Physiol..

[41] Takahashi H., Kopriva S., Giordano M., Saito K., Hell R. (2011). Sulfur Assimilation
in Photosynthetic Organisms: Molecular Functions and Regulations of
Transporters and Assimilatory Enzymes. Annu.
Rev. Plant Biol..

[42] Tunc-Ozdemir M., Miller G., Song L., Kim J., Sodek A., Koussevitzky S., Misra A. N., Mittler R., Shintani D. (2009). Thiamin Confers
Enhanced Tolerance to Oxidative Stress in Arabidopsis. Plant Physiol..

[43] Amir R. (2010). Current Understanding
of the Factors Regulating Methionine Content in Vegetative Tissues
of Higher Plants. Amino Acids.

[44] Yi H., Ravilious G. E., Galant A., Krishnan H. B., Jez J. M. (2010). From Sulfur
to Homoglutathione: Thiol Metabolism in Soybean. Amino Acids.

[45] Stigter K. A., Plaxton W. C. (2015). Molecular Mechanisms of Phosphorus
Metabolism and Transport
during Leaf Senescence. Plants.

[46] Vance C. P., Uhde-Stone C., Allan D. L. (2003). Phosphorus Acquisition and Use: Critical
Adaptations by Plants for Securing a Nonrenewable Resource. New Phytol..

[47] Ren J., Lin J., Yu L., Yan M. (2022). Lysophosphatidylcholine: Potential
Target for the Treatment of Chronic Pain. Int.
J. Mol. Sci..

[48] Nakamura Y. (2013). Phosphate
Starvation and Membrane Lipid Remodeling in Seed Plants. Prog. Lipid Res..

[49] Welti R., Li W., Li M., Sang Y., Biesiada H., Zhou H.-E., Rajashekar C. B., Williams T. D., Wang X. (2002). Profiling Membrane
Lipids in Plant Stress Responses. Role of Phospholipase D Alpha in
Freezing-Induced Lipid Changes in Arabidopsis. J. Biol. Chem..

[50] Hou Q., Ufer G., Bartels D. (2016). Lipid Signalling
in Plant Responses
to Abiotic Stress. Plant, Cell Environ..

[51] Yu L., Zhou C., Fan J., Shanklin J., Xu C. (2021). Mechanisms
and Functions of Membrane Lipid Remodeling in Plants. Plant J..

[52] Venugopal S. C., Chanda B., Vaillancourt L., Kachroo A., Kachroo P. (2009). The Common
Metabolite Glycerol-3-Phosphate Is a Novel Regulator of Plant Defense
Signaling. Plant. Signal. Behav..

[53] Chong J., Wishart D. S., Xia J. (2019). Using MetaboAnalyst
4.0 for Comprehensive
and Integrative Metabolomics Data Analysis. Curr. Protoc. Bioinf.

[54] Rey P., Tarrago L. (2018). Physiological Roles
of Plant Methionine Sulfoxide Reductases
in Redox Homeostasis and Signaling. Antioxidants.

[55] Noctor G., Mhamdi A., Foyer C. H. (2014). The Roles
of Reactive Oxygen Metabolism
in Drought: Not so Cut and Dried. Plant Phys..

[56] Hossain, M. A. ; Wani, S. H. ; Bhattacharjee, S. ; Burritl, D. J. ; Tran, L. S. P. Molecular and Genetic Perspectives In Drought Tolerance in Plants, Vol 2., Springer: Cham, Switzerland, 2016 May 1–526 10.1007/978-3-319-28899-4

[57] Yang J., Cao W., Rui Y. (2017). Interactions between Nanoparticles and Plants: Phytotoxicity
and Defense Mechanisms. J. Plant Interact.

[58] Hildebrandt T. M., Nunes Nesi A., Araújo W. L., Braun H.-P. (2015). Amino Acid Catabolism
in Plants. Mol. Plant.

[59] Zrenner R., Stitt M., Sonnewald U., Boldt R. (2006). Pyrimidine and Purine
Biosynthesis and Degradation in Plants. Annu.
Rev. Plant Biol..

[60] Li W., Mi X., Jin X., Zhang D., Zhu G., Shang X., Zhang D., Guo W. (2022). Thiamine Functions as a Key Activator
for Modulating Plant Health and Broad-Spectrum Tolerance in Cotton. Plant J..

[61] Atif M., Perveen S., Parveen A., Mahmood S., Saeed M., Zafar S. (2022). Thiamine and Indole-3-Acetic Acid Induced Modulations in Physiological
and Biochemical Characteristics of Maize (Zea Mays L.) under Arsenic
Stress. Sustain.

[62] Landa P. (2021). Positive Effects
of Metallic Nanoparticles on Plants: Overview of Involved Mechanisms. Plant Physiol. Biochem..

[63] Guzmán-Báez G. A., Trejo-Téllez L. I., Ramírez-Olvera S. M., Salinas-Ruíz J., Bello-Bello J. J., Alcántar-González G., Hidalgo-Contreras J. V., Gómez-Merino F.
C. (2021). Silver Nanoparticles
Increase Nitrogen, Phosphorus, and Potassium Concentrations in Leaves
and Stimulate Root Length and Number of Roots in Tomato Seedlings
in a Hormetic Manner. Dose-Response.

[64] Ashihara H., Stasolla C., Fujimura T., Crozier A. (2018). Purine Salvage in Plants. Phytochemistry.

[65] Bürstenbinder K., Waduwara I., Schoor S., Moffatt B. A., Wirtz M., Minocha S. C., Oppermann Y., Bouchereau A., Hell R., Sauter M. (2010). Inhibition
of 5’-Methylthioadenosine
Metabolism in the Yang Cycle Alters Polyamine Levels, and Impairs
Seedling Growth and Reproduction in Arabidopsis. Plant J..

[66] Rivoal J., Hanson A. D. (1994). Choline-O-Sulfate
Biosynthesis in Plants (Identification
and Partial Characterization of a Salinity-Inducible Choline Sulfotransferase
from Species of Limonium (Plumbaginaceae). Plant
Phys..

[67] Nakamura Y. (2017). Plant Phospholipid
Diversity: Emerging Functions in Metabolism and Protein-Lipid Interactions. Trends Plant Sci..

[68] Hawkins C., Xue B., Yasmin F., Wyatt G., Zerbe P., Rhee S. Y. (2025). Plant Metabolic
Network 16: Expansion of Underrepresented Plant Groups and Experimentally
Supported Enzyme Data. Nucleic Acids Res..

[69] Afendi F. M., Okada T., Yamazaki M., Hirai-Morita A., Nakamura Y., Nakamura K., Ikeda S., Takahashi H., Altaf-Ul-Amin M., Darusman L. K., Saito K., Kanaya S. (2012). KNApSAcK Family
Databases: Integrated Metabolite-Plant Species Databases for Multifaceted
Plant Research. Plant Cell Phys..

[70] Li-Beisson Y., Shorrosh B., Beisson F., Andersson M. X., Arondel V., Bates P. D., Baud S., Bird D., Debono A., Durrett T. P., Franke R. B., Graham I. A., Katayama K., Kelly A. A., Larson T., Markham J. E., Miquel M., Molina I., Nishida I., Rowland O., Samuels L., Schmid K. M., Wada H., Welti R., Xu C., Zallot R., Ohlrogge (2013). J. Acyl-Lipid Metabolism. Arab. B.

[71] National Center for Biotechnology Information PubChem Compound Summary for CID 513614, [2-(6-Aminopurin-9-Yl)-1-Hydroxy-Ethyl]Phosphonic Acid; 2025. https://pubchem.ncbi.nlm.nih.gov/compound/513614.

[72] Rakusanova S., Cajka T. (2024). Tips and Tricks for
LC–MS-Based Metabolomics and Lipidomics
Analysis. TrAC, Trends Anal. Chem..

[73] Couturier J., Chibani K., Jacquot J.-P., Rouhier N. (2013). Cysteine-Based Redox
Regulation and Signaling in Plants. Front. Plant
Sci..

[74] Ma C., Liu H., Chen G., Zhao Q., Guo H., Minocha R., Long S., Tang Y., Saad E. M., DeLatorreroche R., White J. C., Xing B., Dhankher O. P. (2020). Dual Roles of Glutathione
in Silver Nanoparticle Detoxification and Enhancement of Nitrogen
Assimilation in Soybean (Glycine Max (L.) Merrill). Environ. Sci.: Nano.

[75] Chatterjee A., Jurgenson C. T., Schroeder F. C., Ealick S. E., Begley T. P. (2007). Biosynthesis
of Thiamin Thiazole in Eukaryotes: Conversion of NAD to an Advanced
Intermediate. J. Am. Chem. Soc..

[76] Fitzpatrick T. B., Chapman L. M. (2020). The Importance
of Thiamine (Vitamin B1) in Plant Health:
From Crop Yield to Biofortification. J. Biol.
Chem..

[77] Verbančič J., Lunn J. E., Stitt M., Persson S. (2018). Carbon Supply and the
Regulation of Cell Wall Synthesis. Mol. Plant.

[78] Chen Y. H., Cheng W. H. (2024). Hexosamine Biosynthesis and Related Pathways, Protein
N-Glycosylation and O-GlcNAcylation: Their Interconnection and Role
in Plants. Front. Plant Sci..

[79] Jiang Z., Wang M., Nicolas M., Ogé L., Pérez-Garcia M.-D., Crespel L., Li G., Ding Y., Le Gourrierec J., Grappin P., Sakr S. (2022). Glucose-6-Phosphate
Dehydrogenases: The Hidden Players of Plant Physiology. Int. J. Mol. Sci..

[80] Park J.-S., Choi Y., Jeong M.-G., Jeong Y.-I., Han J.-H., Choi H.-K. (2023). Uncovering Transcriptional Reprogramming during Callus
Development in Soybean: Insights and Implications. Front. Plant Sci..

[81] Tomasiak A., Piński A., Milewska-Hendel A., Andreu Godall I., Borowska-Żuchowska N., Morończyk J., Moreno-Romero J., Betekhtin A. (2024). H3K4me3 Changes Occur in Cell Wall
Genes during the Development of Fagopyrum Tataricum Morphogenic and
Non-Morphogenic Calli. Front. Plant Sci..

[82] van
der Rest B., Boisson A.-M., Gout E., Bligny R., Douce R. (2002). Glycerophosphocholine Metabolism in Higher Plant Cells. Evidence
of a New Glyceryl-Phosphodiester Phosphodiesterase. Plant Phys..

[83] Guo Q., Liu L., Rupasinghe T. W. T., Roessner U., Barkla B. J. (2022). Salt Stress
Alters Membrane Lipid Content and Lipid Biosynthesis Pathways in the
Plasma Membrane and Tonoplast. Plant Phys..

[84] Bonner R., Hopfgartner G. (2022). The Origin and Implications of Artifact Ions in Bioanalytical
LC–MS. LCGC Suppl..

