# Supplementary material for: Online Multimodal HILIC–ESI-HRMS/ICP-MS/MS Speciation Reveals Remodeling of Sulfur- and Phosphorus-Containing Metabolites Associated with Gold Nanoparticle Exposure in Transgenic (cp4 EPSPS Gene) Soybean Callus
Source: J Agric Food Chem. 2026 Oct 6;74(44):35198–211. doi: 10.1021/acs.jafc.6c09256 (PMC13761271; doi:10.1021/acs.jafc.6c09256)
Supplement: Supplementary file 1 [file jf6c09256_si_001.pdf]

# Online multimodal HILIC–ESI-HRMS/ICP-MS/MS speciation reveals remodeling of sulfur- and phosphorus-containing metabolites associated with gold nanoparticle exposure in transgenic (*cp*4 EPSPS gene) soybean callus

Lilian Seiko Kato*[a], Elisânia Kelly Barbosa Fonseca[a], Vinnícius Henrique Cerqueira da Silva[a], Diego Campaci de Andrade[b], Guilherme Crispim de Faria Cruz[b], Jorge Henrique Pedrobom[c], Marco Aurélio Zezzi Arruda**[a]

[a] Spectrometry, Sample Preparation and Mechanization Group, Institute of Chemistry and National Institute of Science and Technology for Bioanalytics, Institute of Chemistry, University of Campinas (Unicamp), Campinas, São Paulo, 13083-970, Brazil

[b] Institutional Mass Spectrometry and Chromatography Laboratory, Institute of Chemistry, University of Campinas (Unicamp), Campinas, São Paulo, 13083-970, Brazil

[c] Nova Analítica Company, Diadema, SP, 09941-202, Brazil

*Corresponding author: lskato@unicamp.br, ORCID: https://orcid.org/0000-0001-9408-8127

**Corresponding author: zezzi@unicamp.br, ORCID: https://orcid.org/0000-0002-7058-3390

1. MATERIAL AND METHODS

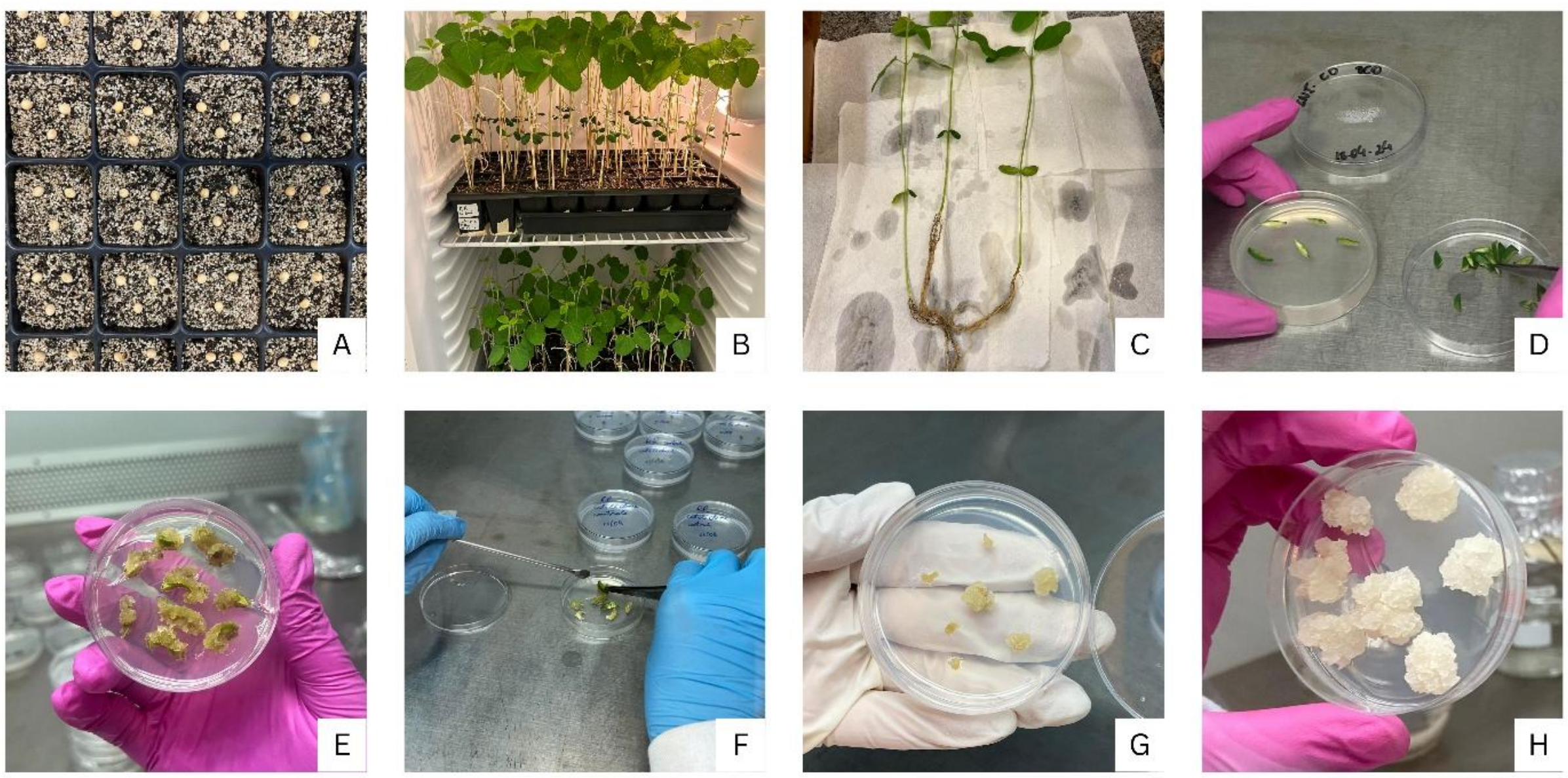


**Figure S1.** The main steps involved in cultivating soybean callus are as follows. A. Seeds of the cultivar RR are planted in a mixture of vermiculite and substrate. B. 21-day-old soybean plants grown in BOD, 12 h light/12 h dark photoperiod. C. Thinning of plants for tissue cutting. D. Preparation of callus induction culture medium, cut, and induced soybean cotyledon pieces. E. Callus growth under the cotyledon tissue. F. Separation and collection of the callus that forms under the piece of tissue. G. Primary callus placed on proliferation medium. H. Grown and mature soybean callus.

## 1.1. Au NP exposure

The Au NP exposure procedure is described in the Materials and Methods section of the main manuscript. Figure S2 shows the untreated and Au NP-treated calli after 30 days.

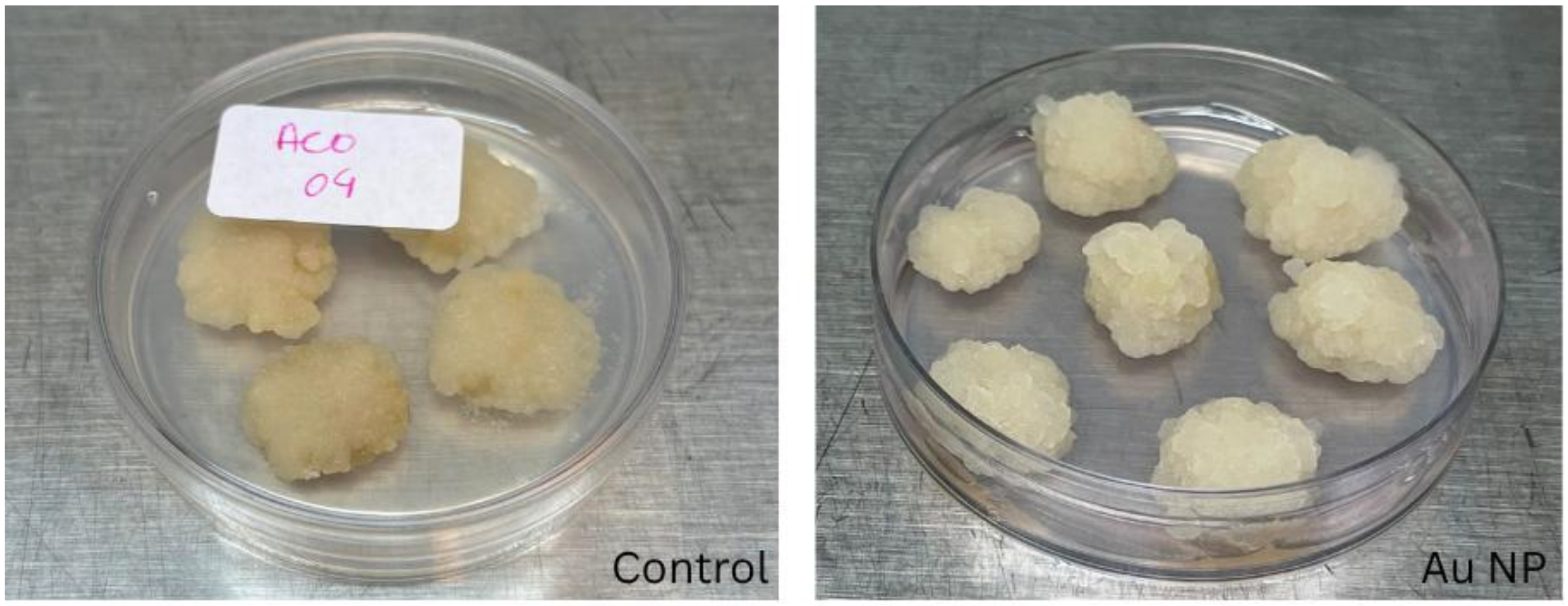


**Figure S2**. Untreated control soybean calli and soybean calli exposed to 100 µg $L^{-1}$ Au NPs for 30 days.

## 1.2. Sample preparation for analysis

Following a previous sample-preparation study that evaluated different extraction solutions and Cup-Horn ultrasound, mature calli from 10 Petri dishes per experimental condition, with approximately seven calli per dish, were stored at −80 °C. For each condition, we combined all calli, immersed them in liquid nitrogen, and ground them in a porcelain crucible. We weighed 200 mg of each homogenized pool into an Eppendorf tube and added 1.5 mL of acetonitrile/water (50:50, v/v). We added glass beads to assist extraction and AsB at 50 µg $L^{-1}$ as an internal standard for quality control. The samples were vortexed for 1 min, subjected to Cup-Horn ultrasound for 90 s at 50% amplitude using 10 s on/off cycles, centrifuged at 8500 rpm for 10 min, filtered through PVDF filters (0.22 µm, 25 mm diameter, Millex-GV, Millipore), and stored under refrigeration until analysis. The comparative results reported here correspond to one processed analytical profile from each pooled experimental condition. A procedural blank was processed in parallel using the same extraction protocol, including reagents, glass beads, sonication, centrifugation, filtration, and LC injection, but without biological material. This procedure assessed potential background signals and carryover. Features were excluded when a corresponding signal in the blank met the same retention-time, mass-accuracy, and, when available, $MS^2$ and elemental co-elution criteria applied to the samples.

## 1.3. Optimization of flow splitting and PEEK lengths

A split-flow valve (Series 600 valve, ASI Company, USA) connected the LC to the ICP-MS/MS and the high-resolution mass spectrometer with electrospray ionization Thermo Orbitrap QExactive (ESI-HRMS) detectors simultaneously. AsB was used as a marker to optimize flow through the split-flow valve until maximum signal was achieved in both ICP-MS/MS and ESI-HRMS. After optimization, the polyetheretherketone (PEEK) tubes connecting the valve to ICP-MS/MS and ESI-HRMS were the same size, with the low flow directed to ICP-MS/MS and the high flow to ESI-HRMS (a 30:70 flow split for ICP-MS/MS and ESI-HRMS, respectively). To direct more flow to the ESI-HRMS, we adjusted the ionization source operating conditions to accommodate a flow rate of 500 µL/min, changing the HESI capillary and desolvation temperatures to ensure maximum signal acquisition[1].

## 1.4. Detailed ESI-HRMS acquisition modes and instrument parameters for coupling

Tables S1 and S2 show the instrumental configurations of the ESI source and the Full MS/dd-MS² experiment used for untargeted analysis.

**Table S1**. Heated Electrospray Ionization - HESI Settings for 500 µL/min Feeding Flow.

| **Parameter** | **Value** |
|---|---|
| Sheath Gas Pressure | 60 psi |
| Auxiliary gas flow | 20 au |
| HESI vaporizer temperature | 450 °C |
| Ion transfer tube temperature | 380 °C |
| S-lens RF voltage | 50 au |
| Sweep gas flow | 0 au |
| Spray Voltage | 3.5 kv |
| ESI probe distance | 1.75 mm |
| ESI probe height | Level C |

**Table S2.** Essential Full MS/dd-MS² data-dependent Top 5 acquisition settings.

| **Parameter** | | **Value** |
|---|---|---|
| Full Scan | Resolution | $70 \times 10^3$ |
| $MS^1$ | Polarity | +/- |
| | AGC target | $1 \times 10^6$ |
| | Maximum injection time | 100 ms |
| | Scan range | 70 to 1050 *m/z* |
| $MS^2$ | Resolution | $35 \times 10^3$ |
| | AGC target | $1 \times 10^5$ |

| | |
|---|---|
| Maximum injection time | 50 ms |
| Loop count | 5 |
| Isolation window | 1 *m/z* |
| Collision energy | 30, 40, 50 NCE |
| Minimum AGC target | $1.0 \times 10^3$ |
| Exclude Isotopes | Enabled |
| Dynamic exclusion window | 10 s |

AGC: automatic gain control.

## 1.5. Data processing, analysis, quality control, and metabolite annotation

### 1.5.1. Lag correction between ICP-MS/MS and ESI-HRMS chromatograms

Due to differences in fluidic path length, ionization interface, and acquisition timing, the retention times (RTs) obtained from ICP-MS/MS and ESI-HRMS are not directly comparable[2]. To correct the time offset, we used arsenobetaine (AsB), which was detected in both systems, as an internal time calibrant. We determined a fixed time shift, Δt, defined as the difference in time between the apex of the AsB extracted-ion chromatogram (XIC, m/z 179.0052) in ESI-HRMS and the ^75As trace in ICP–MS/MS. Specifically, Δt ≡ tICP-MS/MS(AsB) – tESI-HRMS(AsB). For subsequent analyses, this Δt value was applied as a global correction (Δt = +22 s for the control sample; Δt = 0 s for the Au NP-treated sample). Additionally, a local micro-lag refinement (±15 s window) was performed for each analyte to ensure optimal overlap between the ESI-HRMS and ICP-MS/MS signals. This assumption of a relatively constant offset during parallel molecular and elemental detection aligns with findings from previous studies in Parallel Elemental and Molecular Mass Spectrometry (PEMMS) and other comparable studies involving LC-ICP–MS and electrospray ionization mass spectrometry (LC–ICP-MS/ESI-HRMS)[3–7].

For the pooled control sample, the ICP-MS/MS AsB signal eluted about 22 s later than the corresponding ESI-HRMS signal; therefore, we applied a +22 s shift to the ESI-HRMS trace. In the pooled Au NP-treated sample, the AsB signals were effectively coincident (Δt ≈ 0 s), and no global time shift was applied. These global corrections were used for the subsequent chromatographic overlays and comparisons. Figure S3 shows the ESI-HRMS AsB XIC and the corresponding $^{75}$As ICP-MS/MS trace for the control and Au NP-treated samples before and after the time-shift correction.

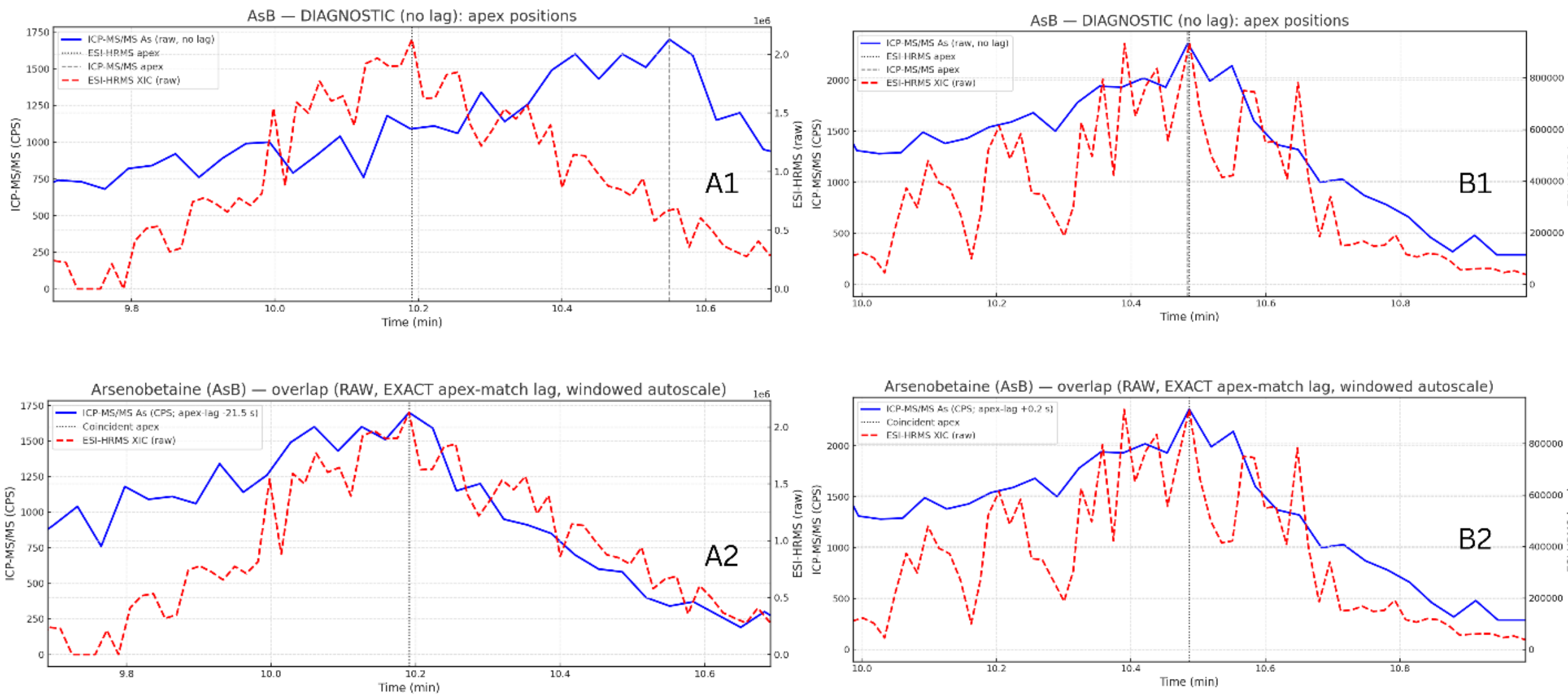


**Figure S3.** Overlays of the arsenobetaine (AsB) extracted-ion chromatogram obtained by ESI-HRMS and the corresponding $^{75}As$ trace obtained by ICP-MS/MS for the pooled control sample (A) and pooled Au NP-treated sample (B), before (A1 and B1) and after (A2 and B2) global time-shift correction. A +22 s shift was applied to the ESI-HRMS trace for the control sample, whereas no global shift was applied to the Au NP-treated sample.

### 1.5.2. Annotation of P- and S-containing molecular features

Preprocessing and conversion of RAW to mzML

The RAW files were converted to mzML using the MSConvert tool from ProteoWizard. This approach applied a peak-picking filter (centroidization and vendor peak picking for $MS^1$ and $MS^2$), standardizing the data and enabling peak detection, ion-current extraction (XIC), and $MS^2$ spectral evaluation.

Data Processing

The mzML files were processed using an in-house Python pipeline (Scripts 1–3), which consists of three main steps:

- Script 1 — $MS^1$ detection and screening

The mzML files were analyzed in Python to detect candidate precursor ions in $MS^1$, incorporating isotopic information, which comprised various compounds and metabolites expected to be present in plants, with checks in metal-aware mode. We used an acceptable error cutoff of ±5 ppm for positive ionization mode and ±10 ppm for negative ionization mode. Simultaneously, the same samples were processed in Thermo Compound Discoverer (CD) with the workflow "Untargeted Metabolomics with Statistics — Detect Unknowns with ID using Online Databases

and mzLogic." This workflow included retention time (RT) alignment, unknown compound detection, gap filling, formula assignment, online database searches, and mzLogic scoring. We filtered the CD output to retain only compounds with annotated formulas containing sulfur (S), phosphorus (P), or both. This filtered set was merged with our internal target list, which included names, formulas, adducts, polarity, and expected $MS^2$ fragments when available. The internal target list comprised compounds and metabolites expected to be present in plants containing S, P, or both in their structure. For each target (both internal and from the CD), we extracted the XIC by summing $MS^1$ intensities within the mode's ppm error window. The integration was vectorized using searchsorted indices and cumulative sums, allowing for a single mzML pass per batch of targets. The apex of the XIC (t_ESI-HRMS) was identified as the local maximum. In the $MS^1$ spectrum at t_ESI-HRMS, the observed m/z of the most intense peak in the same ppm window was noted, the error (ppm) was calculated, and the isotopic check (M, M+1, M+2) was performed, correcting for 1.0033548 Da/z per increment. The isotopic criteria were as follows: for targets containing S, M+2/M must be ≥ 0.04; for other targets, M+1/M must be ≥ 0.01.

- Script 2 — $MS^2$ annotation support

For all candidate features retained after $MS^1$ screening, we evaluated whether an $MS^2$ spectrum generated from the corresponding precursor was available. When $MS^2$ data were available, spectra acquired within ±0.30 min of t_ESI-HRMS were evaluated manually, applying the same precursor mass tolerance and requiring at least one expected or characteristic fragment supporting the proposed annotation within the corresponding mass-error tolerance. The results were documented with annotated PNGs and corresponding tables. Script 3 then evaluated the chromatographic overlap between the candidate features supported by $MS^2$ and the ICP-MS/MS signals monitored for ^32S^16O and ^31P^16O.

- Script 3 — ESI-HRMS × ICP-MS/MS (RT-first correlation and quality control)

Retention times (RTs) from ESI-HRMS and ICP-MS/MS were not directly comparable because the two systems differ in fluidic path lengths, ionization interfaces, and acquisition timing. To correct this temporal offset, arsenobetaine (AsB) was used as an internal reference since it was consistently detected in both datasets. The difference between the AsB XIC apex (m/z 179.0052) and the corresponding ^75As ICP–MS/MS peak was calculated and applied as a fixed shift. For the control profile, the ICP-MS/MS AsB signal eluted about 22 s later than the ESI-HRMS signal; therefore, we applied a +22 s shift to the ESI-HRMS trace. The Au NP-treated profile did not require a global adjustment (Δt ≈ 0 s).

After applying the fixed correction, we further examined each candidate feature and applied a local micro-lag refinement when necessary. The selected micro-lag was recorded for traceability. After alignment, the ESI-HRMS and ICP-MS/MS chromatograms were evaluated using an RT-first approach. For each candidate feature, we recorded the remaining retention-time difference between the two signal apexes (ΔRT), the Pearson correlation between the locally normalized chromatographic profiles, the HRMS peak width, and the normalized ICP-MS/MS peak height. Element-specific overlap was accepted when the apex separation remained within the maximum allowed retention-time window of 0.67 min (approximately 40 s) and the normalized ICP-MS/MS peak height was greater than 0.2. Pearson correlation and HRMS peak width were retained as descriptive alignment metrics and were not used as standalone exclusion thresholds. The source code and computational workflows used in this study have been deposited in Zenodo[8].

Final feature-retention and annotation-support criteria

We considered a candidate feature analytically supported and retained only when it met all required criteria. First, the precursor ion had to fall within the defined mass-accuracy window (±5 ppm in positive mode; ±10 ppm in negative mode) and pass the isotopic check: for sulfur-containing species, the M+2 signal was required to be ≥0.04 relative to the monoisotopic peak; for non-sulfur species, an M+1 contribution of ≥0.01 was required. When $MS^2$ data were available, at least one fragment consistent with the proposed annotation and within the same mass-tolerance window was required. Finally, element-specific support required an ICP-MS/MS peak within the maximum allowed retention-time window of 0.67 min relative to the HRMS apex and a normalized ICP-MS/MS peak height greater than 0.2 after applying the global time shift and, when necessary, local micro-lag refinement. Pearson correlation and HRMS peak width were recorded as descriptive metrics of chromatographic similarity and peak shape.

Quality Control (AsB as internal standard)

To assess script performance, AsB was used as a quality-control and retention-time alignment standard because it was added to both analytical preparations. For Script 1 ($MS^1$), AsB was included in the target list and was required to be detected within ±5 ppm mass error in both the control and Au NP-treated profiles. For Script 2 ($MS^2$), the presence of an AsB $MS^2$ spectrum and the plausibility of its fragmentation within the specified mass-error tolerance were verified. For Script 3 (ESI-HRMS × ICP-MS/MS overlap), the molecular and elemental AsB signals were aligned using the AsB-derived temporal shifts (+22 s for the control profile and 0 s for the Au NP-treated profile) and evaluated using the same co-elution criteria applied to the candidate features.

These AsB checkpoints supported traceability and consistent processing from $MS^1$ feature detection to elemental correlation by ICP-MS/MS. Section 2.2 presents detailed quality-control results.

Blank analysis (contamination and carryover control)

Blank injections were processed using the same pipeline (Scripts 1–3) and parameters applied to the biological profiles, including mass-error tolerances, retention-time windows, ICP-MS/MS quality-control criteria, and the AsB-based temporal correction when applicable. For each candidate feature retained from either profile, the blank was examined for an $MS^1$ signal within the corresponding mass-error window (±5 ppm in positive mode or ±10 ppm in negative mode) and retention-time window (±0.30 min). When $MS^2$ spectra were available in the blank, their consistency with the proposed annotation was also evaluated within the same retention-time and mass-error tolerances. Element-specific co-elution for P, S, or both elements was assessed using the same Script 3 criteria applied to the biological profiles. A candidate feature was retained only when the blank did not meet the combined molecular and element-specific criteria at the corresponding retention time. Features meeting these criteria in the blank were classified as potential background or carryover and excluded from the final retained list; these excluded features are reported in Table S8. This procedure reduced the likelihood that the retained features originated from analytical background or carryover. The blank-processing script is included in the publicly available computational workflow cited above.

2. RESULTS

2.1. Anatomical Analysis of soybean callus

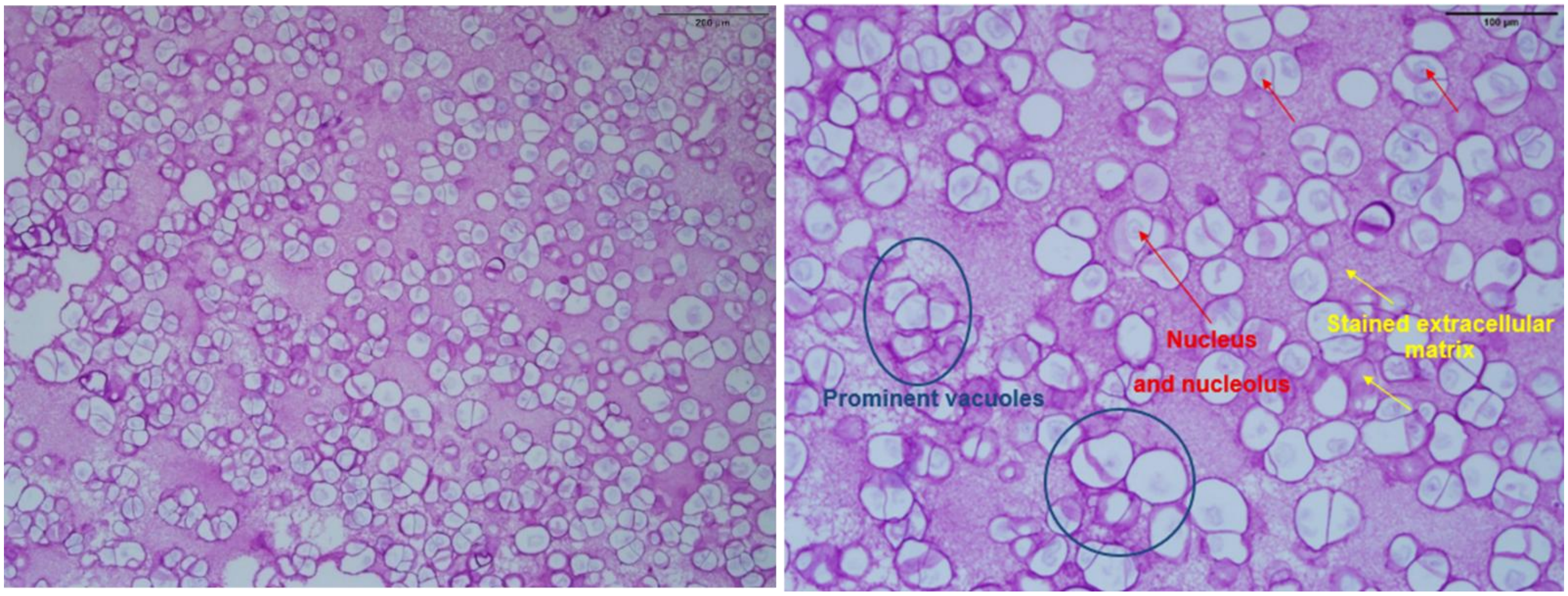


**Figure S4.** Anatomical analysis of soybean callus. The images show a majority of large, prominent vacuoles (blue circles), the nucleus and nucleolus (red arrows), and the stained extracellular matrix (yellow arrows).

2.2. Quality control

**Table S3**. Results of arsenobetaine (AsB) in Script 1.

| mzml | Found | any_ms1_ok | best_mass_error_ppm | best_iso_similarity | rt_apex_min | QC_MS1_STATUS |
|---|---|---|---|---|---|---|
| AU NP_POS.mzML | TRUE | TRUE | 1.5799175 | 0.99972047 | 10.486735 | PASS |
| CONTROL_POS.mzML | TRUE | TRUE | 2.43234191 | 0.998427 | 10.190992 | PASS |

**Table S4**. Results of AsB in Script 2.

| mzml | polarity | rt_apex_min | asb_mz | rt_ms2 | ppm_precursor | status | used_window_min | source | match_selected |
|---|---|---|---|---|---|---|---|---|---|
| AU NP_POS.mzML | POS | 10.486735 | 179.0046 | 10.415336 | 1.92088726 | PASS | 1.5 | selected | 7 |
| CONTROL_POS.mzML | POS | 10.190992 | 179.0046 | 9.912974 | 1.92088726 | PASS | 1.5 | selected | 4 |

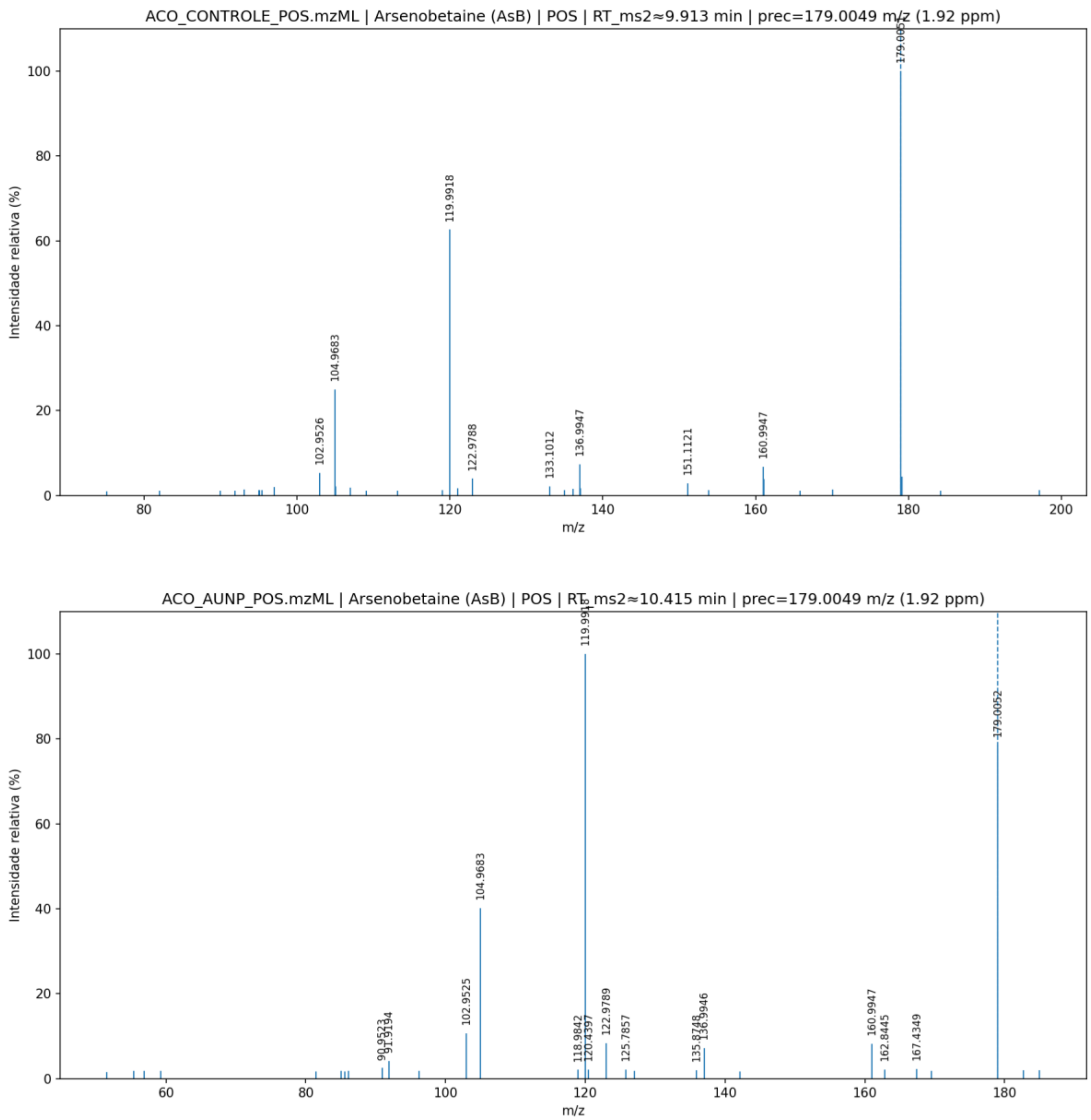


**Figure S5**. $MS^2$ of AsB in the control sample and treated with Au NPs, with the precursor and the key fragments.

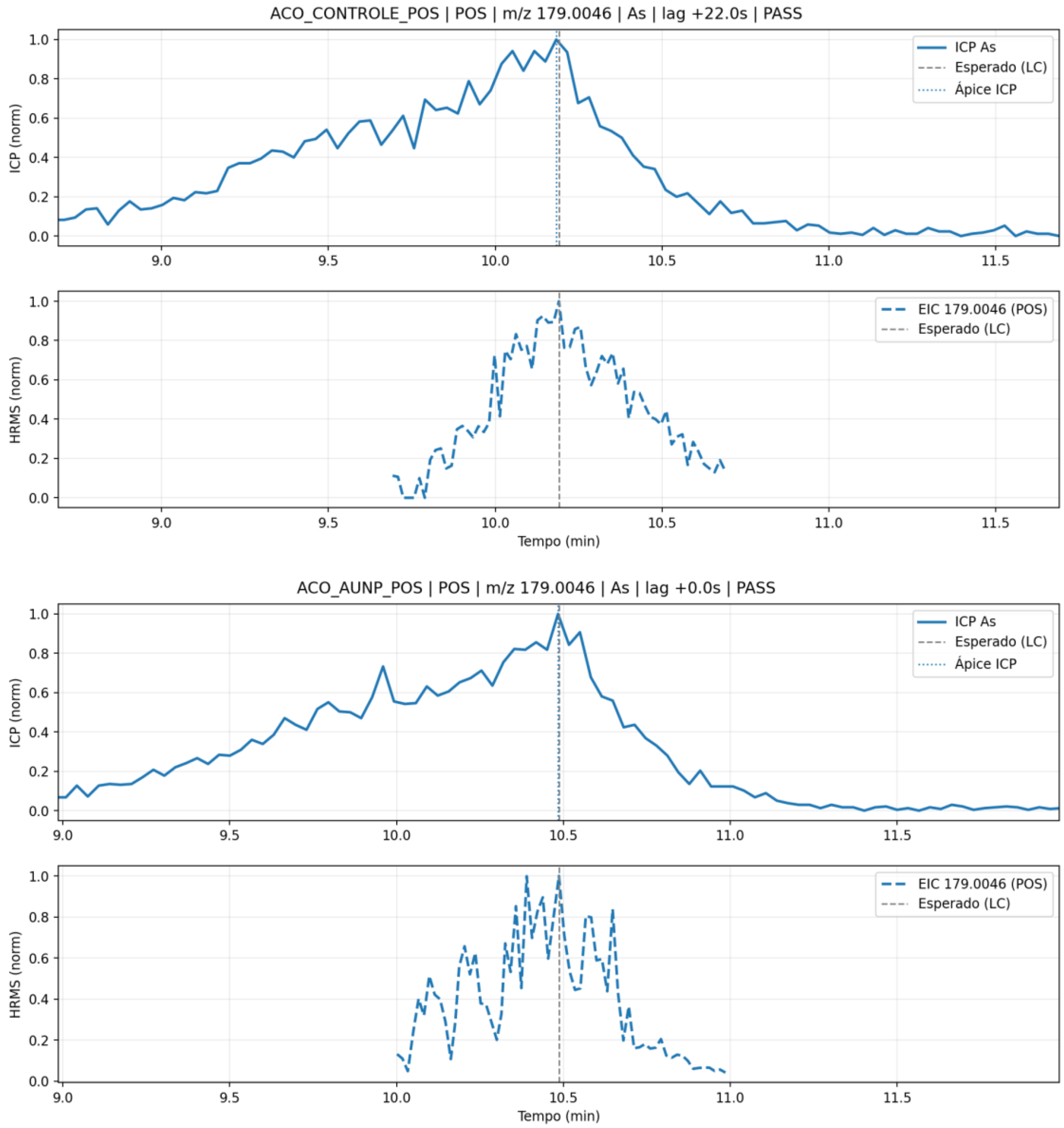


**Figure S6**. Overlap of total As by ICP-MS/MS and AsB signal by ESI-HRMS (extracted ion chromatogram – XIC, m/z 179.0046) for the control sample and the sample treated with Au NP. For the control profile, the ICP-MS/MS AsB signal eluted approximately 22 s later than the ESI-HRMS signal; therefore, a +22 s shift was applied to the ESI-HRMS trace.

## 2.3. P and S-binding metabolites in soybean callus and soybean callus treated with Au NPs

**Table S5**. $MS^1$ results: P- and/or S-containing compounds identified by HILIC-HPLC, ICP-MS/MS, and ESI-HRMS, including retention-time apex, theoretical/observed m/z, mass error, and peak area (normalized by AsB – integrated chromatographic peak area proportional to the detected signal intensity) in soybean callus samples from the control group and those treated with 100 µg $L^{-1}$ Au NPs.

| Sample | Putative metabolite annotations | Formula (neutral) | Adduct | Polarity | RT_apex_MS1_min | Theoretical_mz | Observed_mz_MS1 | Mass_error_ppm | Area_peak |
|---|---|---|---|---|---|---|---|---|---|
| Control | Cys-Pro | C8H14N2O3S | [M-H]- | NEG | 1.22 | 217.065 | 217.066 | 2.08 | 621211 |
| Control | C8H17O9P (putative phosphoglycerol-like metabolite) | C8H17O9P | [M+H]+ | POS | 1.39 | 289.068 | 289.069 | 0.001 | 222390 |
| Control | Phosphorylcholine | C5H14NO4P | [M+H]+ | POS | 29.22 | 184.073 | 184.074 | 1.47 | 765530 |
| Control | [2-(6-aminopurin-9-yl)-1-hydroxy-ethyl]phosphonic acid | C7H10N5O4P | [M+H]+ | POS | 5.35 | 260.054 | 260.054 | -2.69 | 215656 |
| Control | C45H71OP3 | C45H71OP3 | [M+H]+ | POS | 0.70 | 721.479 | 721.481 | 2.39 | 24397 |
| Control | Choline O-Sulfate | C5H13NO4S | [M+H]+ | POS | 2.07 | 184.064 | 184.064 | 2.11 | 203926 |
| Control | Cys-Pro | C8H14N2O3S | [M+H]+ | POS | 1.26 | 219.080 | 219.080 | 0.69 | 8143114 |
| Control | L-homomethionine | C6H13NO2S | [M+H]+ | POS | 36.77 | 164.074 | 164.074 | 0.46 | 2311909 |
| Control | LPC(16:0) - 1-palmitoyl-2-hydroxy-sn-glycero-3-phosphocholine | C24H50NO7P | [M+H]+ | POS | 0.96 | 496.340 | 496.340 | 1.20 | 87746 |
| Control | 5-deoxy-5-methylthioadenosine (MTA) | C11H15N5O3S | [M+H]+ | POS | 1.17 | 298.097 | 298.097 | 0.80 | 292309 |
| Control | Thiamine | C12H16N4OS | [M+H]+ | POS | 16.45 | 265.112 | 265.112 | 0.91 | 1153307 |
| Control | AMP | C10H14N5O7P | [M-H]- | NEG | 15.14 | 346.056 | 346.056 | 1.79 | 149533 |
| Control | Glycerol-3-phosphate (G3P) | C3H9O6P | [M-H]- | NEG | 15.19 | 171.006 | 171.007 | 4.52 | 677829 |
| Control | UMP | C9H13N2O9P | [M-H]- | NEG | 15.45 | 323.029 | 323.029 | 1.34 | 510635 |
| Control | Methionine | C5H11NO2S | [M+H]+ | POS | 6.80 | 150.058 | 150.059 | 2.70 | 7593308 |
| Au NPs | Cys-Pro | C8H14N2O3S | [M-H]- | NEG | 1.25 | 217.065 | 217.066 | 2.64 | 364920 |
| Au NPs | Cys-pPro | C8H14N2O3S | [M+H]+ | POS | 1.23 | 219.080 | 219.080 | 1.52 | 347586 |
| Au NPs | Cysteine sulfinic acid | C3H7NO4S | [M+Cl]- | NEG | 14.75 | 152.002 | 152.003 | 4.43 | 148425 |
| Au NPs | Cystine | C6H12N2O4S2 | [M-H]- | NEG | 24.57 | 239.017 | 239.017 | 1.01 | 113974 |
| Au NPs | L-homomethionine | C6H13NO2S | [M+H]+ | POS | 36.08 | 164.074 | 164.074 | 1.29 | 134381 |
| Au NPs | Methionine | C5H11NO2S | [M+H]+ | POS | 7.25 | 150.058 | 150.059 | 2.60 | 206217 |
| Au NPs | Methionine sulfoxide | C5H11NO3S | [M+H-H2O]+ | POS | 2.07 | 148.043 | 148.043 | 0.64 | 162780 |
| Au NPs | Sulfolactate | C3H6O6S | [M-H]- | NEG | 14.23 | 168.981 | 168.982 | 4.24 | 634198 |
| Au NPs | Phosphocholine - DHA lipid | C44H76NO8P | [M+H]+ | POS | 1.20 | 778.538 | 778.540 | 1.98 | 14894 |
| Au NPs | Hexose phosphate isomers | C6H13O9P | [M-H]- | NEG | 19.78 | 259.022 | 259.022 | -0.40 | 5573041 |

| | | | | | | | | | |
|---|---|---|---|---|---|---|---|---|---|
| Au NPs | 4-Methyl-5-thiazoleethanol | C6H9NOS | [M+H]+ | POS | 16.35 | 144.048 | 144.048 | 1.90 | 988245 |
| Au NPs | C23H51N3O4P2 - Phosphocholine lipid (PC) | C23H51N3O4P2 | [M+Na]+ | POS | 0.89 | 518.325 | 518.325 | 0.96 | 40839 |
| Au NPs | C27H23N2O5PS | C27H23N2O5PS | [M+H]+ | POS | 0.96 | 519.114 | 519.114 | 0.28 | 41533 |
| Au NPs | Phosphorylcholine | C5H14NO4P | [M+H]+ | POS | 29.43 | 184.073 | 184.074 | 1.97 | 10330 |
| Au NPs | LPC (16:0) - 1-palmitoyl-2-hydroxy-sn-glycero-3-phosphocholine | C24H50NO7P | [M+H]+ | POS | 1.10 | 496.340 | 496.341 | 1.94 | 309453 |
| Au NPs | UMP | C9H13N2O9P | [M-H]- | NEG | 15.45 | 323.029 | 323.030 | 3.48 | 309453 |
| Au NPs | Thiamine | C12H16N4OS | [M+H]+ | POS | 16.56 | 265.112 | 265.112 | 0.57 | 5035169 |
| Au NPs | Cysteine sulfonic acid | C3H7NO5S | [M-H]- | NEG | 8.41 | 167.997 | 167.998 | 4.94 | 4052256 |
| Au NPs | UDP-hexose | C15H24N2O17P2 | [M-H]- | NEG | 19.25 | 565.048 | 565.050 | 4.40 | 70892 |
| Au NPs | UDP-N-acetylglucosamine | C17H27N3O17P2 | [M-H]- | NEG | 18.30 | 606.074 | 606.075 | 0.98 | 59699 |
| Au NPs | S-methylmethionine (SMM) | C6H13NO2S | [M+H]+ | POS | 36.08 | 164.074 | 164.074 | 1.30 | 134381 |
| Au NPs | Glycerophosphocholine (GPC) | C8H20NO6P | [M+H]+ | POS | 18.93 | 258.110 | 258.109 | -2.93 | 53089 |
| Au NPs | Glycerol-3-phosphate (G3P) | C3H9O6P | [M-H]- | NEG | 15.18 | 171.006 | 171.007 | 4.70 | 333623 |

*POS = positive; NEG = negative; RT_apex = retention-time apex.*

**Table S6.** MS² results: P- and/or S-containing compounds identified by HILIC–HRMS/ICP–MS/MS, including the MS² retention-time apex, theoretical/observed m/z, mass error, key fragments supporting the molecular structure with their m/z values, fragment mass errors, and the compound classes identified in soybean callus samples from the control group and those treated with 100 μg $L^{-1}$ Au NPs.

| Sample | Putative metabolite annotations | Polarity | RT_MS2_min | Observed_mz_MS2 | Mass_error_ppm_MS2 | Key_ fragments | Fragment_ ppm_errors | Class |
|---|---|---|---|---|---|---|---|---|
| Control | Cys-Pro | NEG | 1.37 | 217.066 | 1.668 | H2S @ 183.0800 | 3.82 | Peptide/dipeptide |
| Control | C8H17O9P (putative phosphoglycerol-like metabolite) | POS | 1.35 | 289.0684 | 0.212 | C7H7 @ 91.0548; C6H9O @ 97.0653; C8H5O3 @ 149.0239; C11H4O4 @ 200.0110 | -3.47; -2.53; -4.23; -0.62 | Lysophosphatidylglycerol (LPG, lipid) |
| Control | Phosphorylcholine | POS | 28.93 | 184.074 | 0.967 | H4O4P1+ @ 98.9844; C2H1O4P1+ @ 119.9610; C2H6O4P1+ @ 125.0001 | -3.65; -2.12; -2.29 | Organophosphate (putative) |
| Control | [2-(6-aminopurin-9-yl)-1-hydroxy-ethyl]phosphonic acid | POS | 5.15 | 260.055 | 4.299 | C3H2N5O3P @ 186.9889 | -3.31 | Acyclic nucleoside phosphonate (synthetic / artifact) |
| Control | C45H71OP3 | POS | 0.71 | 721.481 | 2.350 | H3PO4+H+ @ 98.9845 | -2.11 | Phospholipid-related (putative PC/DAG intermediate) |
| Control | Choline O-Sulfate | POS | 2.05 | 184.064 | 1.597 | C5H12N+ @ 86.0969; C3H10N+ @ 60.0812 | +5.00; +14.00; +0.00; +5.00; +12.00; -1.44; +3.00; +10.00; -1.84 | Quaternary ammonium sulfate (osmolyte) |
| Control | Cys-Pro | POS | 1.11 | 219.080 | 0.502 | C2H6N5+ @ 76.0218;C5H10NO2+ @ 116.0709; [M + H - H2O] @ 201.0695 | -3.89;-2.18;+0.82 | Peptide/dipeptide |
| Control | L-homomethionine | POS | 36.87 | 164.074 | 0.146 | C3H6N+ @ 56.0497; C3H8NO+ @ 74.0602; C4H12NS+ @ 102.0552 | -5.66; +2.65; +2.46 | Amino acid |
| Control | LPC(16:0) - 1-palmitoyl-2-hydroxy-sn-glycero-3-phosphocholine | POS | 1.00 | 496.340 | 1.200 | C5H14NO+ @ 104.1073; C5H15NO4P+ @ 184.0738; [M+H-H2O]+ @ 478.3308 | +2.51; +2.75; +2.05 | Lysophosphatidylcholine (LPC) |

| | | | | | | | | |
|---|---|---|---|---|---|---|---|---|
| Control | 5-deoxy-5-methylthioadenosine (MTA) | POS | 1.18 | 298.097 | 0.803 | C4H7NO2+ @ 75.0264; C5H6NO+ @ 94.0401; C4H5O3+ @ 97.0285; C4H7N2O+ @ 103.0214; C5H11NO2+ @ 119.0353; C5H6N5+ (adenine) @ 136.0619; C7H9NO+ @ 145.0320; C6H7N2O+ @ 163.0426 | +1.29; +0.89; +1.69; +0.67; +1.29; +1.38; -0.23; -0.08 | Organosulfur amine (putative) |
| Control | Thiamine | POS | 16.29 | 265.112 | 0.536 | C6H8N3+ @ 122.0715; C5H10NOS+ @ 144.0480 | +2.54; +2.91 | Vitamin/cofactor (thiamine) |
| Control | AMP | NEG | 15.12 | 346.056 | 1.680 | PO3- @ 78.9593; C4H4N2O2-@ 96.9701; C5H8NO4- @ 128.0358; C5H8NO5- @ 134.0479;C5H9N2O3-@150.9806 | 10.10;5.16;3.91;4.48;2.65 | Nucleotide (adenylate) |
| Control | Glycerol-3-phosphate (G3P) | NEG | 15.17 | 171.007 | 4.253 | PO3- @ 78.9593 | 9.9 | Sugar phosphate / glycerophosphate |
| Control | UMP | NEG | 15.52 | 323.029 | 1.000 | C4H3N2O2- @ 111.0206 | 8.24 | Nucleotide (uridylate) |
| Control | Methionine | POS | 7.38 | 150.058 | 0.999 | C4H10NS+ @ 104.0530 | 0.31 | Amino acid |
| Au NPs | Cys-Pro | NEG | 1.26 | 217.066 | 2.851 | H2O+COS @ 99.0664; CS+H2 @ 137.0979; H2S @ 173.0190 | 5.0; +4.6; +5.2 | Peptide/dipeptide |
| Au NPs | Cys-Pro | POS | 1.68 | 219.080 | 0.959 | C5H10NO2+ @ 116.0706; H2O @ 201.1040 | 2.5; +4.2 | Peptide/dipeptide |
| Au NPs | Cysteine sulfinic acid | NEG | 14.71 | 152.003 | 4.526 | SO2- @ 63.9625 | 4.28 | Sulfur amino acid derivative (oxidized) |
| Au NPs | Cystine | NEG | 24.45 | 239.017 | 0.758 | C3H6NO2S- @ 120.0131 | 5.28 | Organosulfur amine (putative) |

| | | | | | | | | |
|---|---|---|---|---|---|---|---|---|
| Au NPs | L-homomethionine | POS | 35.94 | 164.074 | 1.365 | CO+C2H6S @ 74.0069; H2O+C2H6S @ 75.0227; C2H6S @ 62.0228 | +3.1; +2.6; +3.4 | Amino acid |
| Au NPs | Methionine | POS | 7.99 | 150.059 | 0.973 | H2O+CS @ 88.0544; CS @ 100.0039; H2O+CO @ 101.0186; CH3SH @ 103.0524; NH3 @ 133.0842 | 2.2; +1.5; +3.1; +2.9; +3.4 | Amino acid |
| Au NPs | Methionine sulfoxide | POS | 2.24 | 148.043 | 1.158 | CH3SOH @ 85.0109; H2O+CO @ 102.0497; NH3 @ 131.0811 | 2.3; +3.7; +2.6 | Amino acid |
| Au NPs | Sulfolactate | NEG | 14.18 | 168.982 | 4.327 | H2O+SO3 @ 65.0134; HSO3- @ 80.9647; SO3- @ 79.9576; H2O+SO2 @ 82.9404; HSO4- @ 96.9604; H2O+CO2 @ 100.9861; H2O @ 148.9713 | +1.6; +10.4; +9.7; +4.2; +11.0; +7.2; +4.8 | Sulfate/sulfonate (putative) |
| Au NPs | Phosphocholine - DHA lipid | POS | 1.21 | 778.539 | 1.352 | C5H12N+ (choline) @ 86.0967; phosphocholine-NMe3 (NL 59) @ 166.0989; phosphocholine @ 184.0733 | -3.7; +2.6; +3.2 | Phosphocholine headgroup |
| Au NPs | Hexose phosphate isomers | NEG | 20.62 | 259.022 | 0.019 | PO3- (C0H0O3P1) @ 78.9584; H2PO4- (C0H2O4P1) @ 96.9692; C2H4O5P- (sugar-phosphate fragment) @ 136.9829 | 10.6; +11.2; +8.2 | Sugar phosphate (hexose phosphate) |

| | | | | | | | | |
|---|---|---|---|---|---|---|---|---|
| Au NPs | 4-Methyl-5-thiazoleethanol | POS | 17.10 | 144.048 | 1.659 | C2H4O+CS @ 63.4746; CH2O+CS @ 68.0497; CO+NH3 @ 73.0107; CS+H2 @ 95.5758; CH2O+H2 @ 113.0297; H2O @ 126.6571 | +4.7; +2.7; +3.6; +3.1; +3.1; +3.3 | Organosulfur amine (putative) |
| Au NPs | C23H51N3O4P2 - Phosphocholine lipid (PC) | POS | 0.90 | 518.325 | 0.958 | C5H12N+ @ 86.0967; C5H14NO+ @ 104.1073; C5H15NO4P+ @ 184.0738; C2H8NO4P+ @ 125.0002; [M+Na-H2O]+ @ 500.3146 | -3.7; +2.9; +2.8; +1.5; +2.7 | Phosphocholine headgroup |
| Au NPs | C27H23N2O5PS | POS | 1.09 | 519.114 | 0.868 | C5H12N+ (choline-type) @ 86.0966; C7H7N+ (benzylamine-type) @ 91.9213; C8H11NO2+ @ 153.0385; C13H12NO3+ @ 215.0705; C14H11NO4+ @ 243.0658; C16H15NO4+ @ 271.0609 | | Unknown phospho-sulfur aromatic compound |
| Au NPs | Phosphorylcholine | POS | 29.95 | 184.074 | 1.473 | Phosphocholine cation (184.0739); C2H6O4P+ (124.9999); C5H12N+ (86.0967) | +3.05; -3.57; -3.22 | Organophosphate (putative) |
| Au NPs | LPC(16:0) - 1-palmitoyl-2-hydroxy-sn-glycero-3-phosphocholine | POS | 1.10 | 496.340 | 1.326 | C5H14NO+ (choline) @ 104.10699; C5H12N+ @ 86.09697; C2H5PO4+ @ 125.0000; C5H15NO4P+ (phosphocholine) @ 184.0738 | +1.29; +0.89; +1.69; +0.67; +1.29; +1.38; -0.23; -0.08 | Lysophosphatidylcholine (LPC) |

| | | | | | | | | |
|---|---|---|---|---|---|---|---|---|
| Au NPs | UMP | NEG | 16.19 | 323.029 | 1.242 | H2PO4- (96.9696) \| 96.9701 m/z | +5.16 | Nucleotide (uridylate) |
| Au NPs | Thiamine | POS | 16.73 | 265.112 | 0.911 | C3H4NS+ (thiazolium) @ 122.0345; C4H4N2+ (pyrimidine) @ 144.0449 | +2.6; +3.7 | Vitamin/cofactor (thiamine) |
| Au NPs | Cysteine sulfonic acid | NEG | 8.48 | 167.998 | 4.665 | C3H3O2- @ 71.0140; HSO3- @ 80.9655; C3H4NO2- @ 86.0243; [M–H–NH3- @ 150.9715 | +10.1; +10.5; +11.4; +9.3 | Organosulfur amine (putative) |
| Au NPs | UDP-hexose | NEG | 19.56 | 565.050 | 4.665 | PO3- @ 78.9593; H2PO4- @ 96.9702; UMP- @ 323.0299; UDP?H2O @ 386.0096 | +10.3; +11.7; +0.5; -9.1 | Nucleotide sugar |
| Au NPs | UDP-N-acetylglucosamine | NEG | 18.48 | 606.075 | 1.186 | PO4H- @ 96.9700 | +4.50 | Nucleotide (uridylate) |
| Au NPs | S-methylmethionine (SMM) | POS | 36.47 | 164.074 | 1.371 | C4H8NO2+ @ 102.0553; C3H6N+ @ 56.0497; C3H8NO+ @ 74.0603 | +4.00; +3.00; +3.00 | Amino acid derivative (sulfonium) |
| Au NPs | Glycerophosphocholine (GPC) | POS | 19.22 | 258.110 | 0.331 | C5H14NO+ @ 104.1073; C5H15NO4P+ @ 184.0740 | -2.01; +0.82 | Phosphocholine headgroup |
| Au NPs | Glycerol-3-phosphate (G3P) | NEG | 15.77 | 171.007 | 3.896 | PO3- @ 78.9593; H2PO4- @ 96.9701 | +3.00; +2.00 | Sugar phosphate/glycerophosphate |

**Table S7**. Overlap parameters between molecular (ESI-HRMS) and elemental (ICP-MS/MS) signals for P- and S-containing candidate features retained after MS¹ screening and MS² annotation support.

| Sample | Compound | Element | lag_ selected_s | ICP_apex_ min | HRMS_apex_min | ΔRT_min | Pearson_r | FWHM_HRMS_min | Overlap |
|---|---|---|---|---|---|---|---|---|---|
| Control | Cys-pro (NEG) | S | −22.00 | 0.58 | 1.21 | −0.63 | −0.11 | 0.15 | PASS |
| Control | C8H17O9P (putative phosphoglycerol-like metabolite) | P | −0.30 | 15.7 | 15.45 | 0.25 | 0.68 | 0.3 | PASS |
| Control | Phosphorylcholine | P | 1.95 | 29.53 | 29.22 | 0.32 | 0.78 | 0.08 | PASS |
| Control | [2-(6-aminopurin-9-yl)-1-hydroxy-ethyl]phosphonic acid | P | -3.50 | 5.12 | 4.75 | 0.37 | 0.17 | 0.02 | PASS |
| Control | C45H71OP3 | P | -5.76 | 0.50 | 0.70 | -0.20 | 0.15 | 0.04 | PASS |
| Control | Choline O-Sulfate | S | 15.00 | 1.86 | 2.07 | -0.21 | 0.03 | 0.02 | PASS |
| Control | Cys-pro (POS) | S | −46.00 | 0.24 | 1.31 | −1.07 | 0.01 | 0.15 | PASS |
| Control | L-homomethionine | S | 10.50 | 36.79 | 36.77 | 0.02 | 0.11 | 0.01 | PASS |
| Control | LPC:16 - 1-palmitoyl-2-hydroxy-sn-glycero-3-phosphocholine | P | 8.00 | 0.73 | 0.96 | -0.23 | 0.03 | 0.08 | PASS |
| Control | 5-deoxy-5-methylthioadenosina (MTA) | S | 12.39 | 0.80 | 1.17 | -0.37 | 0.28 | 0.07 | PASS |
| Control | Thiamine | S | 2.00 | 17.05 | 16.45 | 0.60 | 0.40 | 0.12 | PASS |
| Control | AMP | P | −24.90 | 15.63 | 15.12 | 0.51 | 0.55 | 0.29 | PASS |
| Control | Glycerol-3-phosphate (G3P) | P | -29.90 | 15.20 | 15.19 | 0.01 | 0.94 | 0.48 | PASS |
| Control | UMP | P | -17.10 | 15.42 | 15.45 | -0.03 | 0.92 | 0.30 | PASS |
| Control | Methionine | S | -22.00 | 7.34 | 6.80 | 0.54 | 0.10 | 0.76 | PASS |
| Au NPs | Cys-pro (NEG) | S | 0 | 0.98 | 1.27 | −0.29 | 0.59 | 0.15 | PASS |
| Au NPs | Cys-pro (POS) | S | 0 | 0.97 | 1.24 | −0.27 | 0.57 | 0.15 | PASS |
| Au NPs | Cysteine sulfinic acid | S | 0.00 | 14.89 | 14.75 | 0.15 | 0.39 | 0.22 | PASS |
| Au NPs | Cystine | S | 0.00 | 24.69 | 24.57 | 0.12 | 0.47 | 0.69 | PASS |
| Au NPs | L-homomethionine | S | 11.00 | 36.02 | 36.08 | -0.07 | 0.16 | 0.09 | PASS |
| Au NPs | Methionine | S | 0.00 | 35.83 | 36.08 | -0.25 | 0.23 | 0.35 | PASS |
| Au NPs | Methionine sulfoxide | S | 0.00 | 2.14 | 2.07 | 0.07 | -0.09 | 0.30 | PASS |
| Au NPs | Sulfolactate | S | 0.00 | 14.89 | 14.23 | 0.66 | 0.41 | 0.50 | PASS |
| Au NPs | Phosphocholine - DHA lipid | P | 11.00 | 1.14 | 1.20 | -0.06 | 0.70 | 0.16 | PASS |
| Au NPs | Hexose phosphate isomers | P | 0.00 | 19.61 | 19.76 | -0.15 | 0.69 | 0.38 | PASS |
| Au NPs | 4-Methyl-5-thiazoleethanol | S | 7.00 | 17.01 | 16.35 | 0.65 | 0.30 | 0.80 | PASS |
| Au NPs | C23H51N3O4P2 - Phosphocholine lipid (PC) | P | -4.50 | 0.88 | 0.89 | -0.01 | 0.64 | 0.00 | PASS |
| Au NPs | C27H23N2O5PS | P | 3.00 | 1.01 | 0.89 | 0.12 | 0.73 | 0.33 | PASS |
| Au NPs | C27H23N2O5PS | S | 3.00 | 1.01 | 0.89 | 0.12 | 0.60 | 0.33 | PASS |
| Au NPs | Phosphorylcholine | P | 0 | 29.31 | 29.37 | -0.06 | 0.75 | 0.14 | PASS |
| Au NPs | LPC:16 - 1-palmitoyl-2-hydroxy-sn-glycero-3-phosphocholine | P | 0.00 | 0.940 | 0.87 | 0.07 | 0.70 | 0.08 | PASS |
| Au NPs | UMP | P | -31.00 | 15.52 | 15.47 | 0.05 | 0.88 | 0.05 | PASS |
| Au NPs | Thiamine | S | 0.00 | 17.09 | 16.56 | 0.52 | 0.28 | 16.56 | PASS |
| Au NPs | Cysteine sulfonic acid | S | -10.50 | 8.32 | 8.41 | -0.09 | 0.23 | 0.06 | PASS |
| Au NPs | UDP-hexose | P | -21.00 | 19.26 | 19.25 | 0.01 | 0.73 | 0.37 | PASS |
| Au NPs | UDP-N-acetylglucosamine | P | -22.50 | 18.32 | 18.30 | 0.02 | 0.49 | 0.08 | PASS |
| Au NPs | S-methylmethionine (SMM) | S | 12.00 | 36.03 | 36.08 | -0.05 | 0.25 | 0.85 | PASS |
| Au NPs | Glycerophosphocholine (GPC) | P | 15.00 | 18.94 | 18.93 | 0.01 | 0.07 | 0.37 | PASS |
| Au NPs | Glycerol-3-phosphate (G3P) | P | -8.00 | 15.84 | 15.18 | 0.66 | 0.53 | 0.20 | PASS |

Lag_selected_s: time shift applied to align HRMS and ICP–MS/MS (positive = ICP elutes later); ICP_apex_min: retention time of the ICP–MS/MS elemental signal; HRMS_apex_min: retention time of the precursor ion in HRMS; ΔRT_min: difference (minutes) between HRMS and ICP–MS/MS apexes (HRMS_apex − ICP_apex); values close to zero indicate good co-elution; Pearson_r: linear correlation coefficient between locally normalized HRMS and ICP–MS/MS chromatographic profiles (evaluated in a ±0.5 min window centered on the HRMS apex); FWHM_HRMS_min: full width at half maximum (minutes) of the HRMS chromatographic peak; Overlap: final element-specific overlap status

recorded after global time-shift correction and local micro-lag refinement. PASS required an apex separation within the maximum allowed retention-time window of 0.67 min and a normalized ICP-MS/MS peak height greater than 0.2. Pearson_r and FWHM_HRMS_min are reported as descriptive metrics and were not used as standalone PASS/FAIL thresholds.

**Table S8**. P- and/or S-containing features detected in the procedural blank, with MS² annotation support, and excluded from the final retained list.

| Compound | polarity | Mz | rt_min | area | MS2_found | precursor_dppm |
|---|---|---|---|---|---|---|
| 2-(2-benzothiazolylthio)ethanol | POS | 212.019832 | 0.96852023 | 349922.5421 | TRUE | 1.96402894 |
| 2-Methylpropane-1-sulfonamide | POS | 138.058326 | 46.555388 | 12307774.47 | TRUE | 14211.707 |
| Benzothiazole | POS | 136.021546 | 2.3468542 | 5365199.801 | TRUE | 2.57578534 |

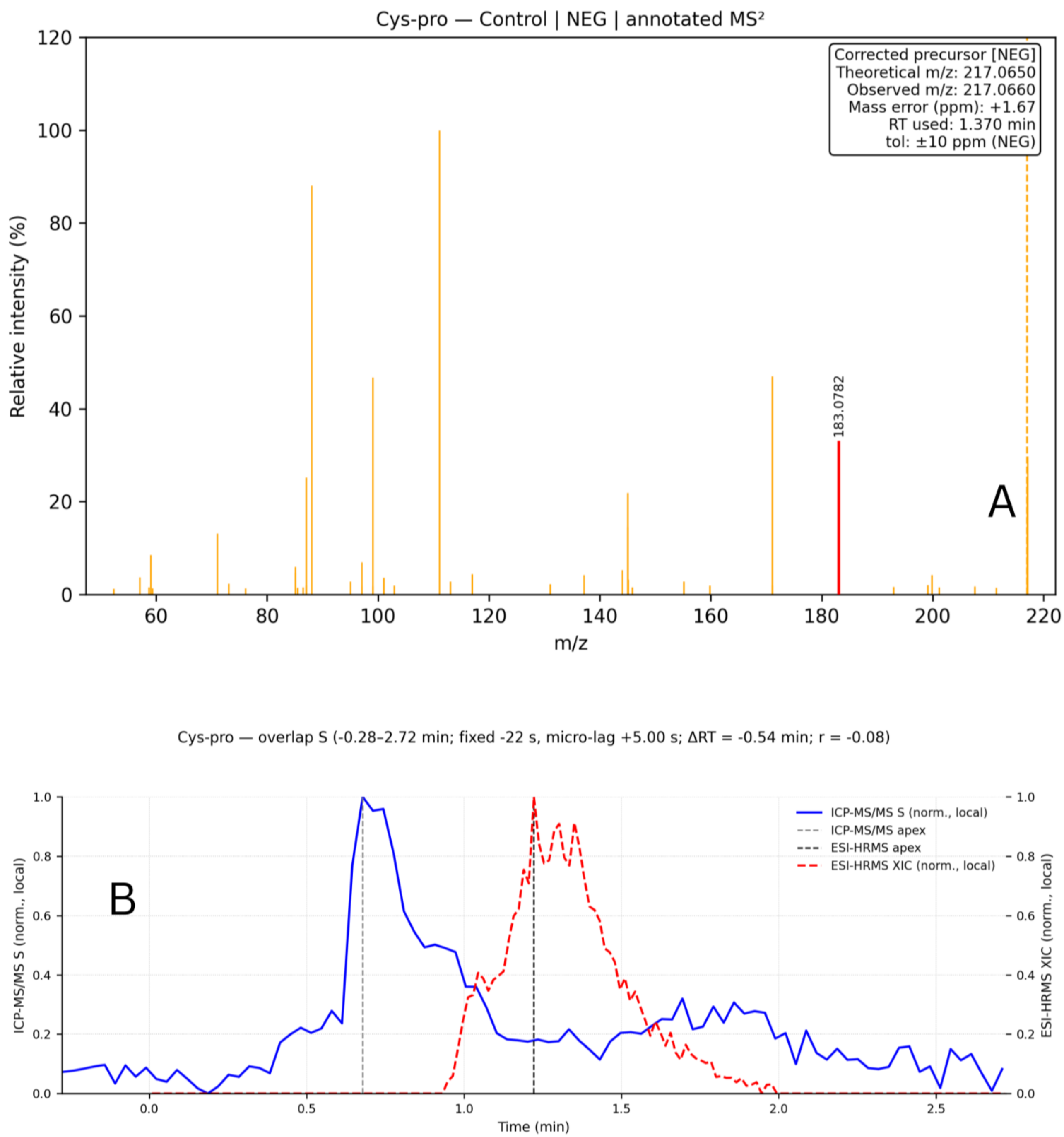


**Figure S7. Cysteine–proline (Cys-Pro) detected in the control callus in negative ion mode.** (A) The MS² confirms the deprotonated precursor at *m/z* 217.0660 (theoretical 217.0650; +2.1 ppm; RT 1.22 min), showing fragment ions consistent with a cysteine–proline dipeptide, including cleavages of the peptide bond and thiol-associated neutral losses. (B) The S-specific ICP-MS/MS signal co-elutes with the HRMS XIC, with a narrow ΔRT window, providing orthogonal elemental confirmation. Cys-Pro is also detected in Au NP-treated callus, indicating a conserved thiol-containing dipeptide pool.

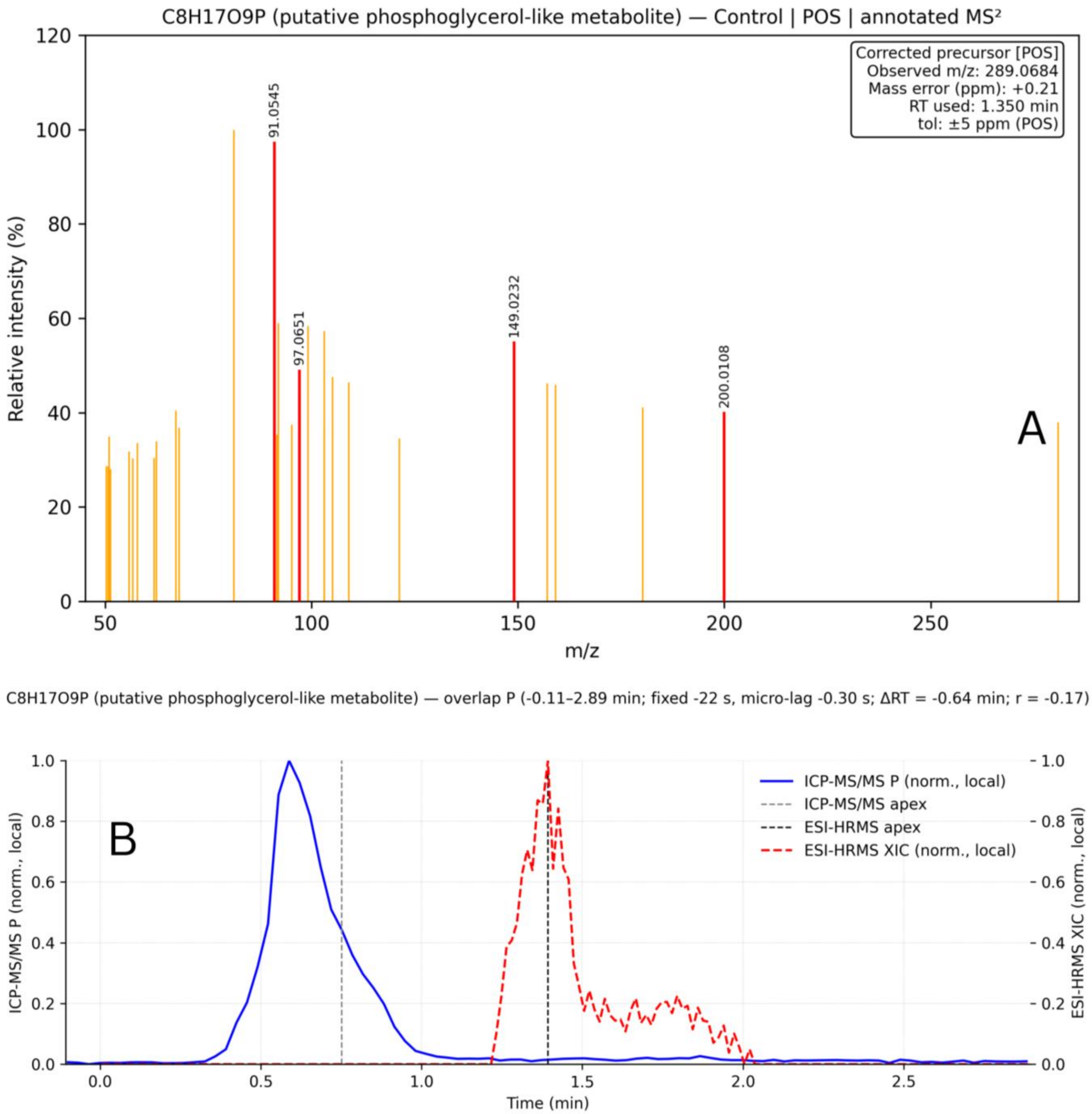


**Figure S8. C8H17O9P (putative phosphoglycerol-like metabolite) detected in control callus in positive ion mode.** (A) The protonated precursor at *m/z* 289.0690 (theoretical 289.0680; ~0 ppm; RT 1.39 min) shows MS² fragments typical of a small phosphoglycerol-like molecule, including neutral losses related to phosphate and glyceryl fragments. (B) Co-elution of the P ICP-MS/MS signal with the HRMS XIC confirms the presence of a P-containing metabolite. This feature was not observed in Au NP-treated callus, suggesting that this phosphoglycerol-like species is depleted or diverted under NP exposure.

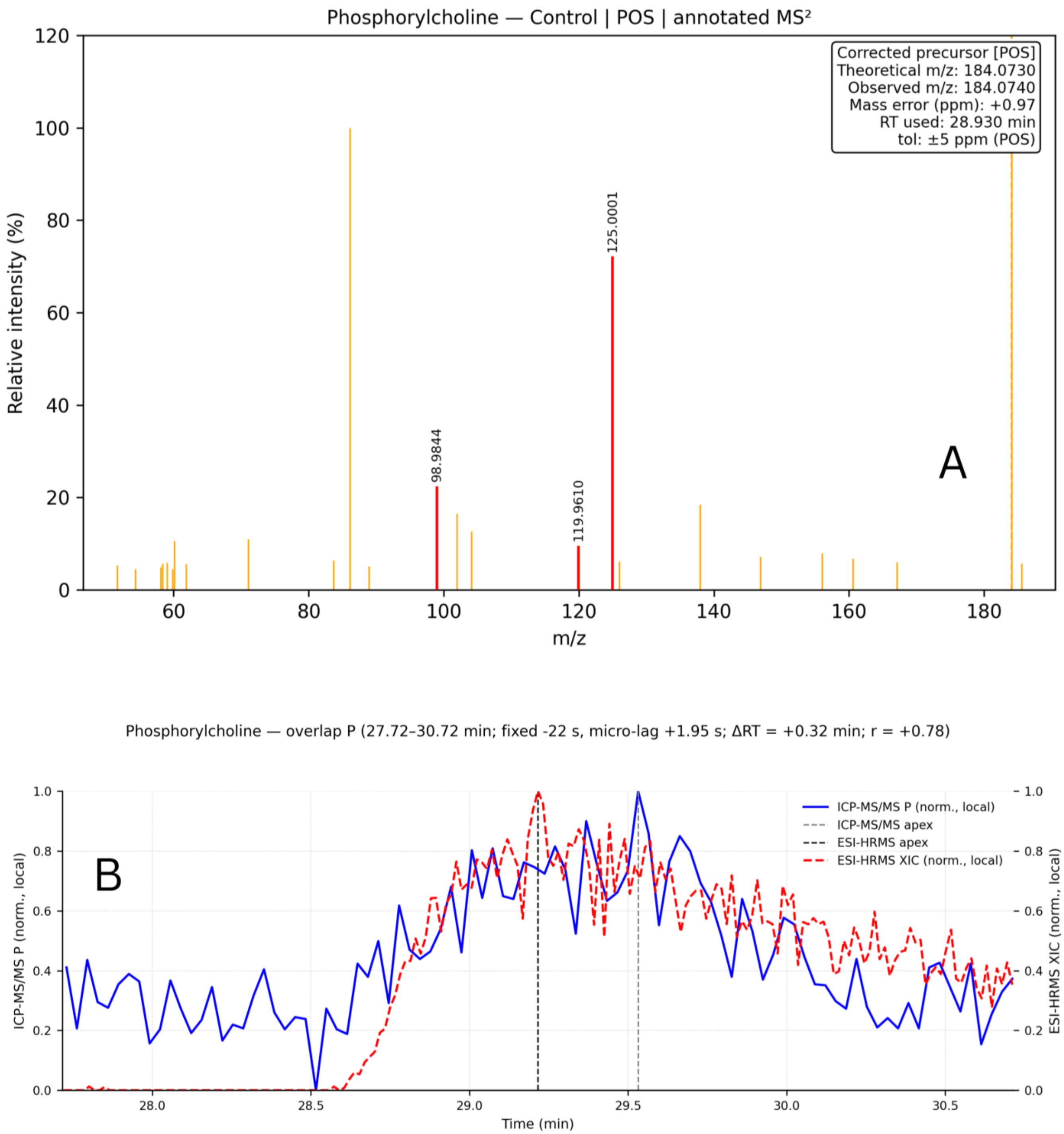


**Figure S9. Phosphorylcholine detected in the control callus in positive ion mode.** (A) The MS² spectrum confirms the protonated precursor at *m/z* 184.0740 (theoretical 184.0730; +1.5 ppm; RT 29.22 min), with the characteristic choline fragment and phosphate-related ions. (B) The P-specific ICP-MS/MS signal overlaps closely with the HRMS XIC, supporting the assignment as a phosphorylated choline headgroup. Phosphorylcholine is also detected in Au NP-treated callus, indicating that phosphocholine cycling persists under NP exposure.

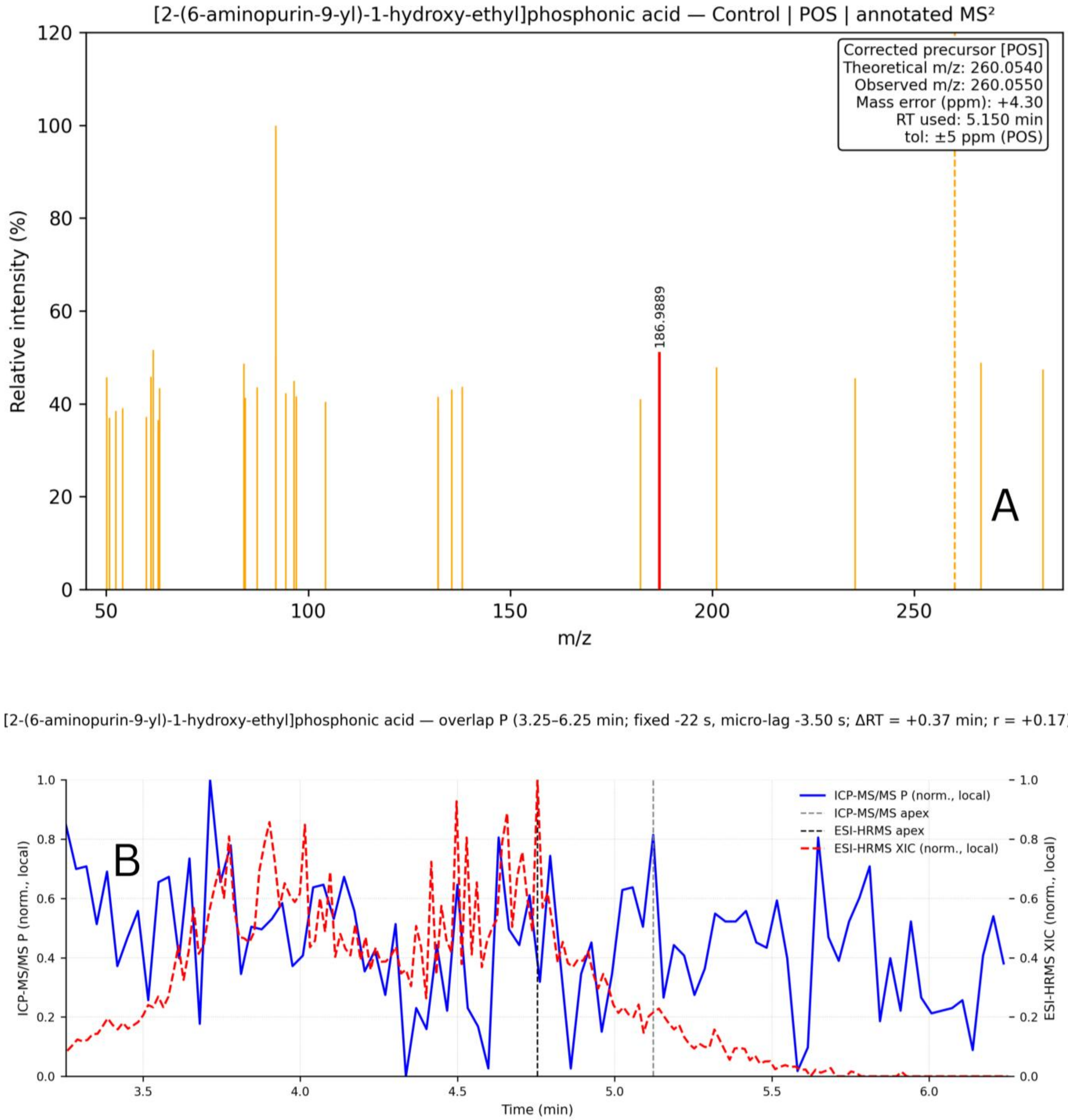


**Figure S10. [2-(6-aminopurin-9-yl)-1-hydroxy-ethyl]phosphonic acid detected in control callus in positive ion mode.** (A) The protonated precursor at *m/z* 260.0540 (theoretical 260.0540; −2.7 ppm; RT 5.35 min) shows a reproducible MS² fragmentation pattern consistent with an aminopurine moiety linked to a phosphonic group. (B) The P ICP-MS/MS signal co-elutes with the HRMS XIC, supporting assignment as a P-containing molecule. This feature was only detected in control samples and did not appear in the Au NP-treated callus. Given its atypical structure and non-biological relevance, this signal was classified as an external contaminant and excluded from the interpretation of treatment-dependent metabolic changes.

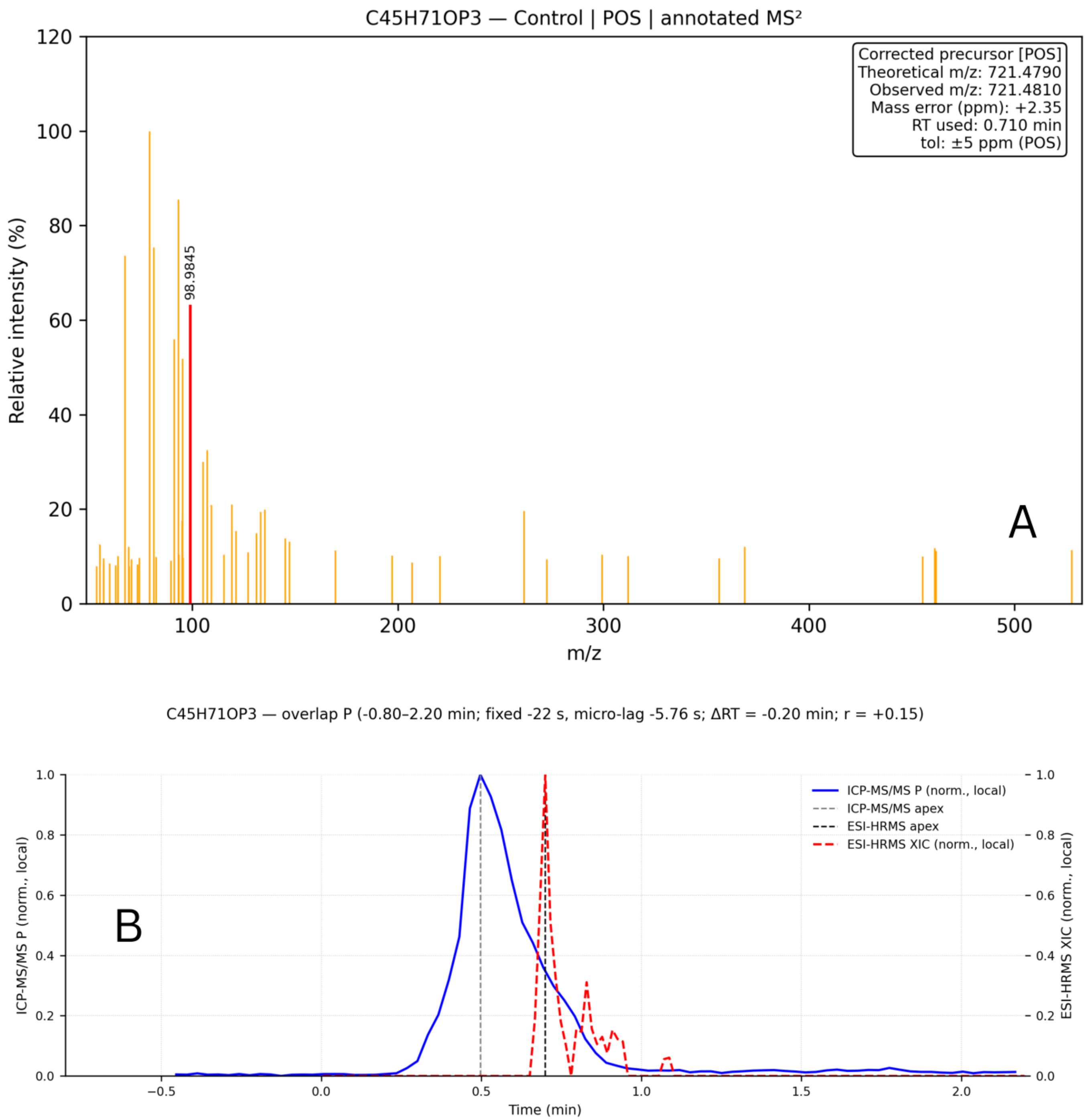


**Figure S11. C45H71OP3 (phospholipid-related intermediate) detected in control callus in positive ion mode.** (A) MS² data confirm the protonated precursor at *m/z* 721.4810 (theoretical 721.4790; +2.4 ppm; RT 0.70 min), with fragments consistent with a multi-phosphorylated lipid-like structure (B). The P ICP-MS/MS signal co-elutes with the HRMS signal, indicating a P-rich lipid intermediate. This signal is not detected in Au NP-treated callus, suggesting that altered high-mass phospholipid intermediates are present under NP stress.

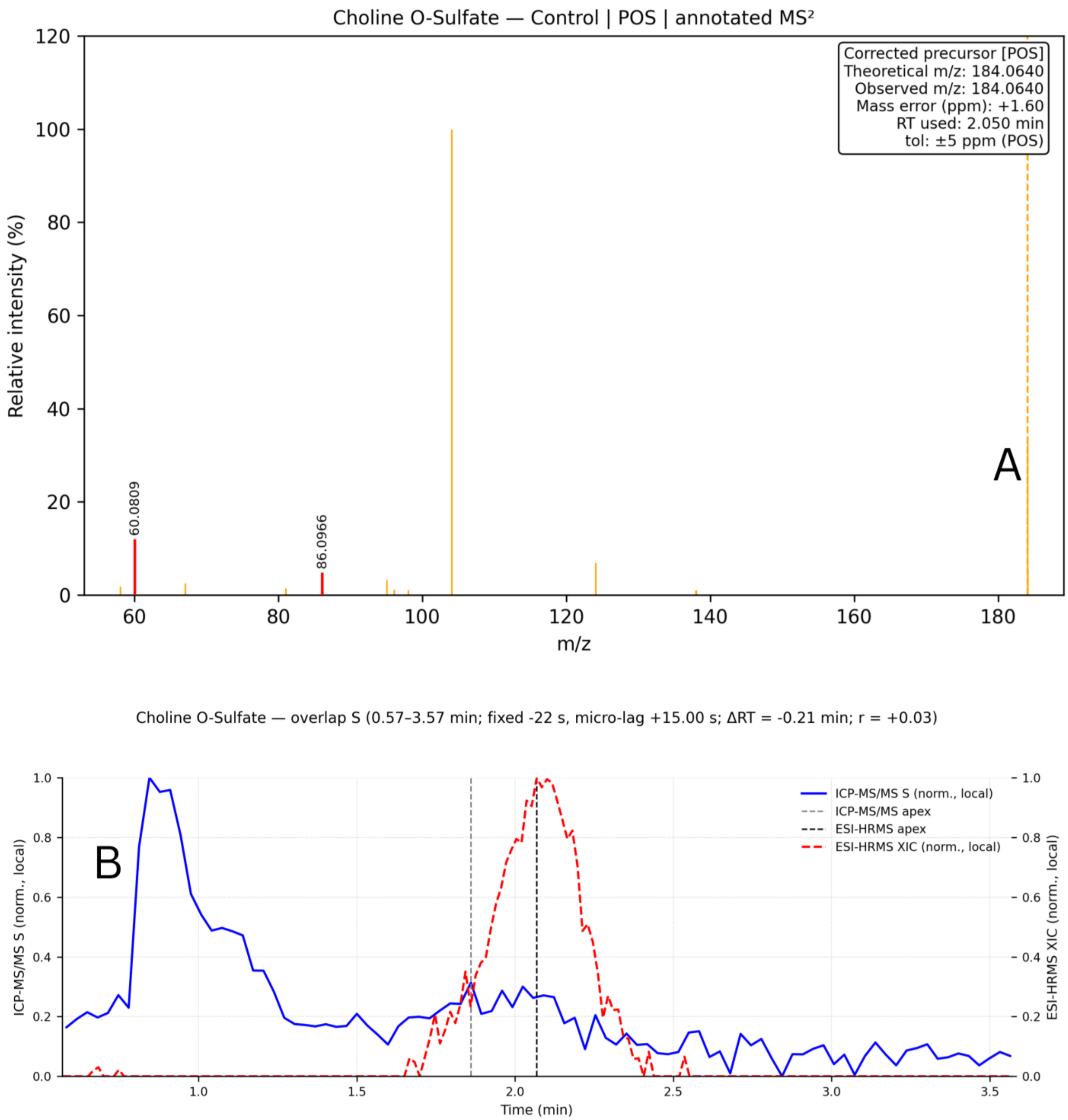


**Figure S12. Choline O-sulfate detected in the control callus in positive ion mode.** (A) The protonated precursor at *m/z* 184.0640 (theoretical 184.0640; +2.1 ppm; RT 2.07 min) shows MS² fragments reflecting a choline core and sulfate-related neutral losses. (B) The sulfur ICP-MS/MS signal overlaps with the HRMS XIC, confirming that the feature carries sulfur. Choline O-sulfate is not observed in Au NP-treated callus, consistent with reorganization of sulfur osmoprotection pathways.

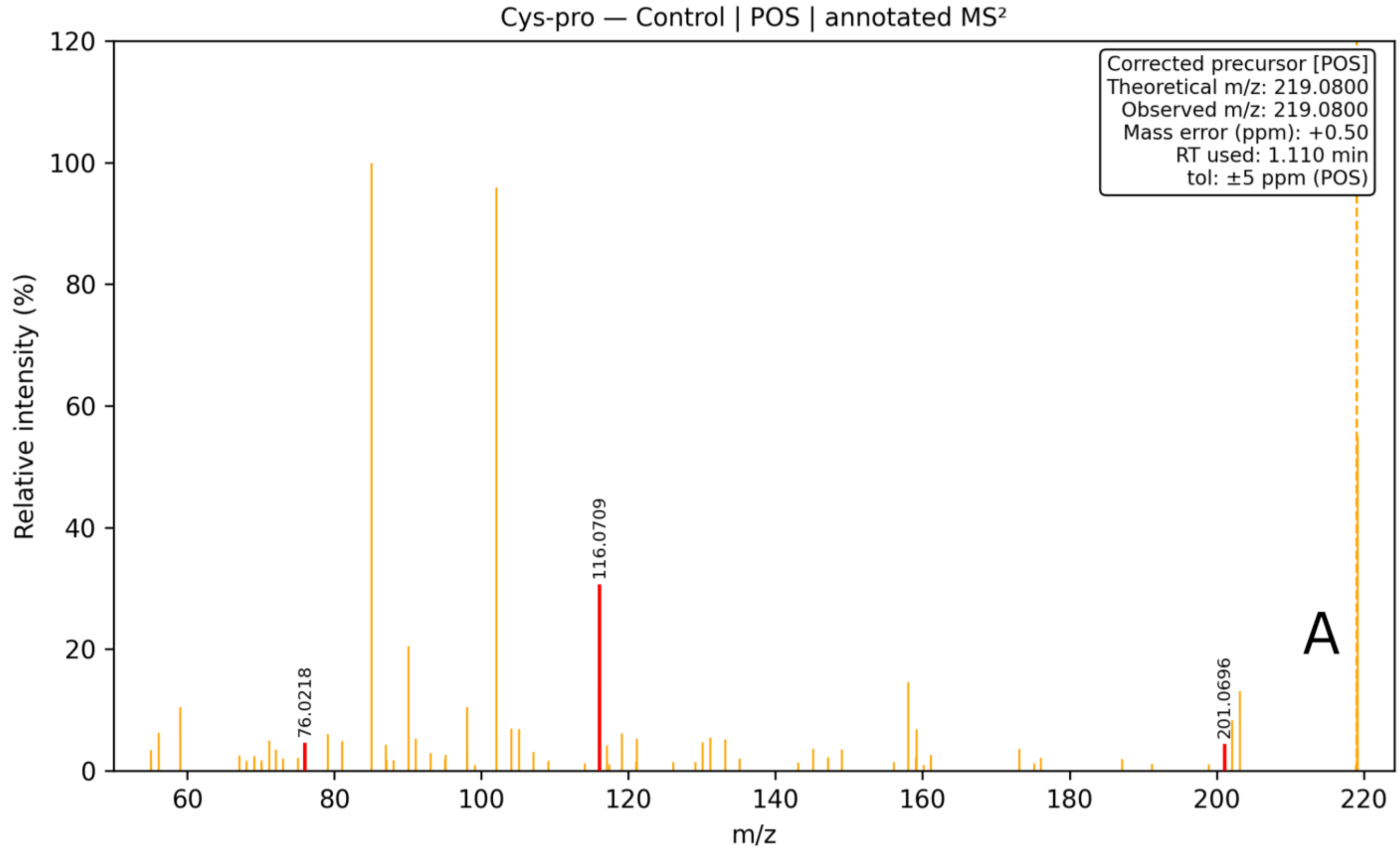


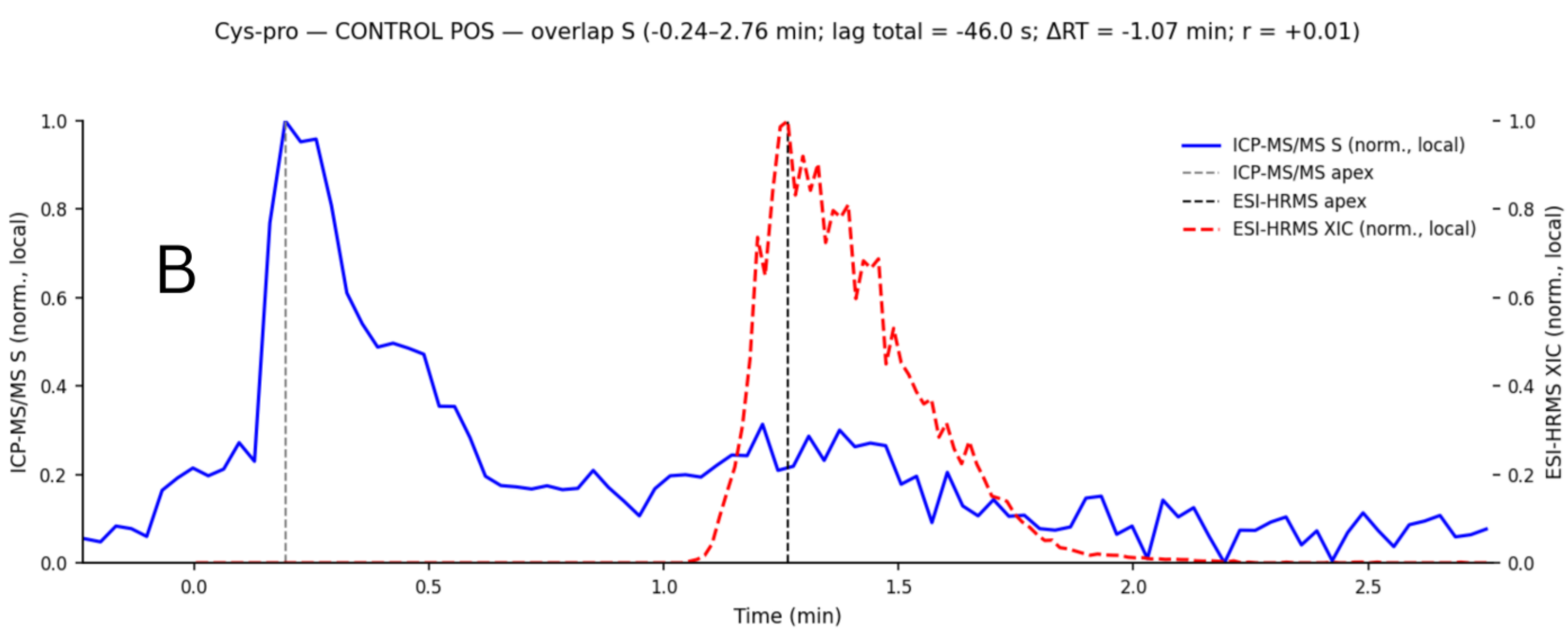


**Figure S13. Cysteine–proline (Cys-Pro) detected in the control callus in positive ion mode.** (A) The protonated precursor at m/z 219.0800 (theoretical 219.0800; +0.7 ppm; RT 1.26 min) yields MS² fragments characteristic of thiol-containing dipeptides, mirroring the fragmentation observed in negative mode. (B) Co-elution of the S ICP-MS/MS signal with the HRMS XIC confirms the S-containing nature of this feature. Cys-Pro is also present in Au NP-treated callus, indicating that this dipeptide persists across treatments.

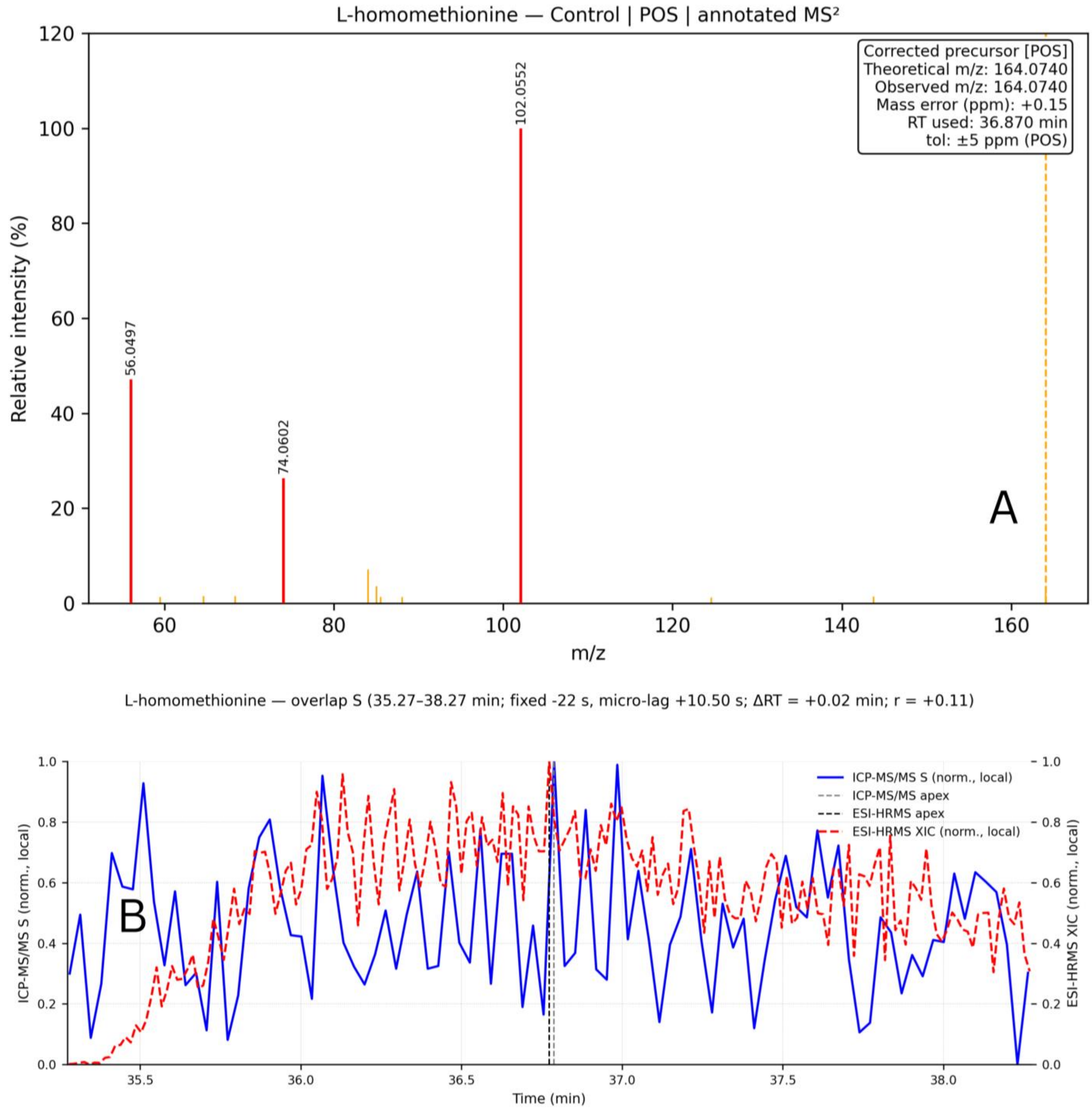


**Figure S14. L-homomethionine detected in the control callus in positive ion mode.** (A) $MS^2$ confirms the protonated precursor at m/z 164.0740 (theoretical 164.0740; +0.5 ppm; RT 36.77 min), with fragment ions consistent with a sulfur-containing amino acid. (B) The S ICP-MS/MS signal co-elutes with the HRMS XIC. Homomethionine is also observed in Au NP-treated callus, supporting its role as a conserved sulfur amino acid.

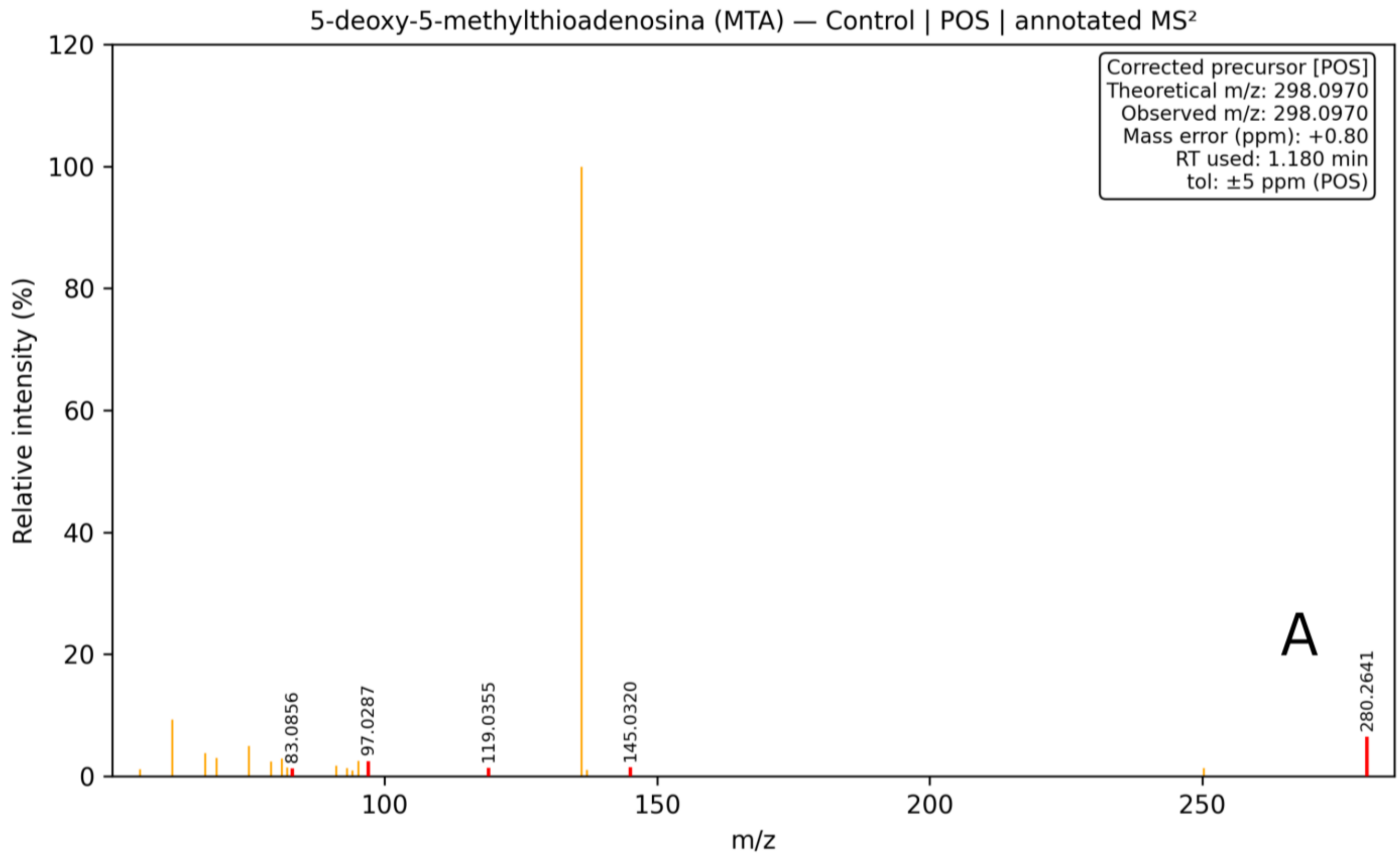


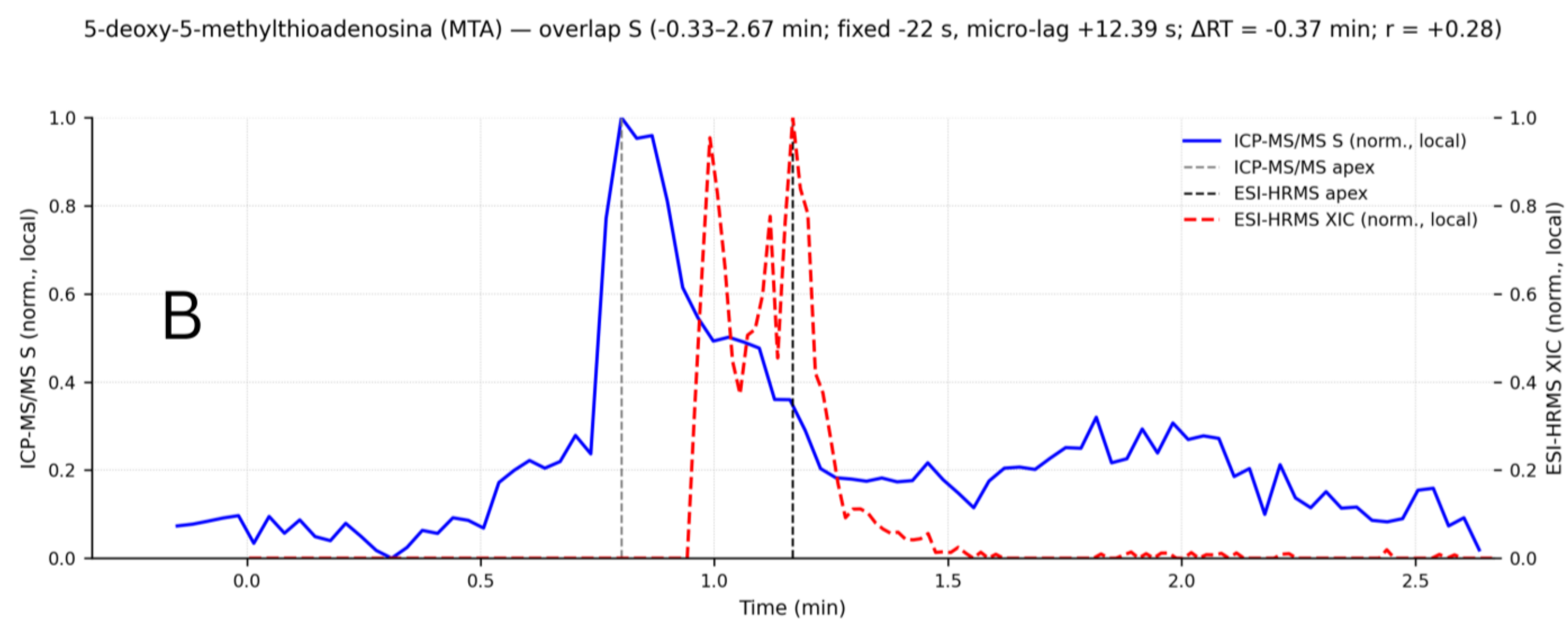


**Figure S15. 5-deoxy-5-methylthioadenosine (MTA) detected in the control callus in positive ion mode.** (A) The protonated precursor at m/z 298.0970 (theoretical 298.0970; +0.8 ppm; RT 1.17 min) shows fragment ions characteristic of an adenine base and methylthio sugar moiety. (B) Co-elution with the S ICP-MS/MS signal confirms the presence of sulfur in this metabolite. MTA was not detected in Au NP-treated callus, suggesting changes in the Yang cycle / methylthio salvage under nanoparticle exposure.

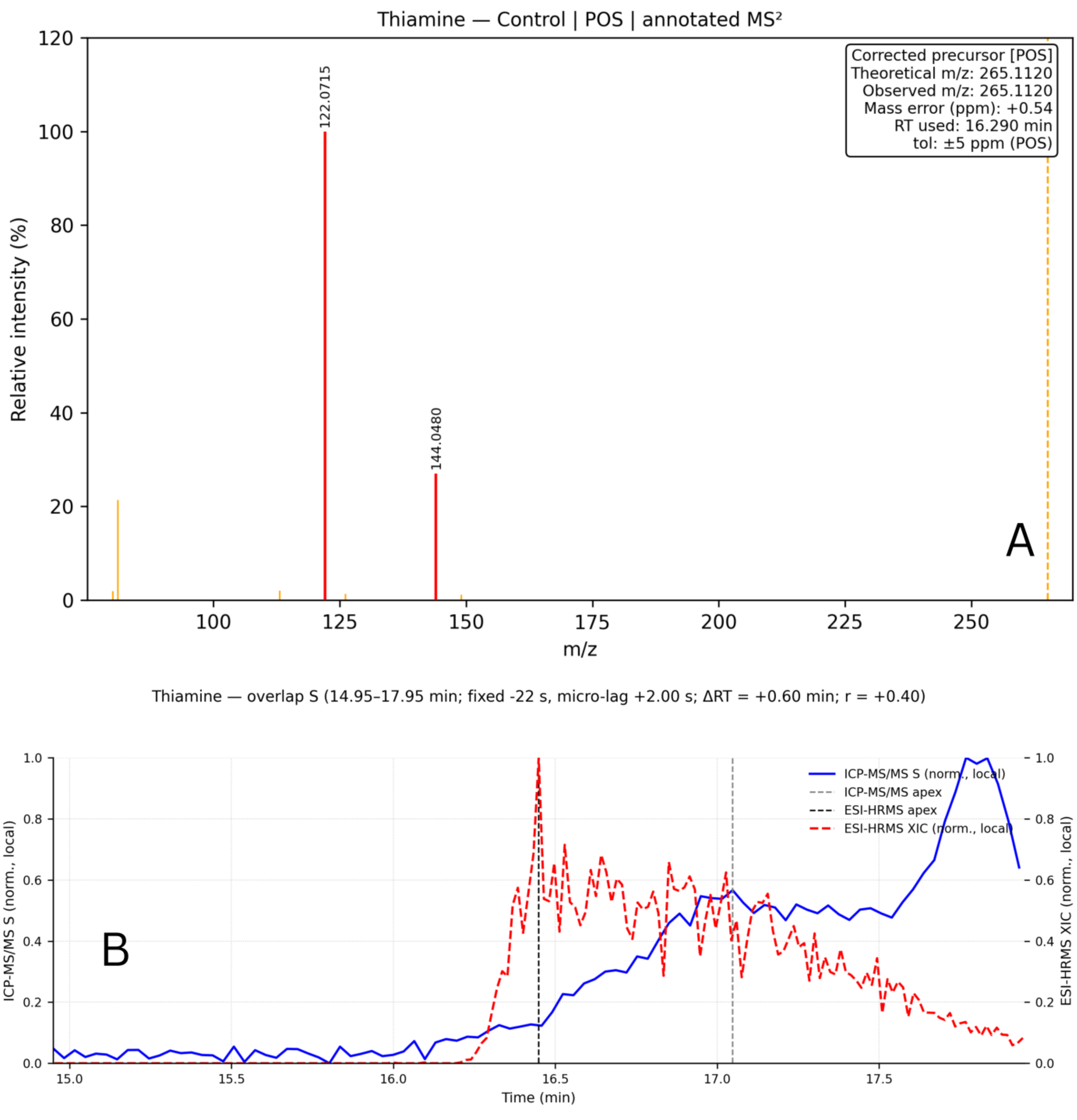


**Figure S16. Thiamine detected in the control callus (positive ion mode).**(A) MS² spectrum of the $[M+H]^+$ ion at m/z 265.1120 (calc. 265.1120; +0.9 ppm; RT 16.45 min), showing fragment ions from the thiazole and aminopyrimidine parts of the molecule. (B) The S ICP-MS/MS signal peaks at the same retention time as the HRMS XIC, confirming that this feature corresponds to an S-containing thiamine species. Thiamine is also present, and more abundant, in Au NP-treated callus, consistent with activation of thiamine-linked stress metabolism.

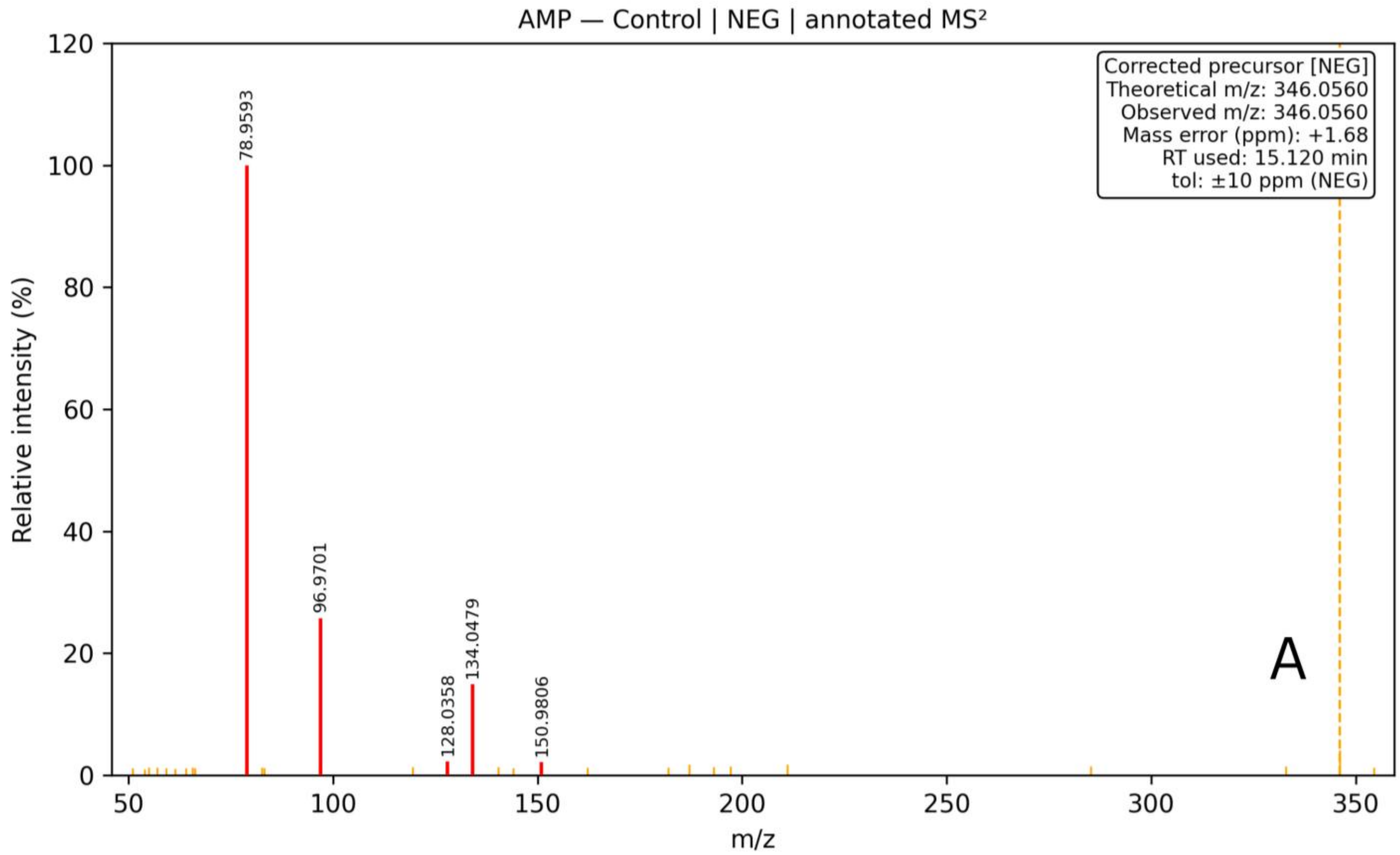


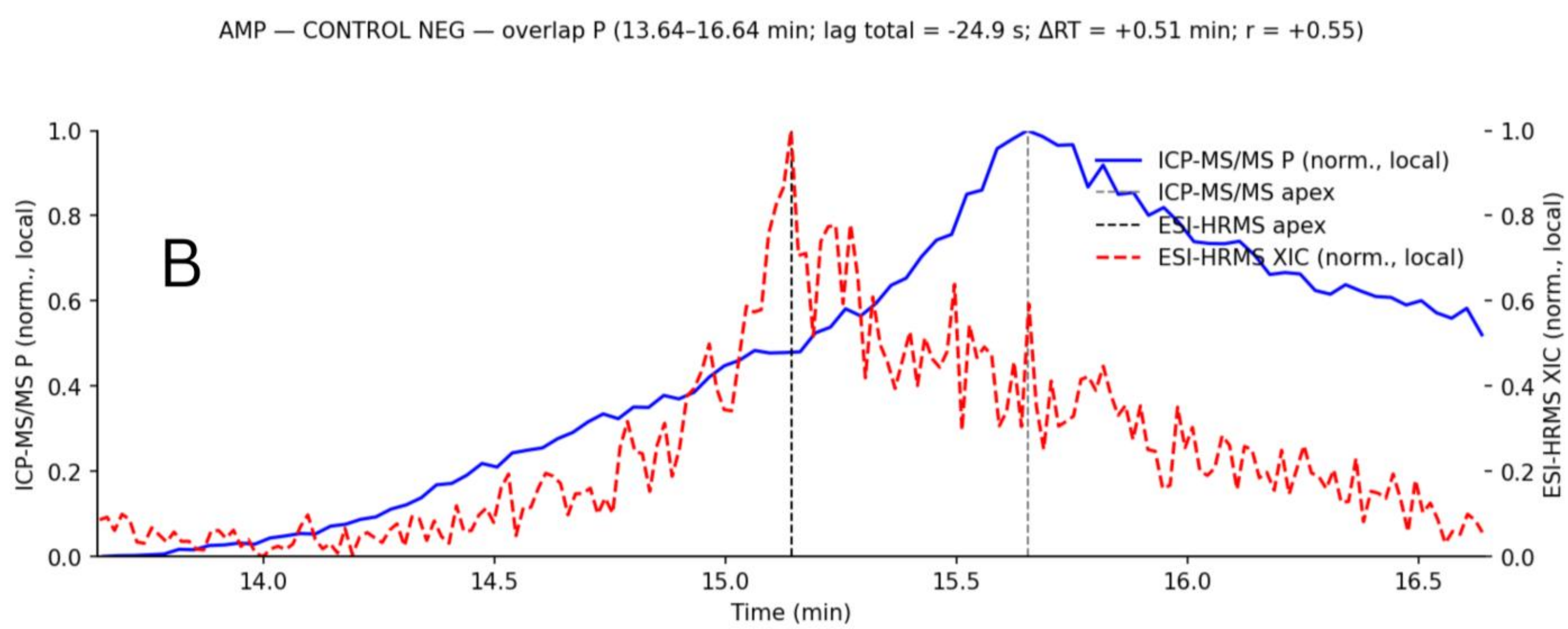


**Figure S17. Adenosine monophosphate (AMP) detected in the control callus in negative ion mode.** (A) MS² confirms the deprotonated precursor at m/z 346.0560 (theoretical 346.0560; +1.8 ppm; RT 15.14 min), with fragment ions characteristic of the adenine base and phosphate-ribose neutral losses. (B) Co-elution of the P ICP-MS/MS signal with the HRMS XIC validates the assignment. AMP is not detected in Au NP-treated samples, suggesting a shift in adenylate or nucleotide pools following NPs exposure.

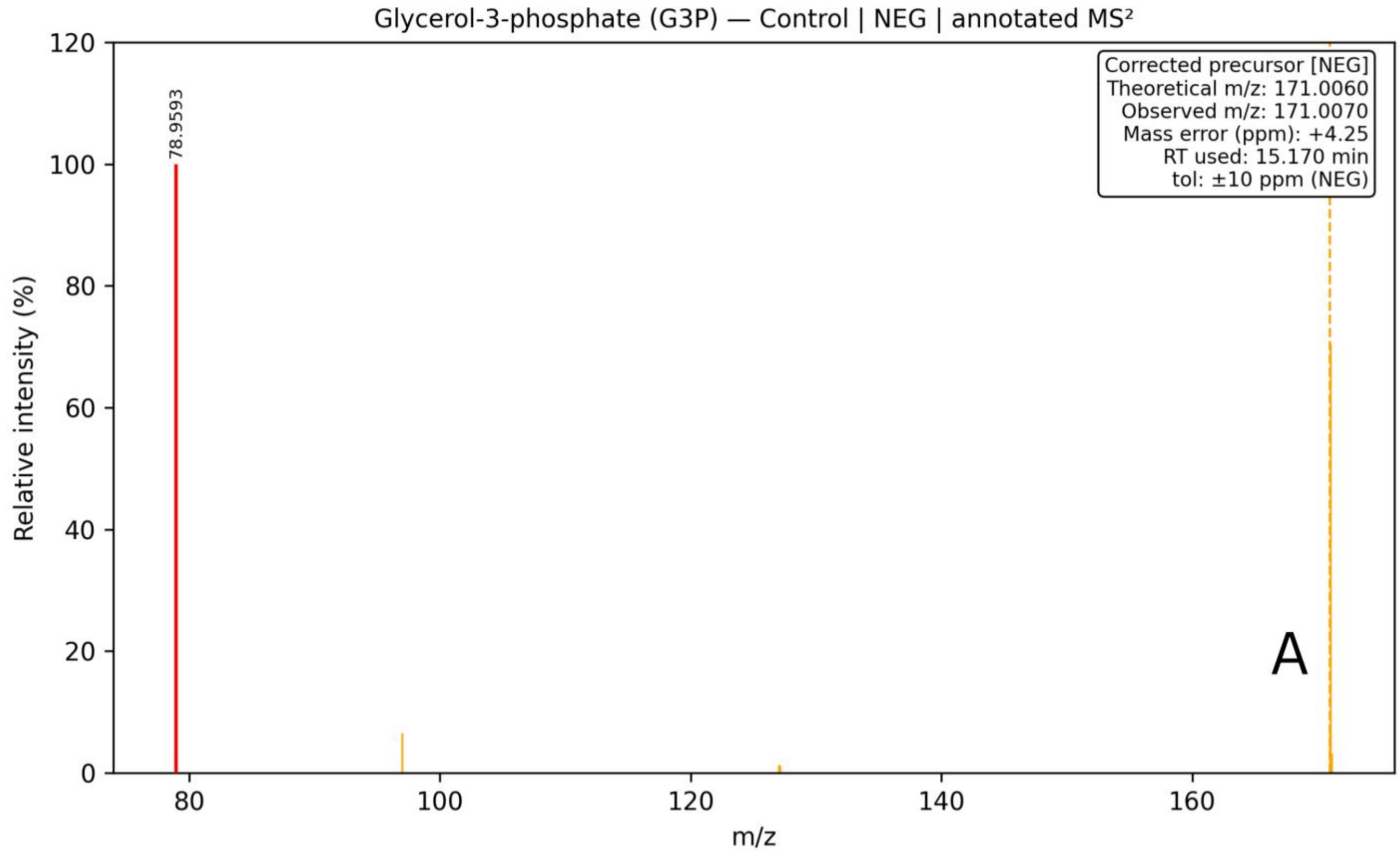


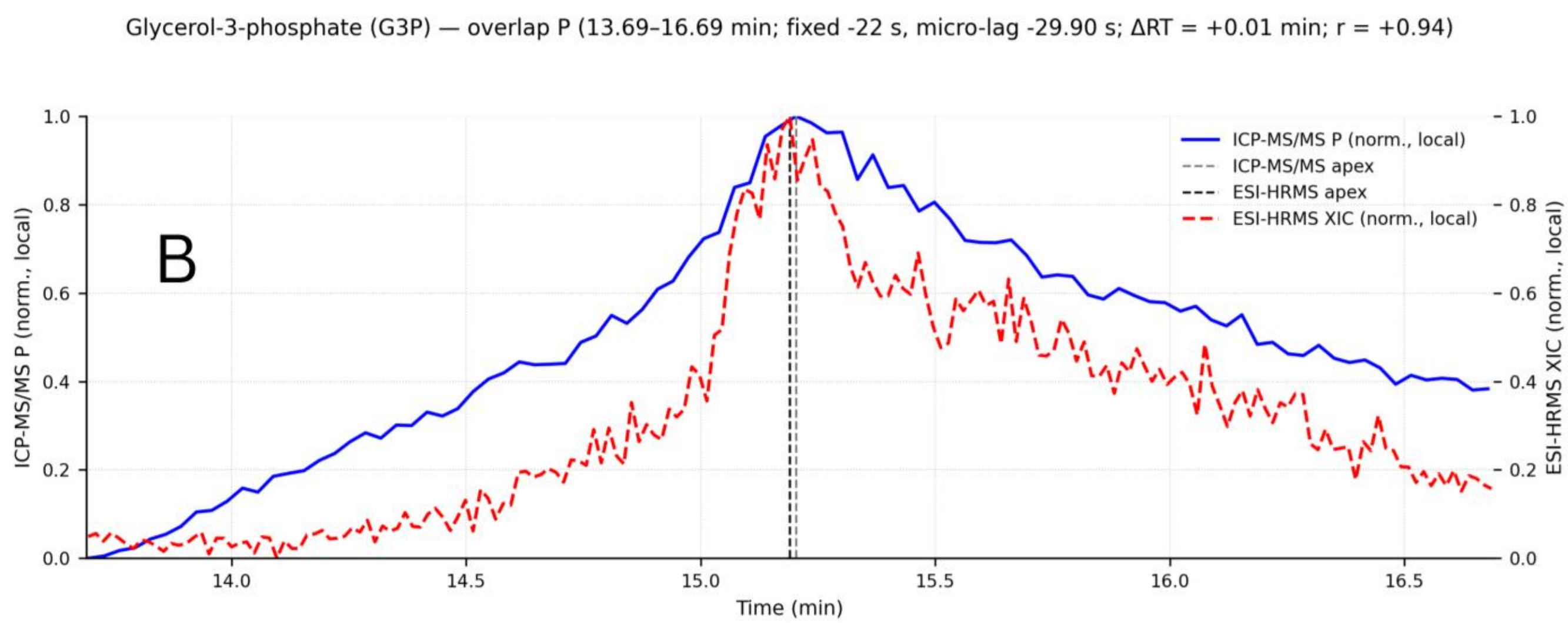


**Figure S18. Glycerol-3-phosphate (G3P) detected in the control callus in negative ion mode.** (A) The deprotonated precursor at m/z 171.0070 (theoretical 171.0060; +4.5 ppm; RT 15.19 min) shows MS² fragments consistent with phosphorylated glycerol. (B) The P ICP-MS/MS signal co-elutes with the HRMS XIC. G3P is also detected in Au NP-treated callus, indicating that this phosphorylated glycolytic intermediate is maintained across conditions.

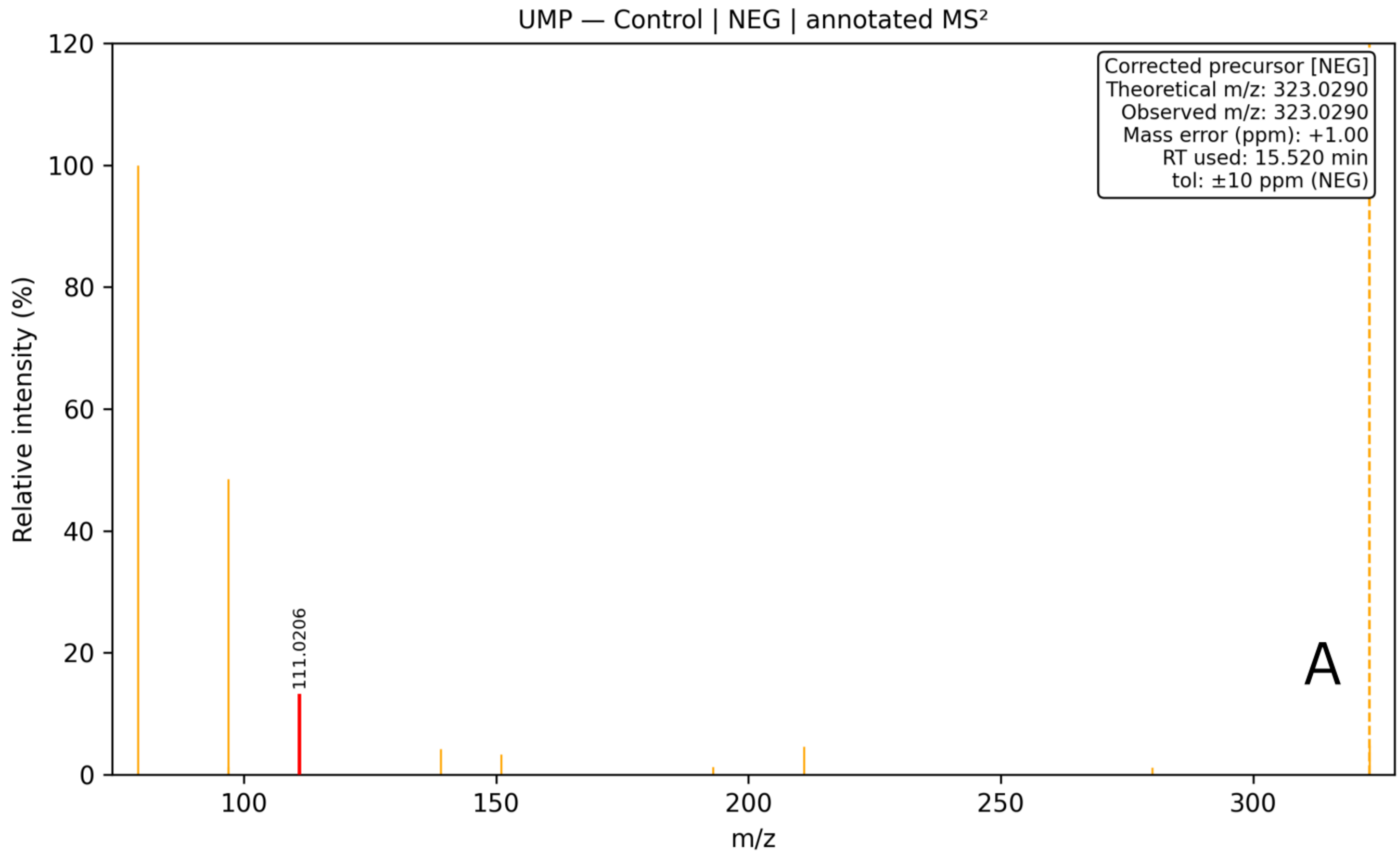


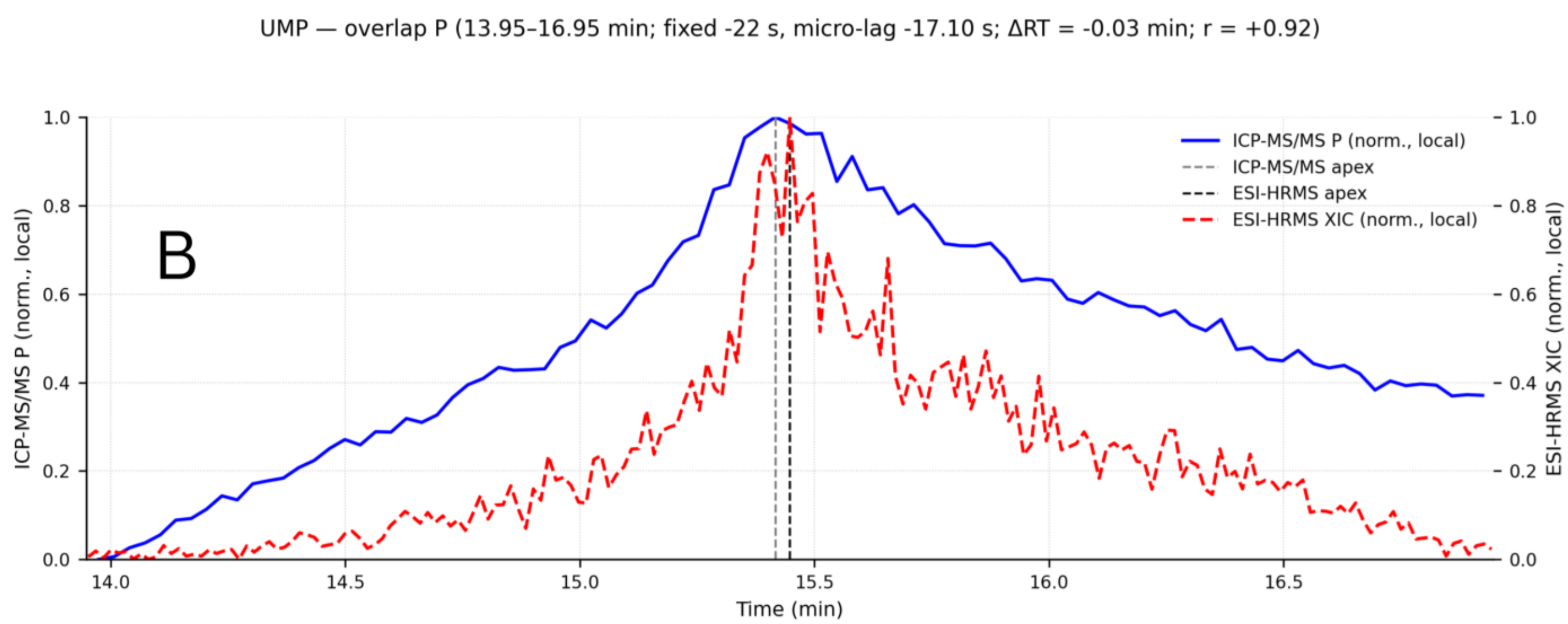


**Figure S19. Uridine monophosphate (UMP) detected in the control callus in negative ion mode.** (A) MS² confirms the deprotonated precursor at m/z 323.0290 (theoretical 323.0290; +1.3 ppm; RT 15.45 min), with fragment ions reflecting loss of ribose and phosphate. (B) Co-elution of the P ICP-MS/MS signal with the HRMS XIC supports the nucleotide assignment. UMP is also present in Au NP-treated callus, suggesting preserved uridylate pools.

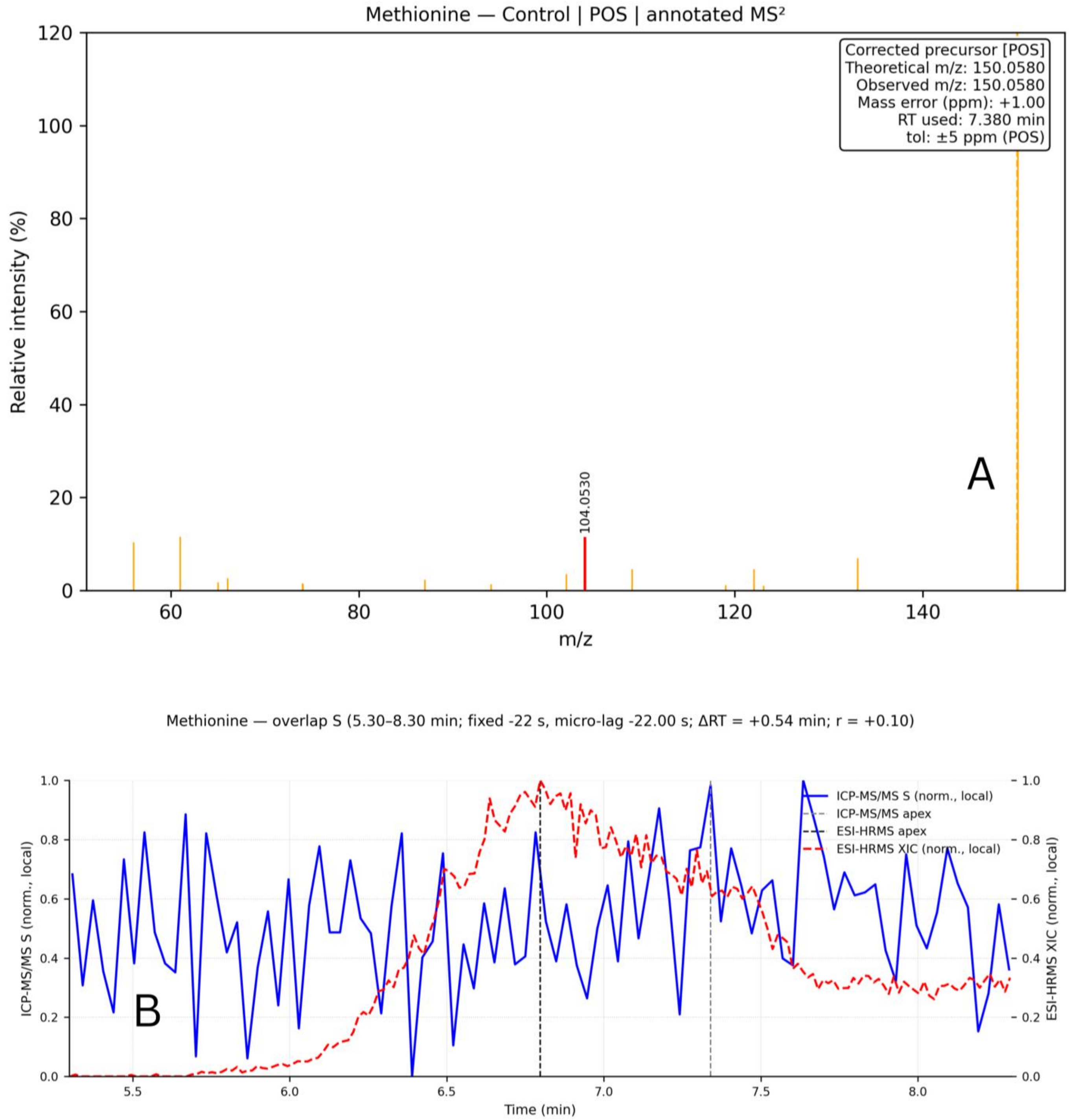


**Figure S20. Methionine detected in the control callus in positive ion mode.** (A) The protonated precursor at m/z 150.0590 (theoretical 150.0580; +2.7 ppm; RT 6.80 min) generates MS² fragments typical of an aliphatic thioether amino acid. (B) The sulfur ICP-MS/MS signal overlaps with the HRMS XIC, confirming the presence of sulfur. Methionine is also detected in Au NP-treated callus, consistent with conserved sulfur-amino acid metabolism.

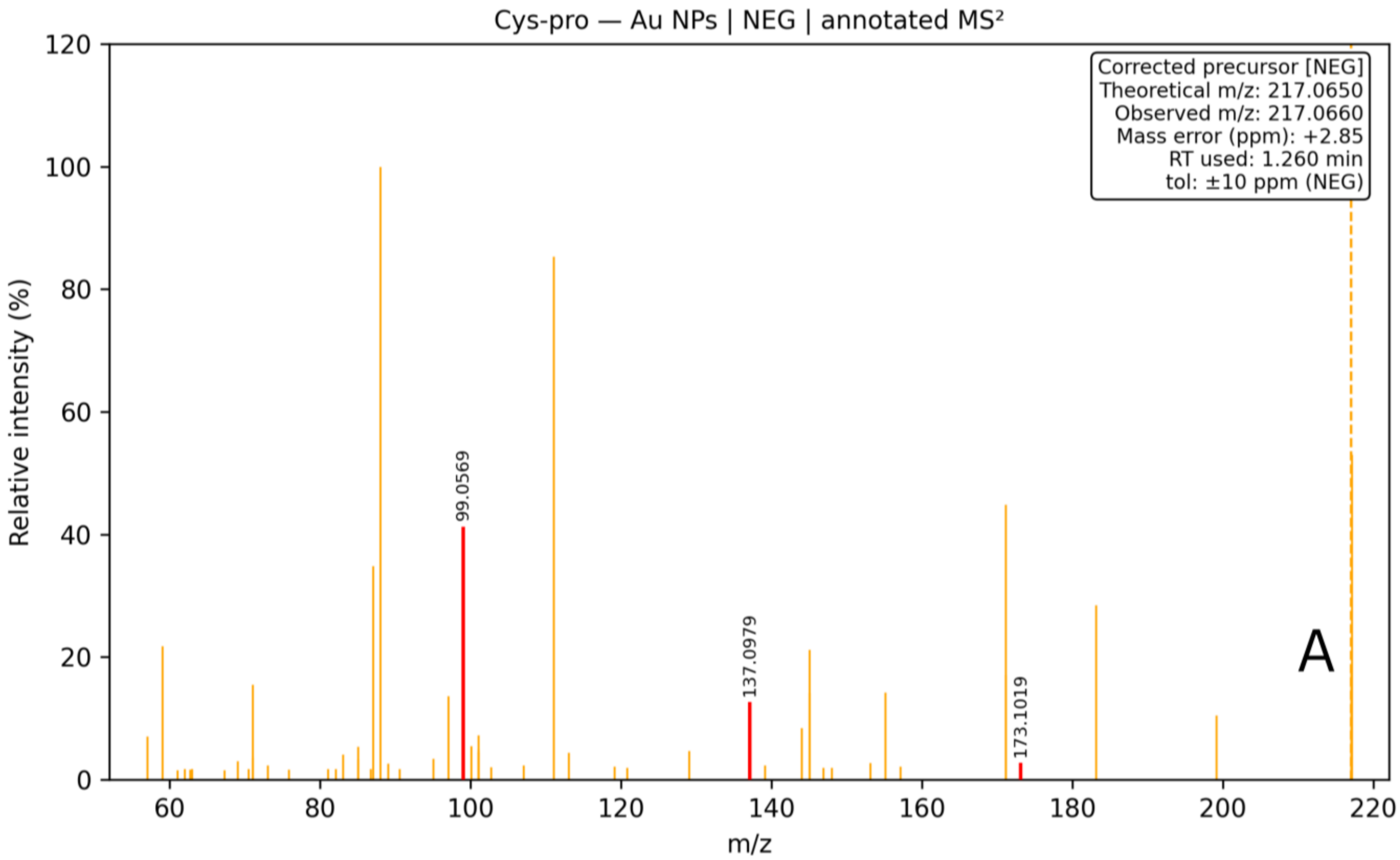


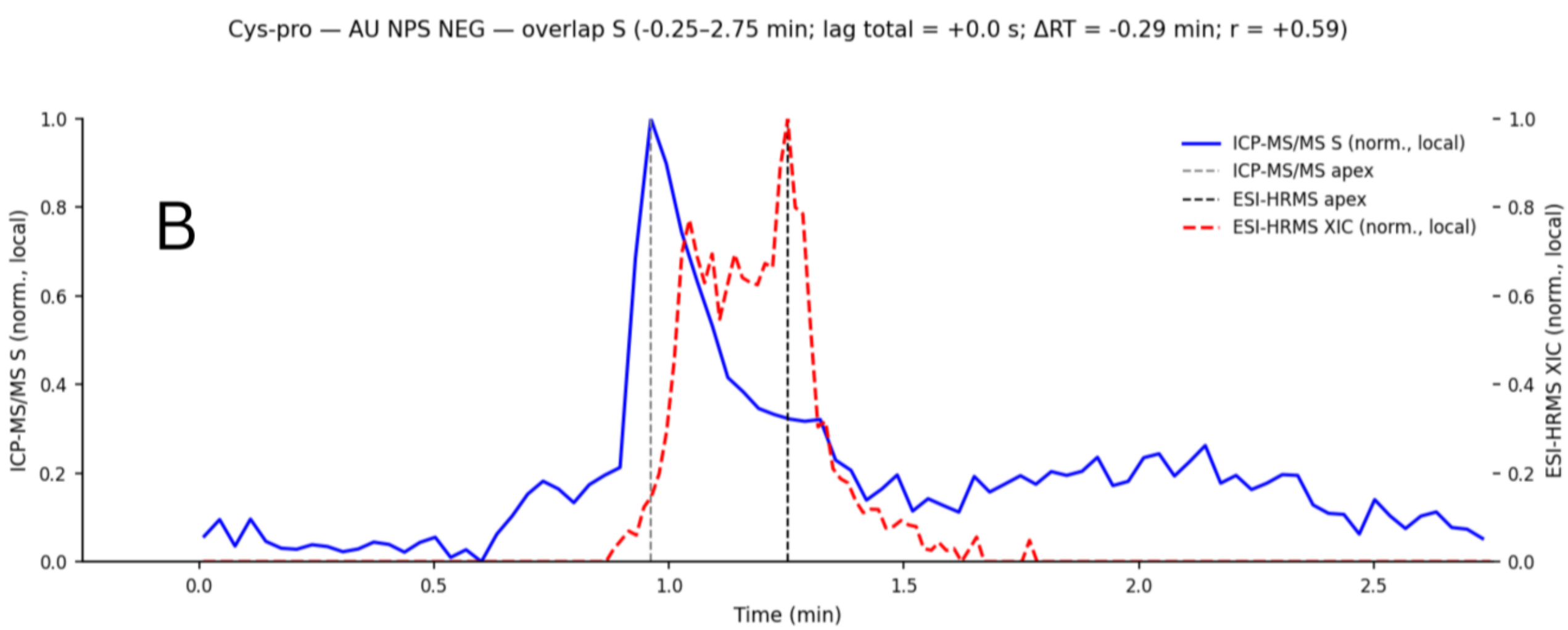


**Figure S21. Cysteine–proline (Cys-Pro) detected in Au NP-treated callus in negative ion mode. (A)** The deprotonated precursor at *m/z* 217.0660 (theoretical 217.0650; +2.6 ppm; RT 1.25 min) shows an MS² pattern matching that observed in control samples. (B) The S ICP-MS/MS signal co-elutes with the HRMS XIC. Together with detection in controls, this indicates that Cys-Pro is a persistent thiol-containing dipeptide under exposure to Au NPs.

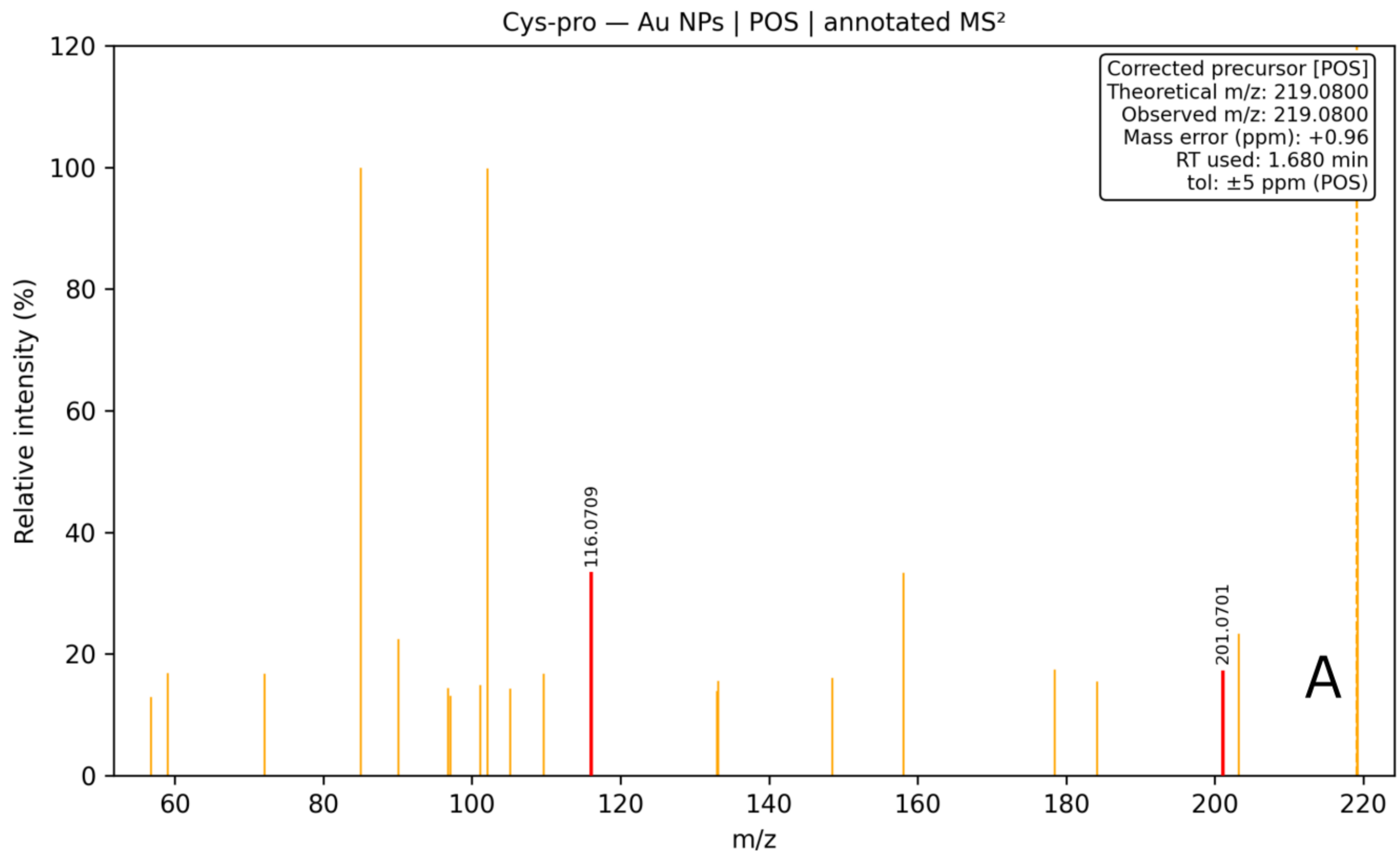


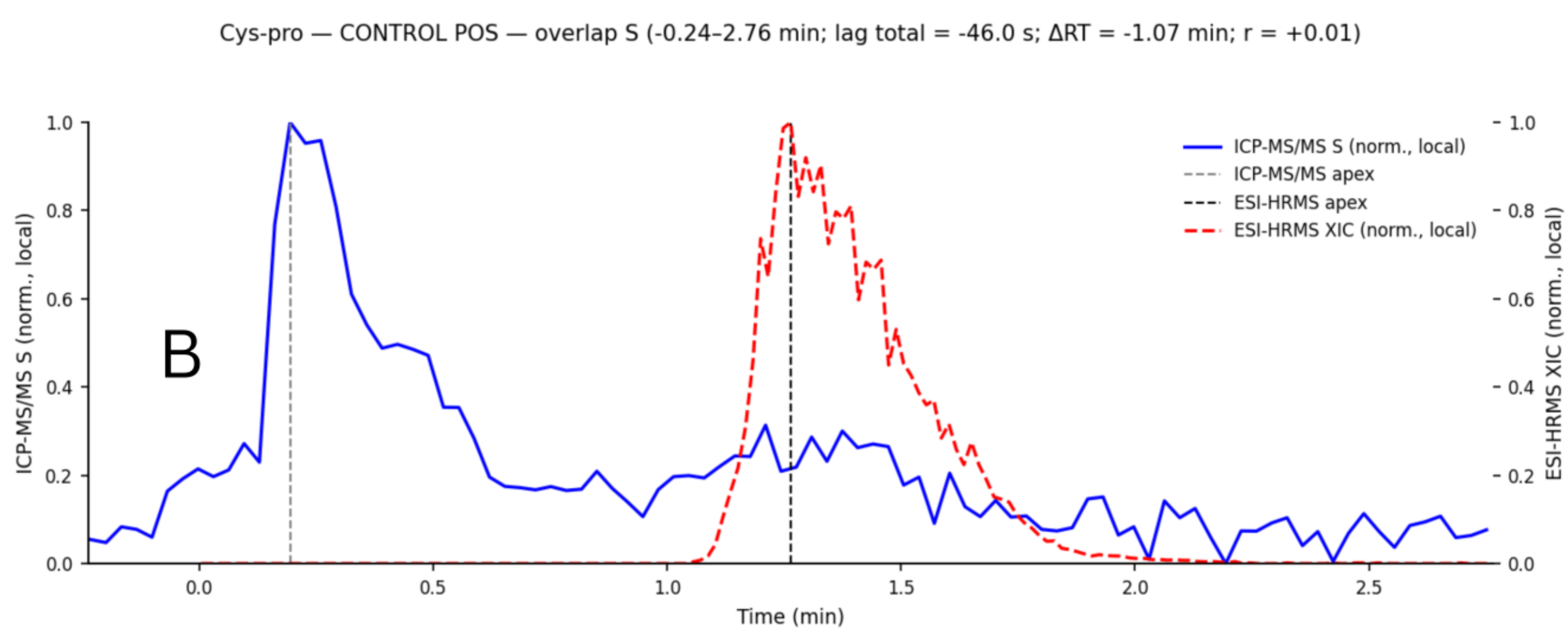


**Figure S22. Cysteine–proline (Cys-Pro) detected in Au NP-treated callus in positive ion mode.** (A) The protonated precursor at *m/z* 219.0800 (theoretical 219.0800; +1.5 ppm; RT 1.23 min) displays the same key fragments as in the control sample. (B) Co-elution of the S ICP-MS/MS signal with the HRMS XIC confirms sulfur content. The persistence of Cys-Pro in both treatments suggests robust thiol dipeptide metabolism.

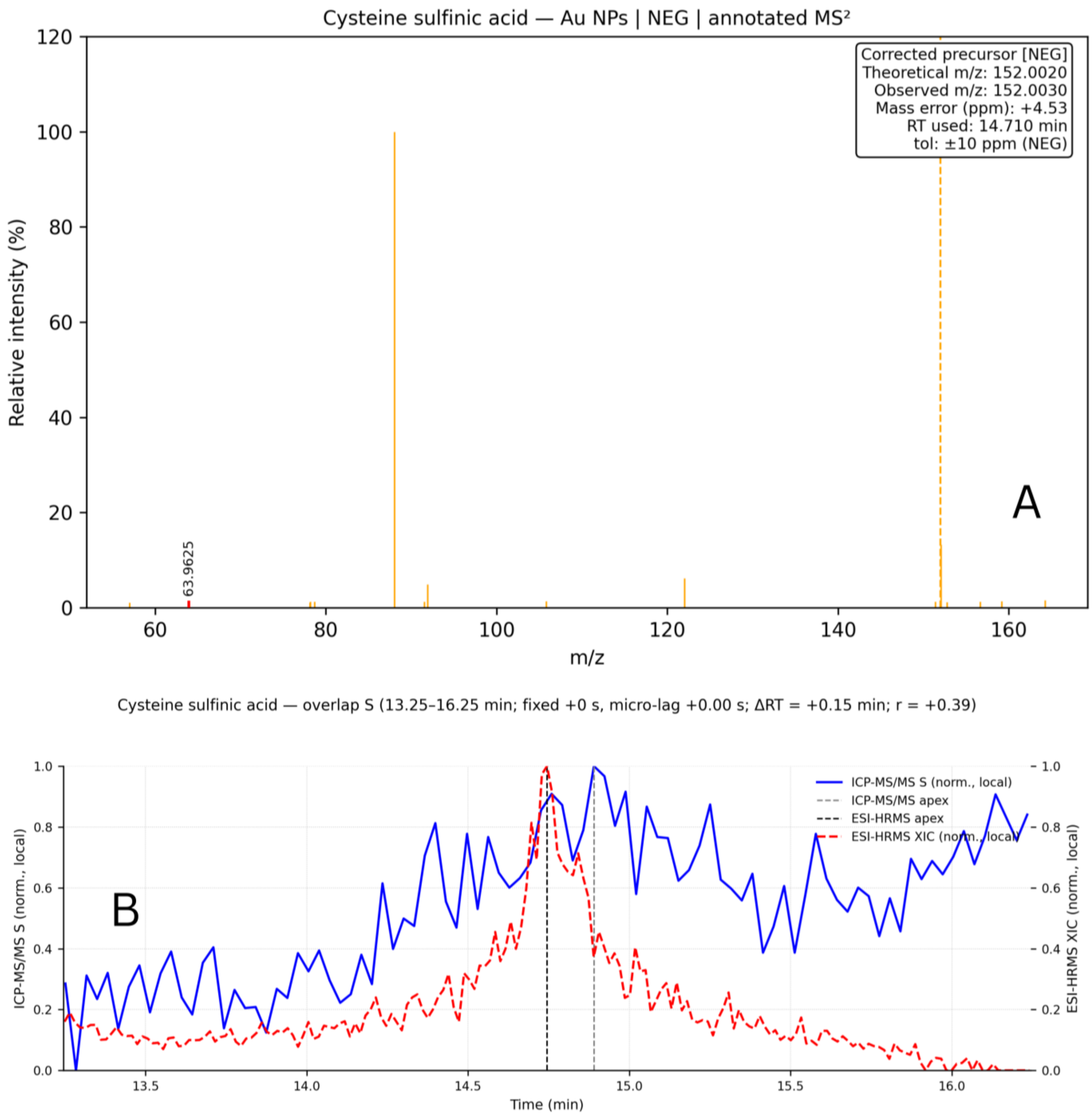


**Figure S23. Cysteine sulfinic acid detected in the Au NP-treated callus in negative ion mode.** (A) The [M+Cl]⁻ adduct at m/z 152.0030 (theoretical 152.0020; +4.4 ppm; RT 14.75 min) gives MS² fragments consistent with a sulfinic acid derivative of cysteine. (B) The S ICP-MS/MS signal aligns with the HRMS XIC, confirming the incorporation of S. This metabolite is absent in controls, indicating oxidation of cysteine side chains under Au NP-induced.

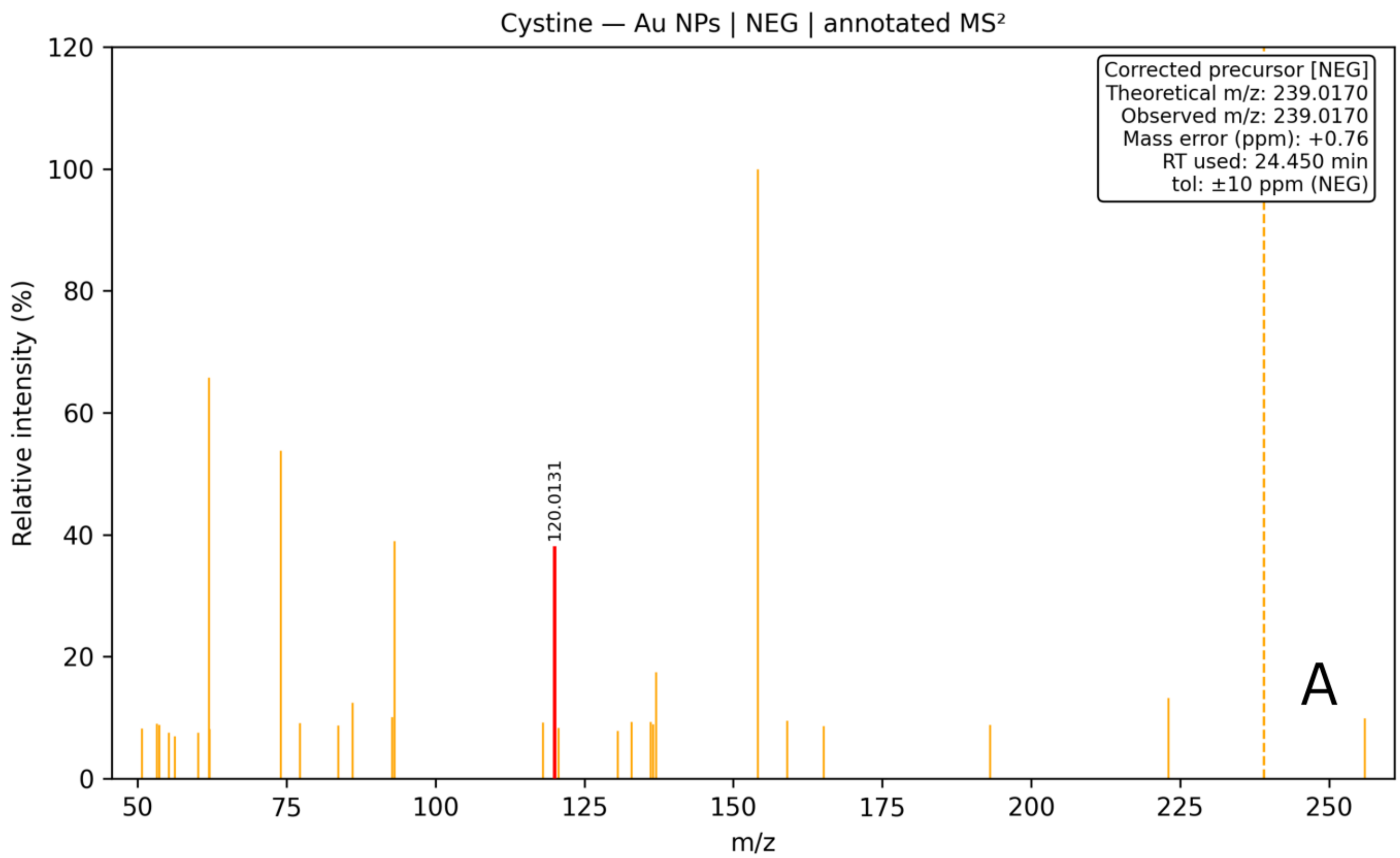


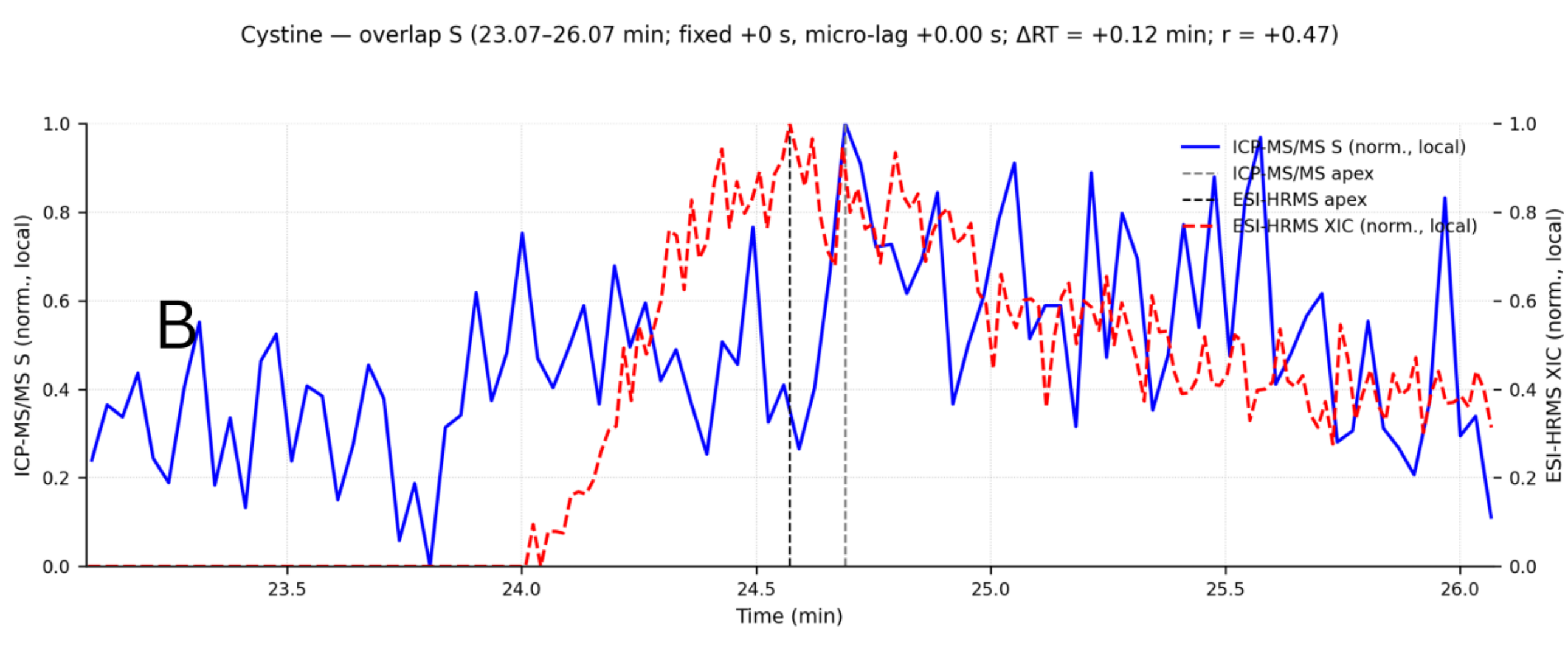


**Figure S24. Cystine detected in Au NP-treated callus in negative ion mode.** (A) MS² confirms the deprotonated precursor at m/z 239.0170 (theoretical 239.0170; +1.0 ppm; RT 24.57 min), with fragments characteristic of disulfide bond cleavage. (B) Co-elution of the S ICP-MS/MS signal with the HRMS XIC validates the assignment. Cystine is not detected in controls, consistent with the formation of disulfide-linked oxidized sulfur pools upon exposure to Au NPs.

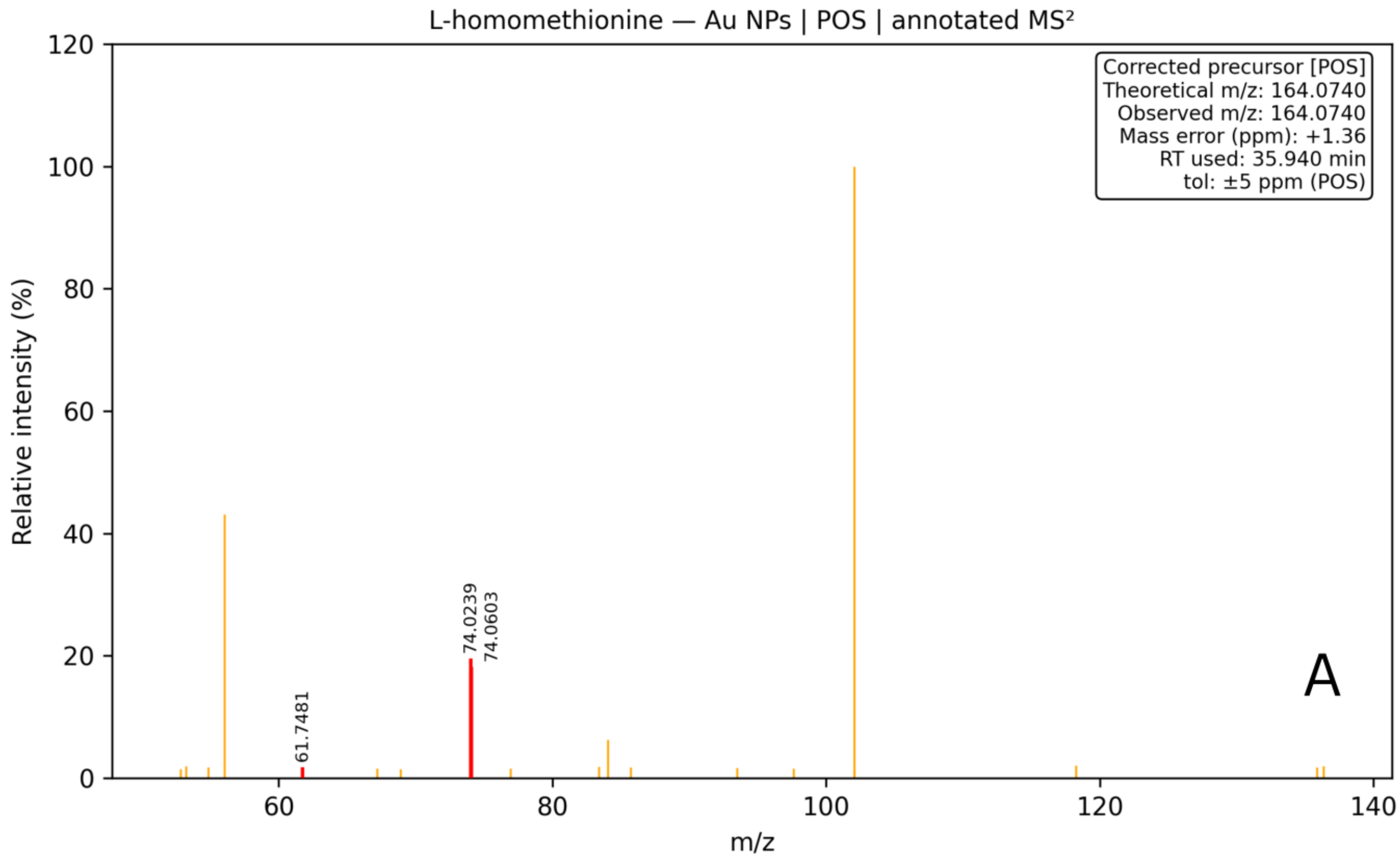


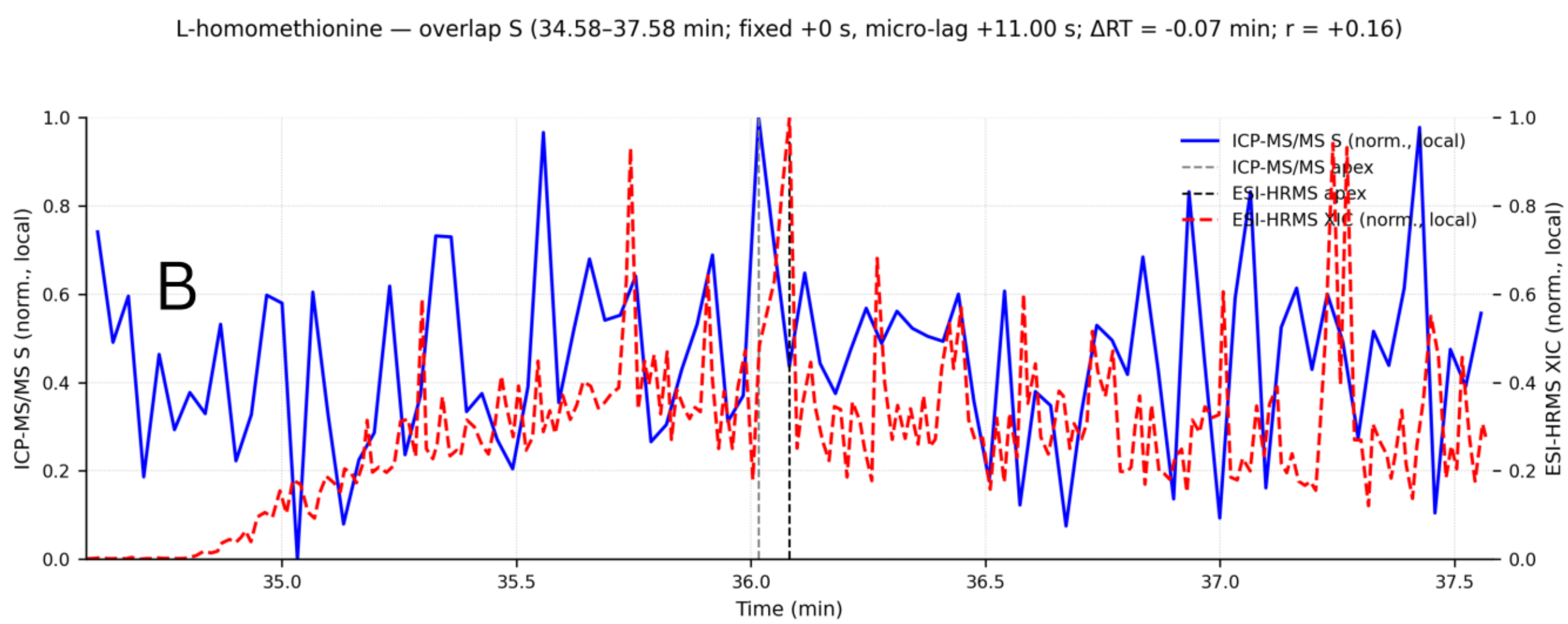


**Figure S25. L-homomethionine detected in Au NP-treated callus in positive ion mode.** (A) The protonated precursor at m/z 164.0740 (theoretical 164.0740; +1.3 ppm; RT 36.08 min) fragments in a manner consistent with an S-containing amino acid, similarly to the control.(B) The S ICP-MS/MS signal co-elutes with the HRMS XIC. Detection in both treatments indicates that homomethionine metabolism is maintained under Au NP exposure.

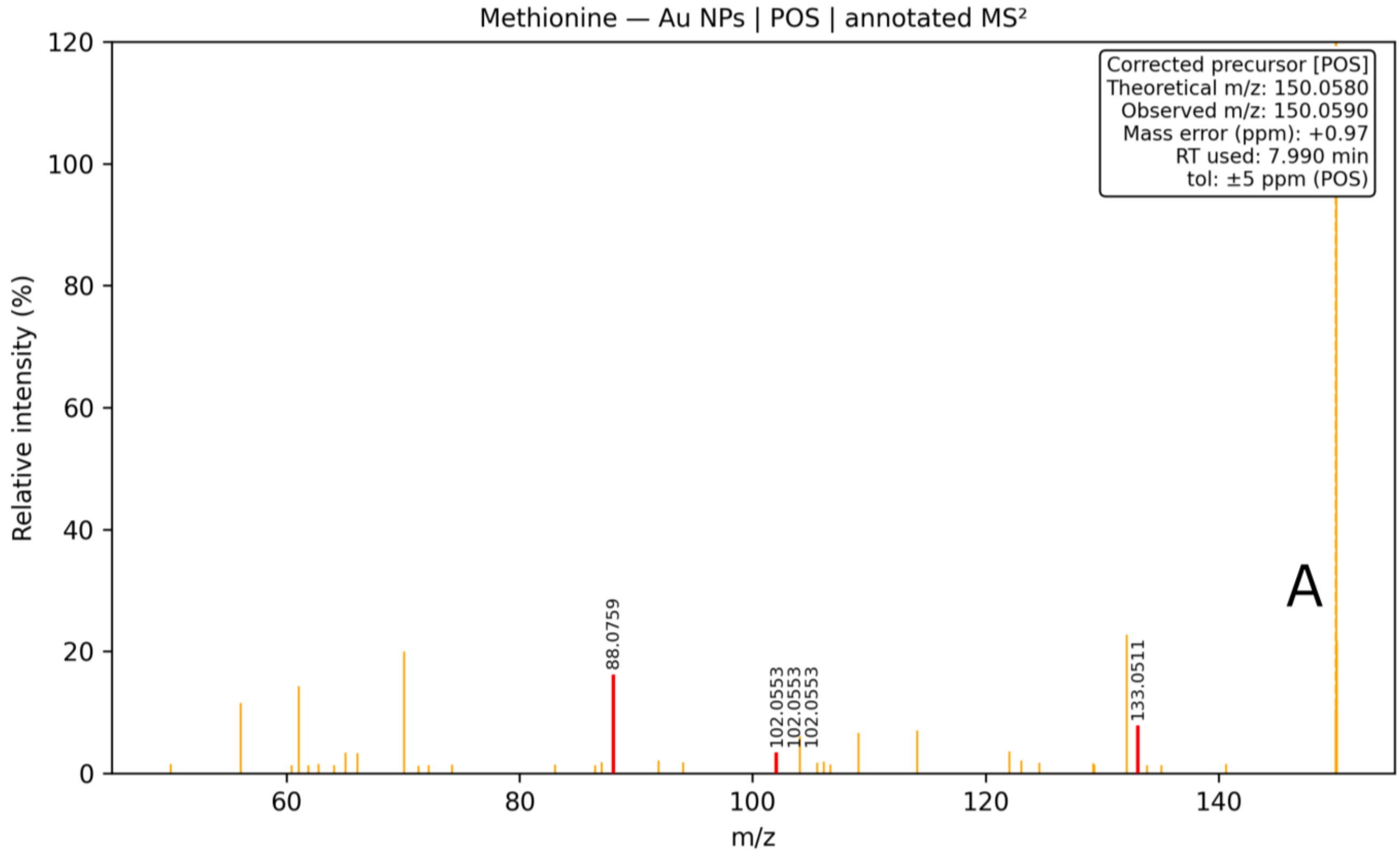


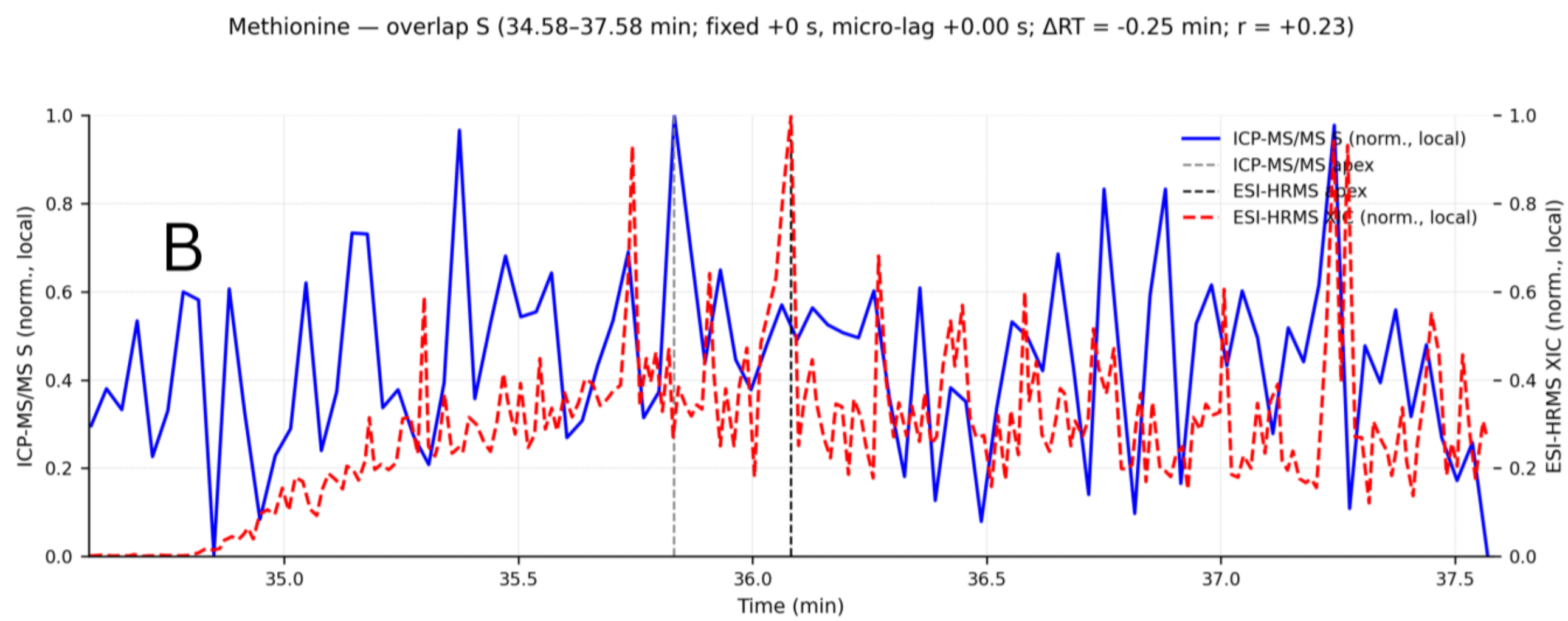


**Figure S26. Methionine detected in the Au NP-treated callus in positive ion mode.** (A) The protonated precursor at m/z 150.0590 (theoretical 150.0580; +2.6 ppm; RT 7.25 min) yields MS² fragments comparable to those in control samples. (B) The S ICP-MS/MS signal overlaps with the HRMS XIC. Methionine is present in both groups, but shows lower intensity in the Au NP-treated callus, consistent with the consumption or oxidation of reduced sulfur pools.

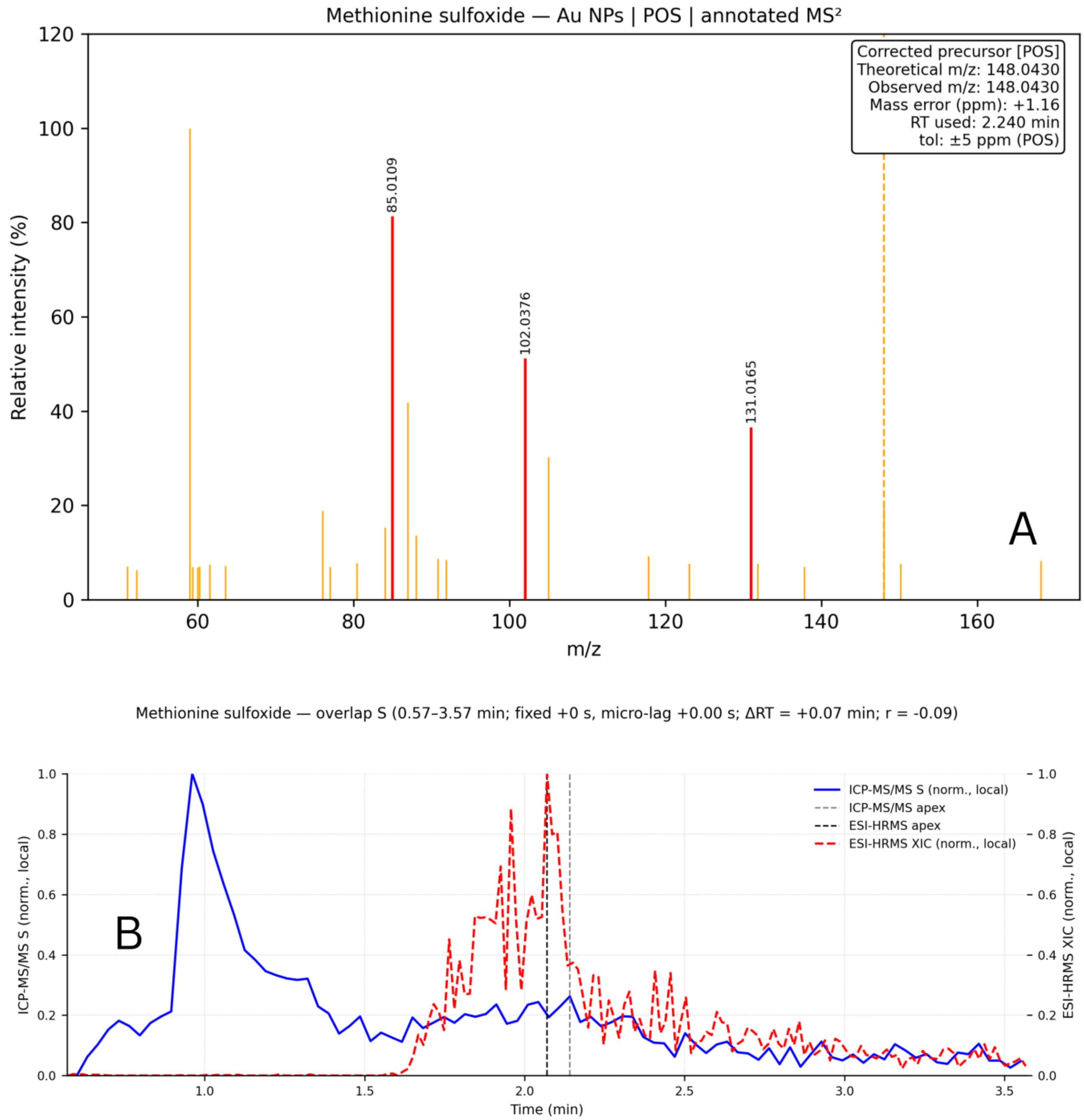


**Figure S27. Methionine sulfoxide detected in Au NP-treated callus in positive ion mode.** (A) The dehydrated protonated precursor $[M+H-H_2O]^+$ at m/z 148.0430 (theoretical 148.0430; +0.6 ppm; RT 2.07 min) displays MS² fragments typical of oxidized methionine. (B) The S ICP-MS/MS signal co-elutes with the HRMS XIC. This oxidized S amino acid is unique to Au NP-treated callus, indicating oxidative modification of methionine.

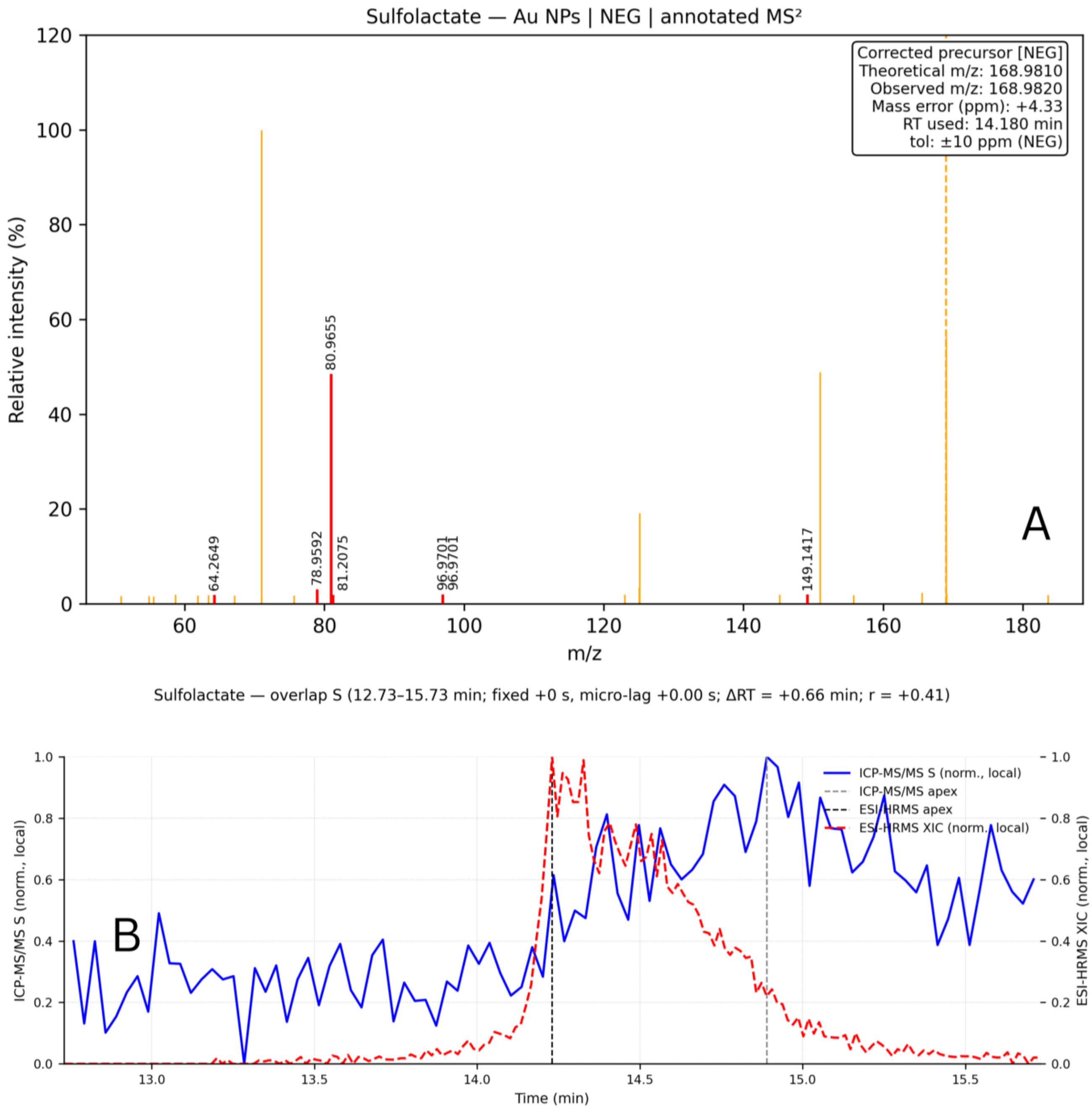


**Figure S28. Sulfolactate detected in Au NP-treated callus in negative ion mode.** (A) MS² confirms the deprotonated precursor at m/z 168.9820 (theoretical 168.9810; +4.2 ppm; RT 14.23 min), with fragments consistent with a sulfonated hydroxyacid. (B) Co-elution of the S ICP-MS/MS signal with the HRMS XIC validates the S-containing assignment. Sulfolactate is absent from the controls, suggesting that Au NP-induced rerouting of sulfur occurs towards sulfonated organic acids.

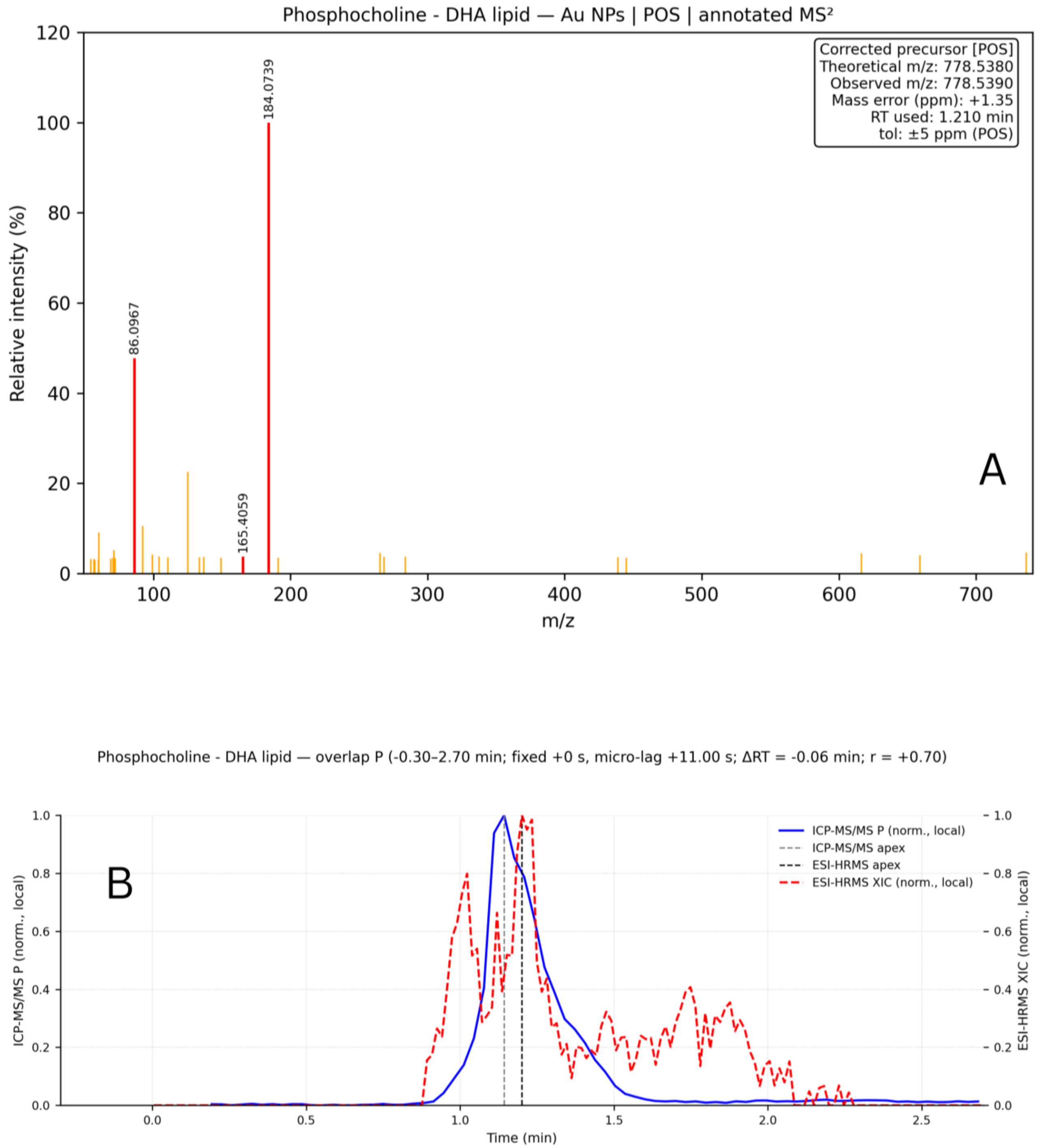


**Figure S29. Phosphocholine–DHA lipid detected in Au NP-treated callus in positive ion mode.** (A) The protonated precursor at m/z 778.5400 (theoretical 778.5380; +2.0 ppm; RT 1.20 min) shows MS² fragments consistent with a phosphocholine headgroup attached to a polyunsaturated (DHA-like) acyl chain. (B) The P ICP-MS/MS signal co-elutes with the HRMS XIC. This species is unique to Au NP-treated callus, supporting phospholipid remodeling under nanoparticle exposure.

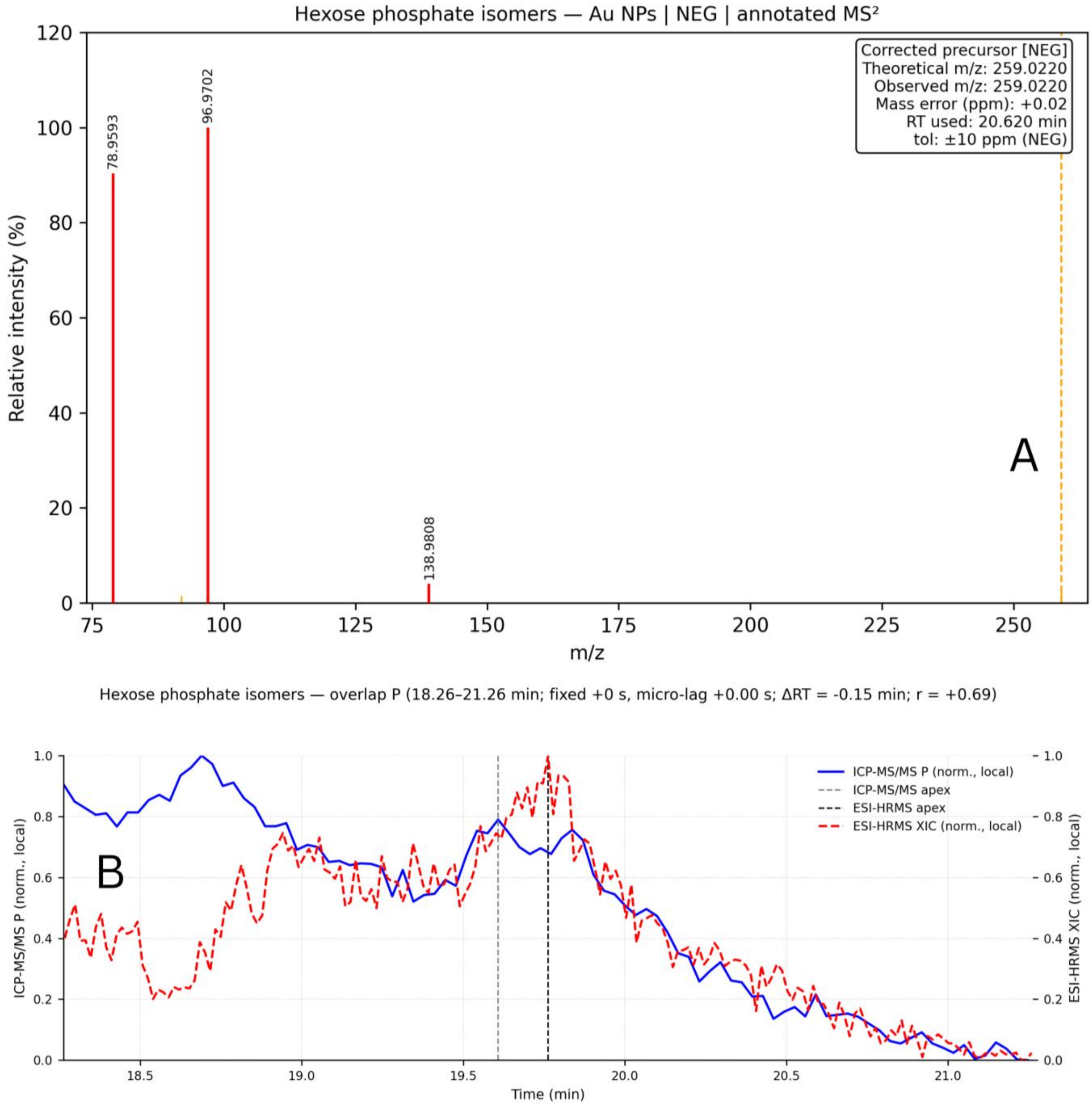


**Figure S30. Hexose phosphate isomers detected in Au NP-treated callus in negative ion mode**. (A) The deprotonated precursor at m/z 259.0220 (theoretical 259.0220; −0.4 ppm; RT 19.78 min) fragments in a manner consistent with phosphorylated hexoses. (B) The P ICP-MS/MS signal overlaps the HRMS XIC, confirming P-containing carbohydrate metabolites. These features are only observed in Au NP-treated callus, suggesting enhanced carbohydrate phosphorylation and flux through hexose-phosphate pools.

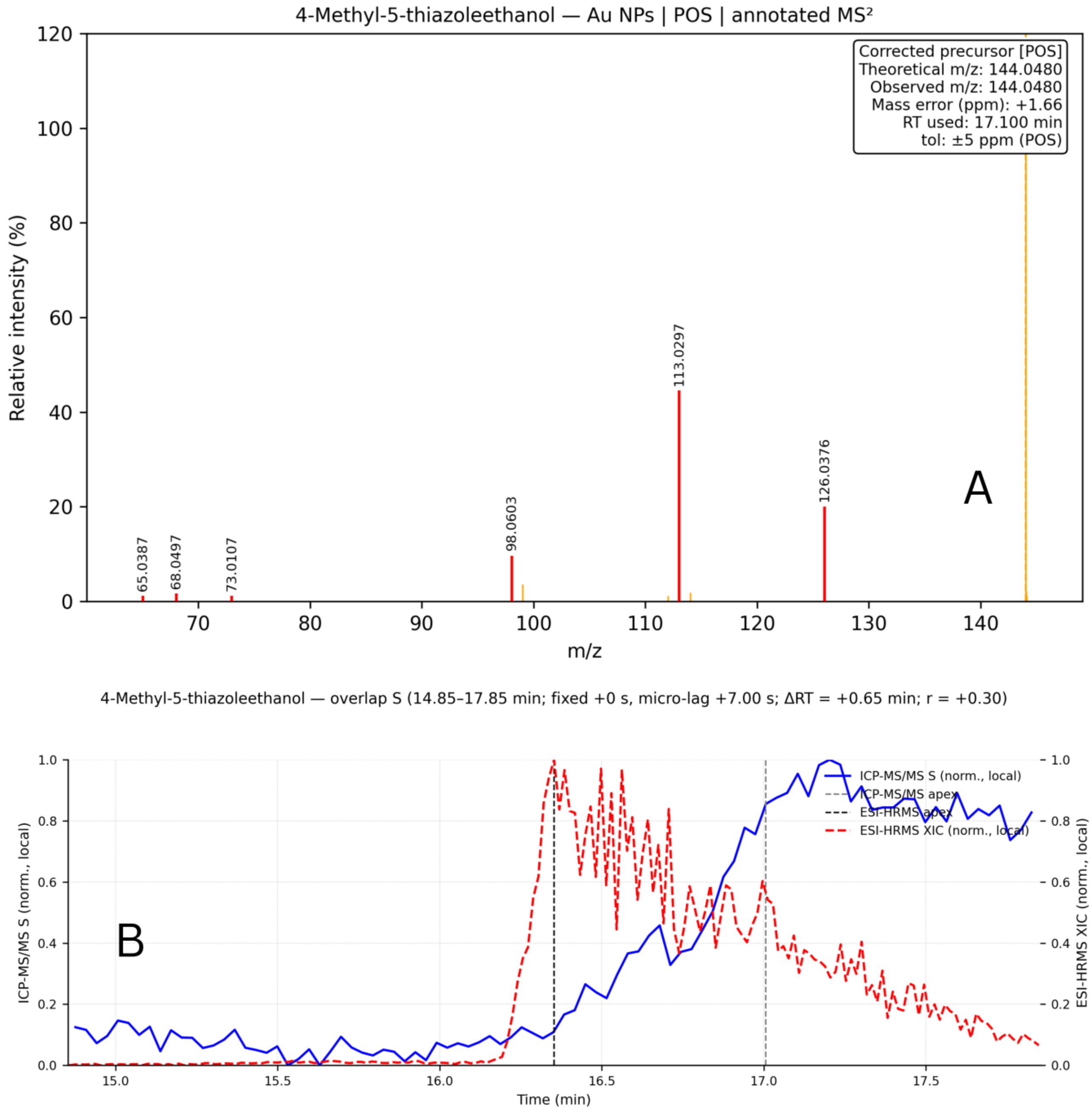


**Figure S31. 4-Methyl-5-thiazoleethanol (thiamine precursor) detected in Au NP-treated callus in positive ion mode.** (A) $MS^2$ confirms the protonated precursor at m/z 144.0480 (theoretical 144.0480; +1.9 ppm; RT 16.35 min), with fragments characteristic of a thiazole ring. (B) Co-elution of the S ICP-MS/MS signal with the HRMS XIC validates the S-containing nature of this intermediate. This is unique to Au NP-treated callus, supporting activation of thiamine biosynthesis and stress-tolerance pathways.

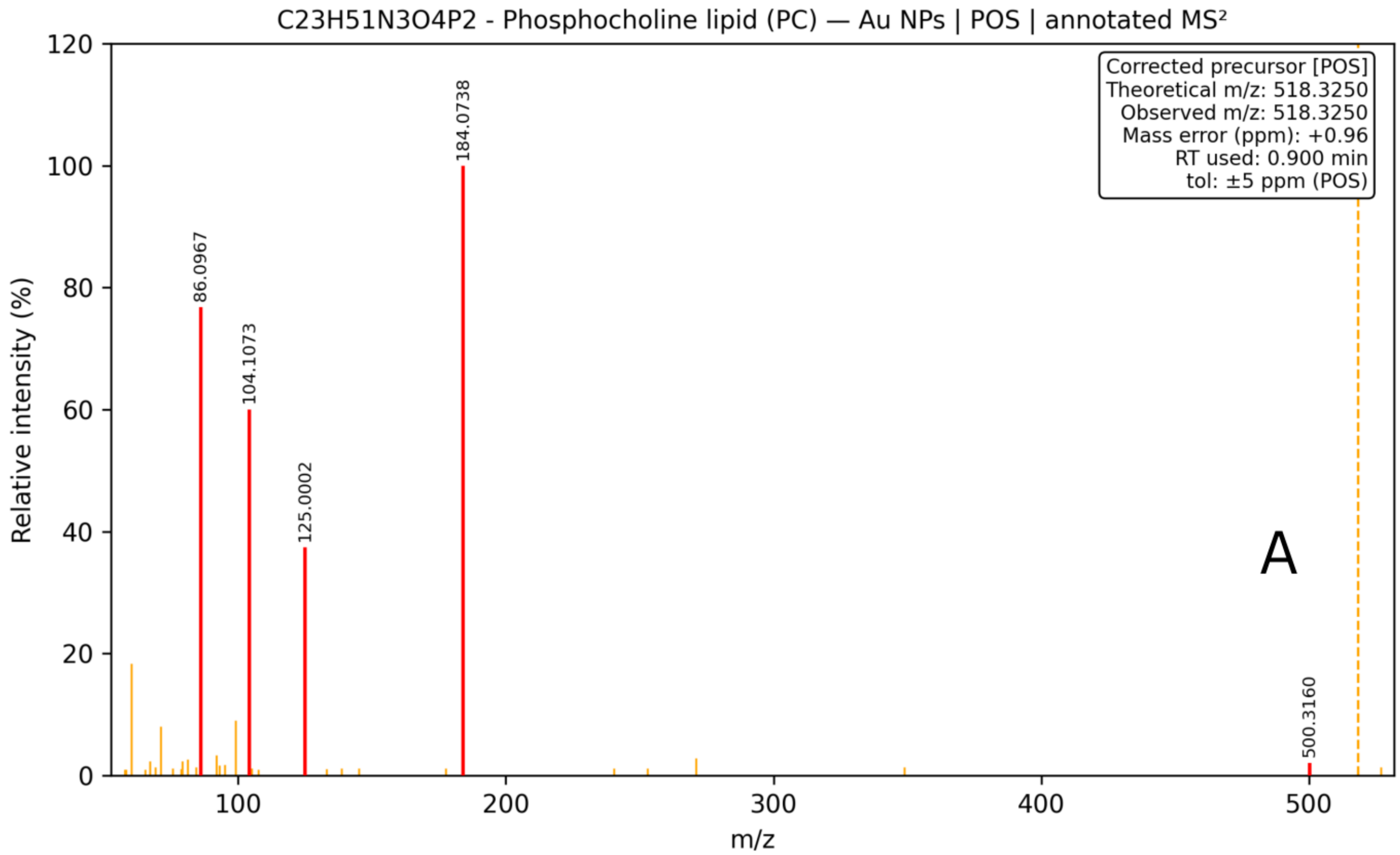


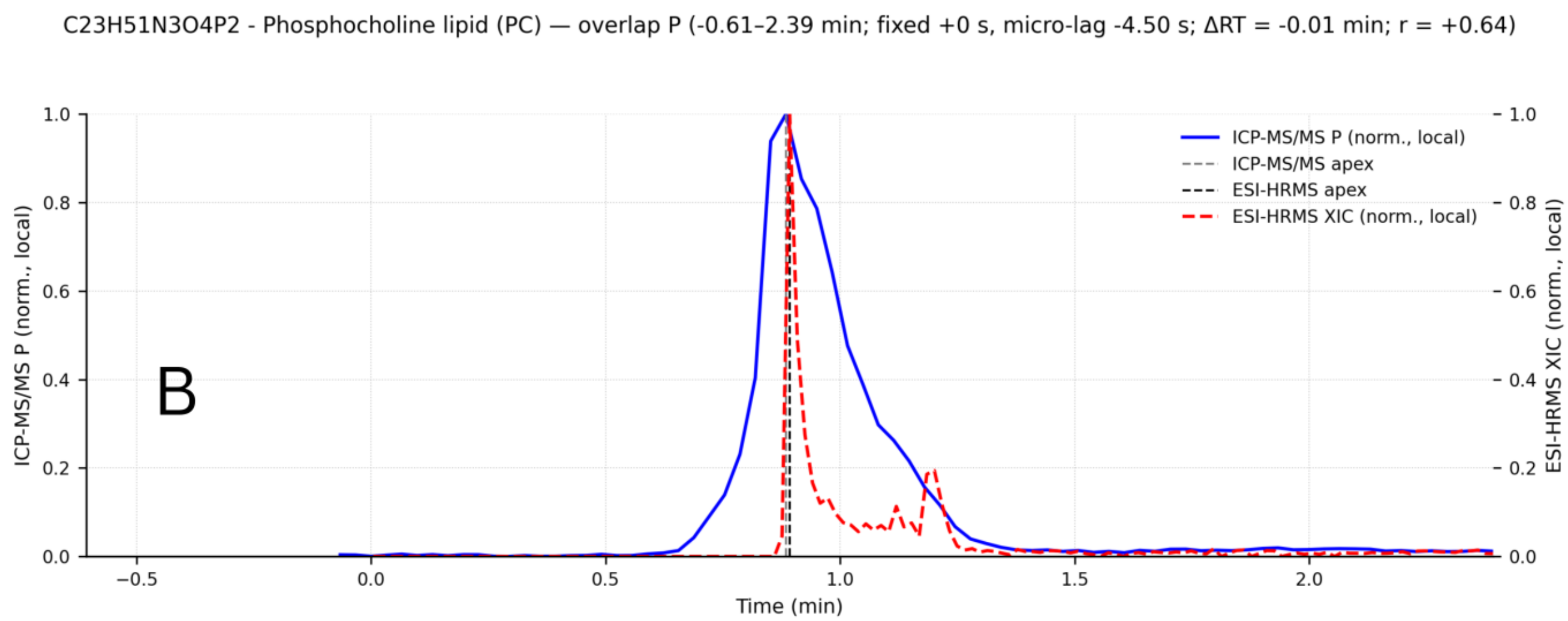


**Figure S32.C23H51N3O4P2 phosphocholine lipid (PC) detected in Au NP-treated callus in positive ion mode.** (A) The sodium adduct $[M+Na]^+$ at m/z 518.3250 (theoretical 518.3250; +1.0 ppm; RT 0.89 min) shows MS² fragments indicative of a phosphocholine lipid backbone. (B) The P ICP-MS/MS signal co-elutes with the HRMS XIC. This PC-like species is unique to Au NP-treated callus, reinforcing phospholipid remodeling as a response to NPs exposure.

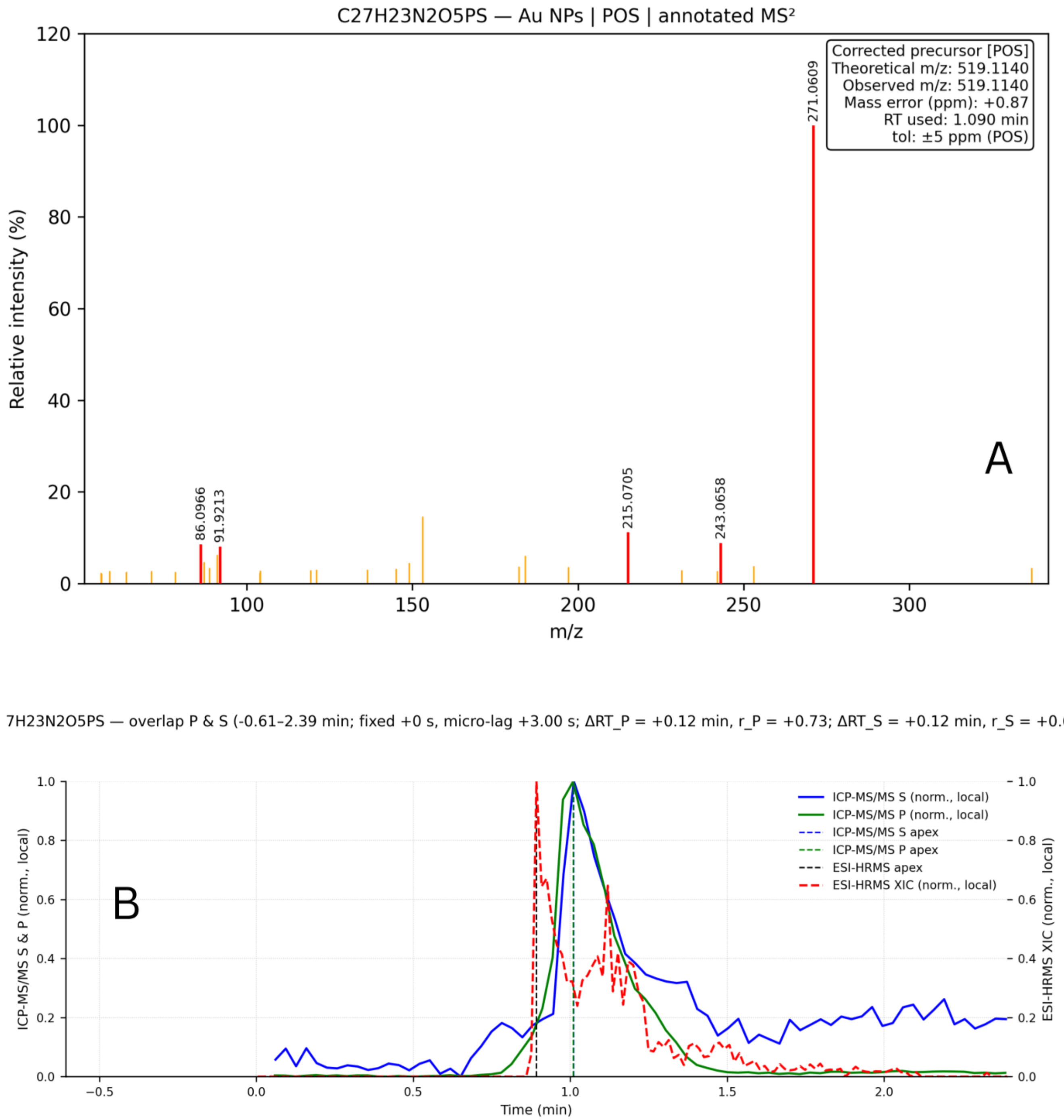


**Figure S33.C27H23N2O5PS detected in Au NP-treated callus in positive ion mode.** (A) The precursor ion at m/z 519.1140 shows a consistent MS² fragmentation pattern across injections. (B) Although both the S and P ICP-MS/MS signals co-eluted with the HRMS XIC signal, this feature was not considered a true endogenous metabolite and showed behavior inconsistent with endogenous compounds. For this reason, it was considered a laboratory contaminant and was excluded from the biological interpretation.

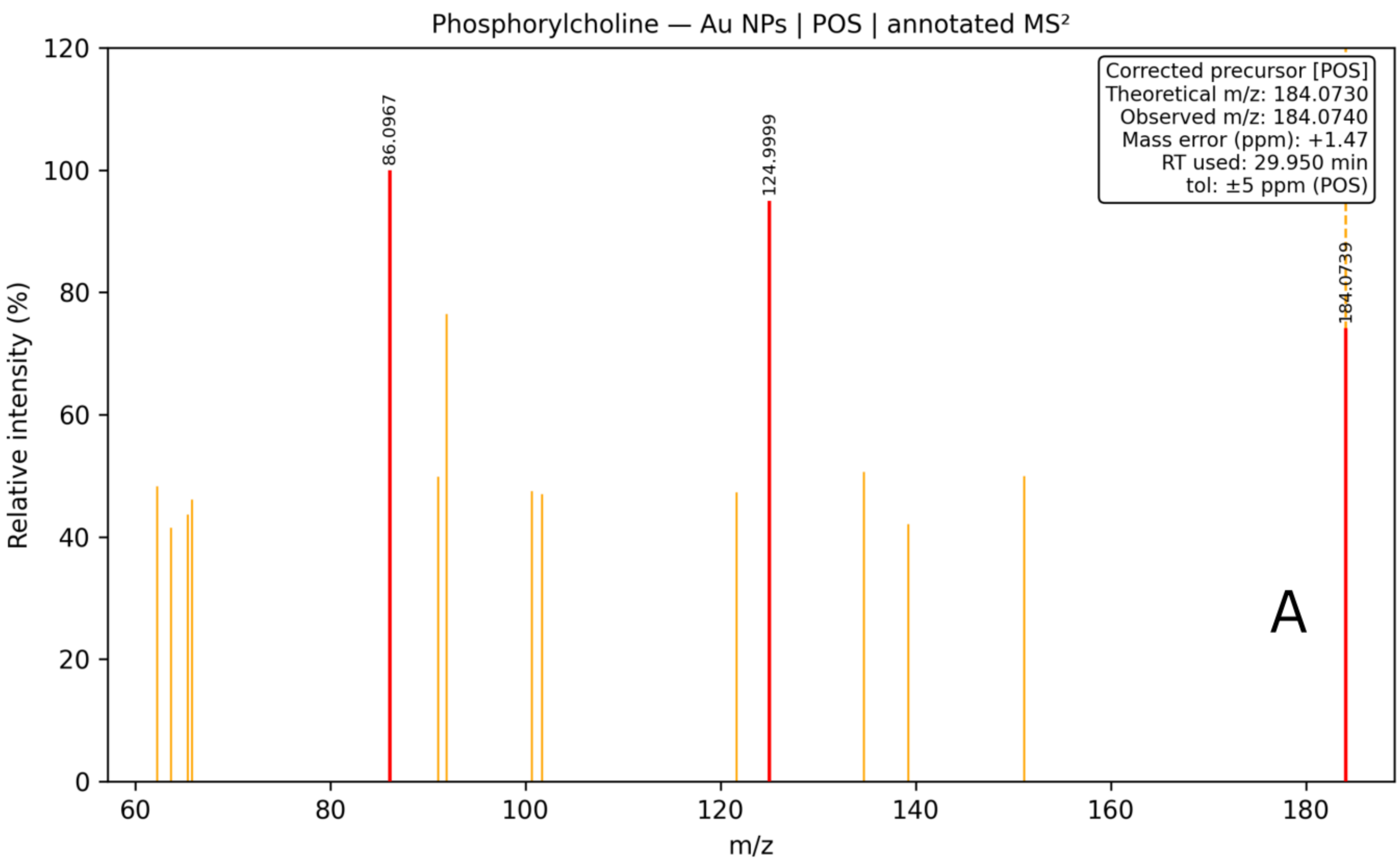


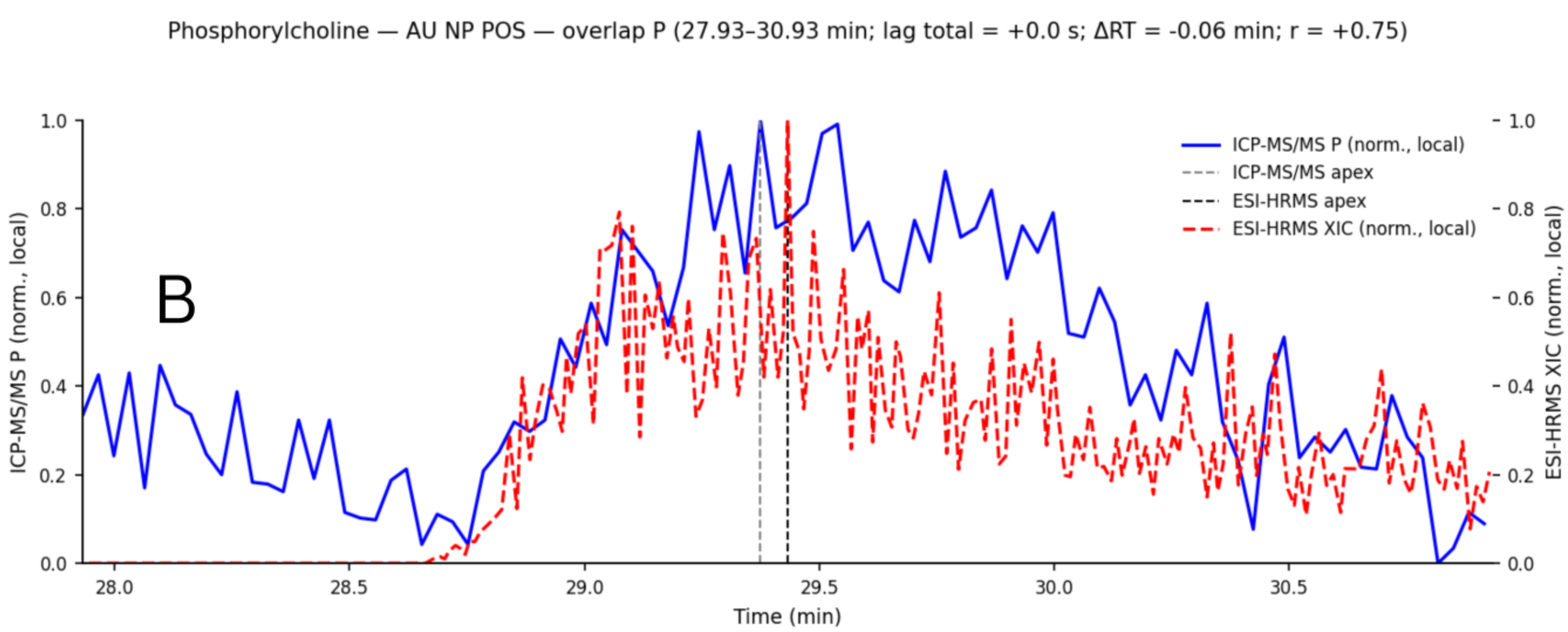


**Figure S34. Phosphorylcholine detected in the Au NP-treated callus in positive ion mode.** (A) The protonated precursor at m/z 184.0740 (theoretical 184.0730; +2.0 ppm; RT 29.43 min) shows the same diagnostic fragments as in control samples. (B) The P ICP-MS/MS signal co-elutes with the HRMS signal. Phosphorylcholine is detected in both groups, but its abundance decreases after Au NP exposure, consistent with phosphocholine consumption and membrane remodeling.

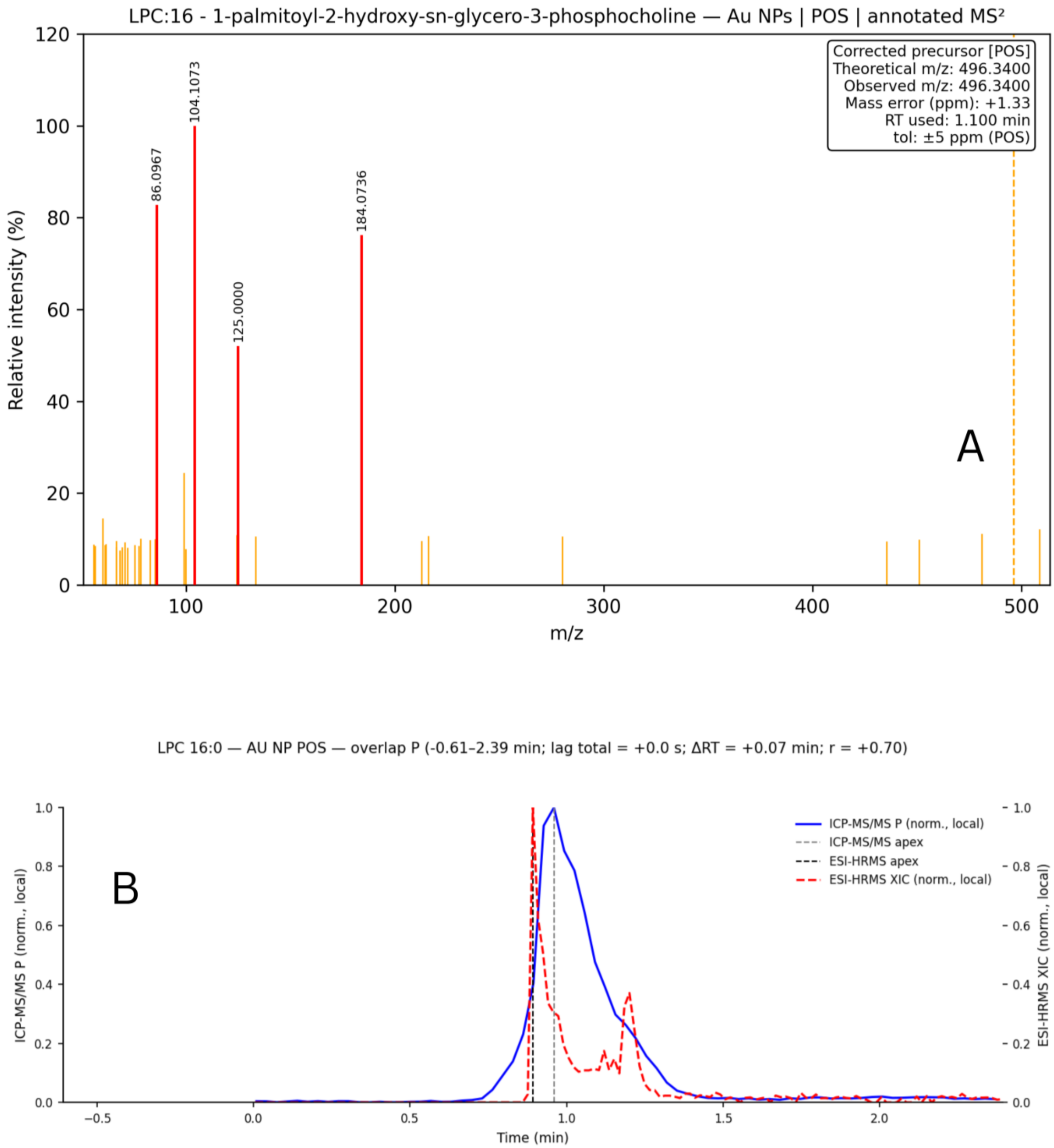


**Figure S35. LPC:16 (1-palmitoyl-2-hydroxy-sn-glycero-3-phosphocholine) detected in Au NP-treated callus in positive ion mode.** (A) The protonated precursor at *m/z* 496.3410 (theoretical 496.3400; +1.9 ppm; RT 1.10 min) shows typical LPC headgroup and acyl-chain fragments. (B) Co-elution of the P ICP-MS/MS signal with the HRMS XIC confirms the P-containing lysophospholipid identity. LPC:16 is present in both groups, but is higher in the Au NP-treated callus, supporting enhanced phospholipid turnover and membrane remodeling.

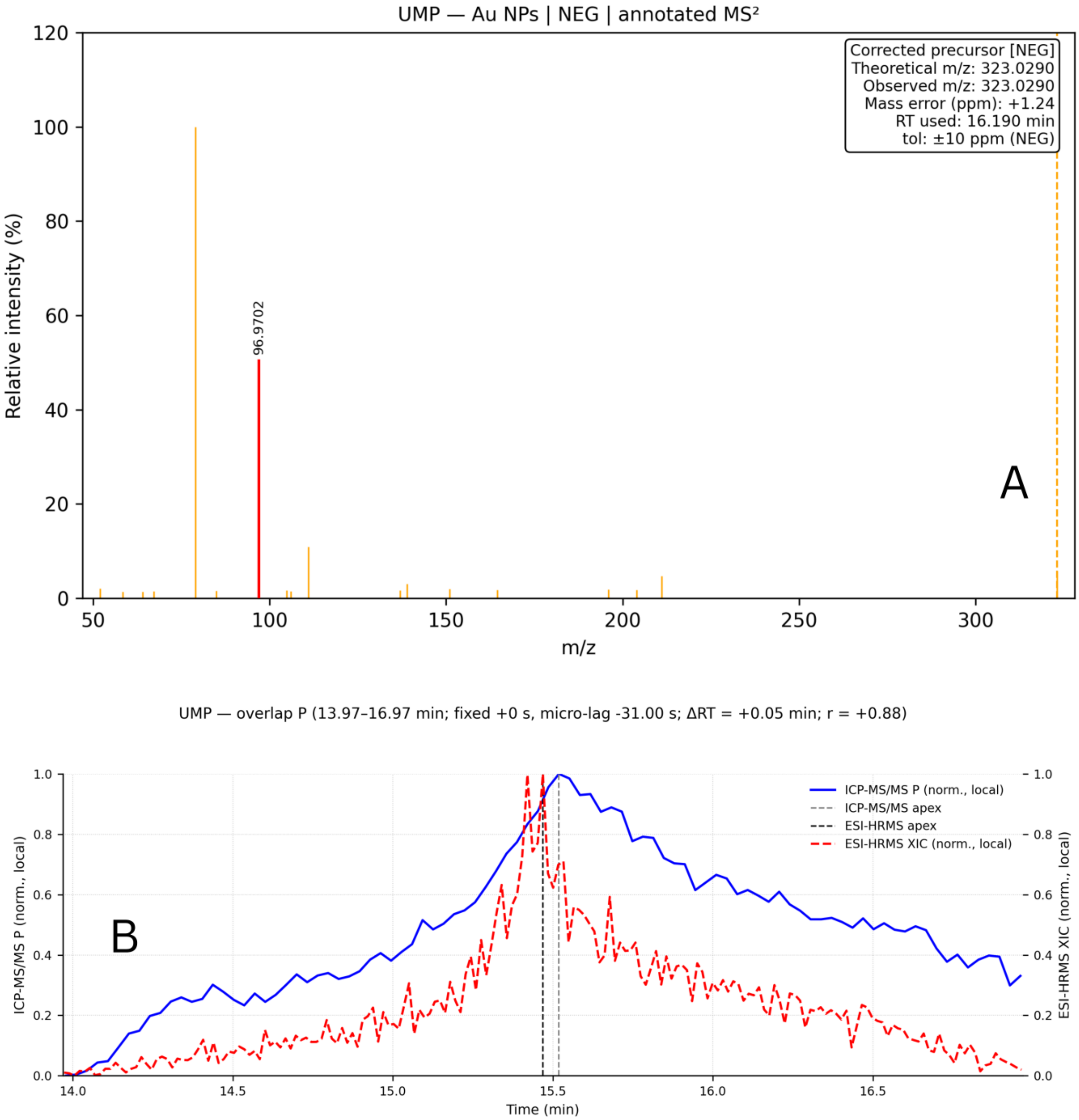


**Figure S36. Uridine monophosphate (UMP) detected in Au NP-treated callus in negative ion mode.** (A) The deprotonated precursor at m/z 323.0300 (theoretical 323.0290; +3.5 ppm; RT 15.45 min) yields nucleotide-typical fragments. (B) The P ICP-MS/MS signal co-elutes with the HRMS XIC. UMP is also observed in controls, indicating that uridylate pools remain detectable under Au NP exposure.

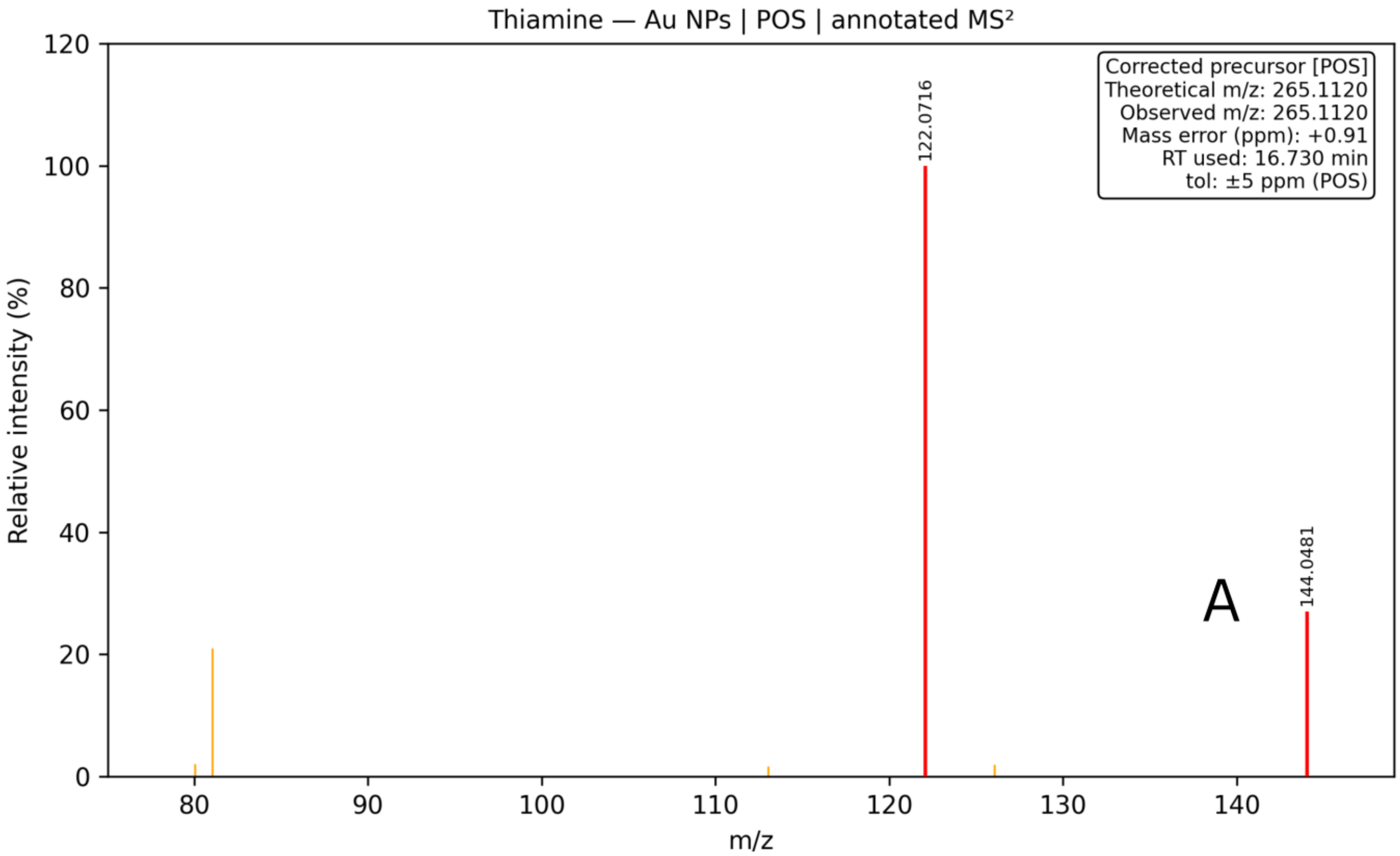


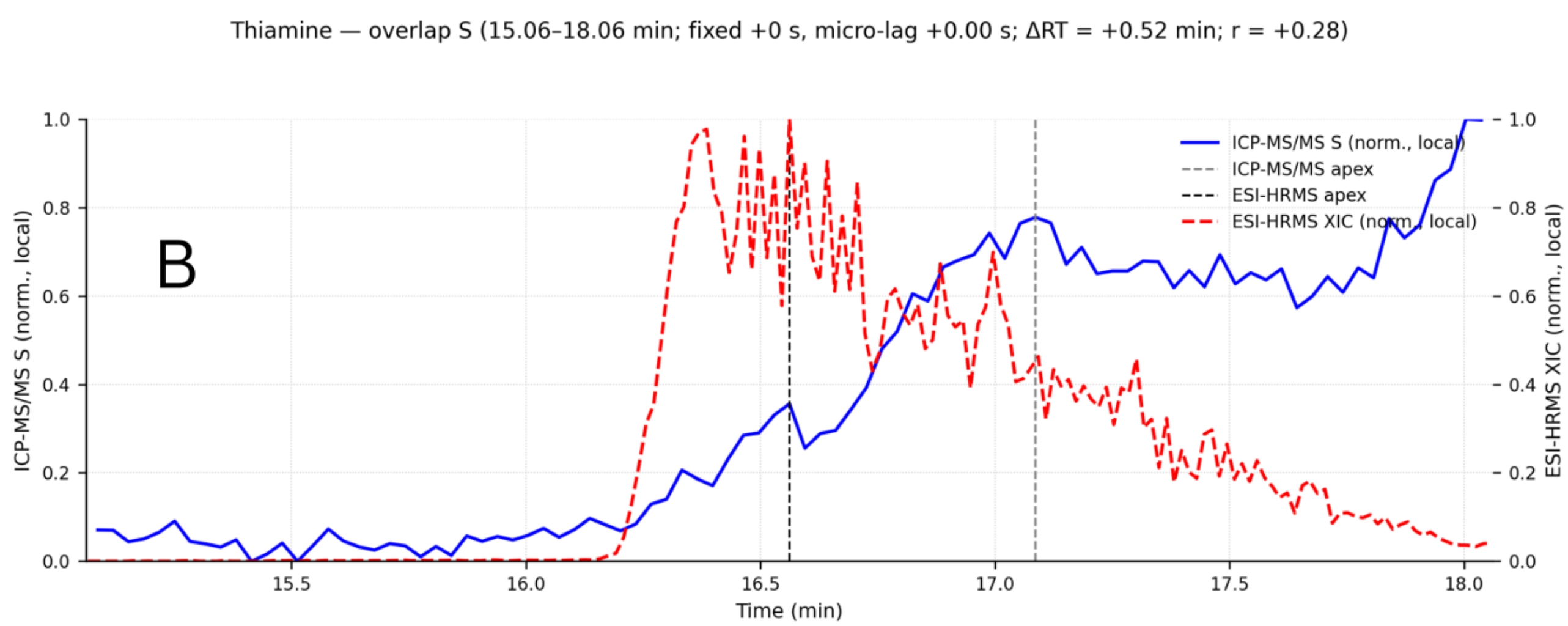


**Figure S37. Thiamine detected in the Au NP-treated callus in positive ion mode.** (A) The protonated precursor at m/z 265.1120 (theoretical 265.1120; +0.6 ppm; RT 16.56 min) shows the same thiazole and aminopyrimidine fragments as in control samples. (B) Co-elution of the S ICP-MS/MS signal with the HRMS XIC confirms the S-containing vitamin. Thiamine is detected in both treatments, with its level increasing in the Au NP-treated callus, supporting the activation of thiamine-linked stress-response pathways.

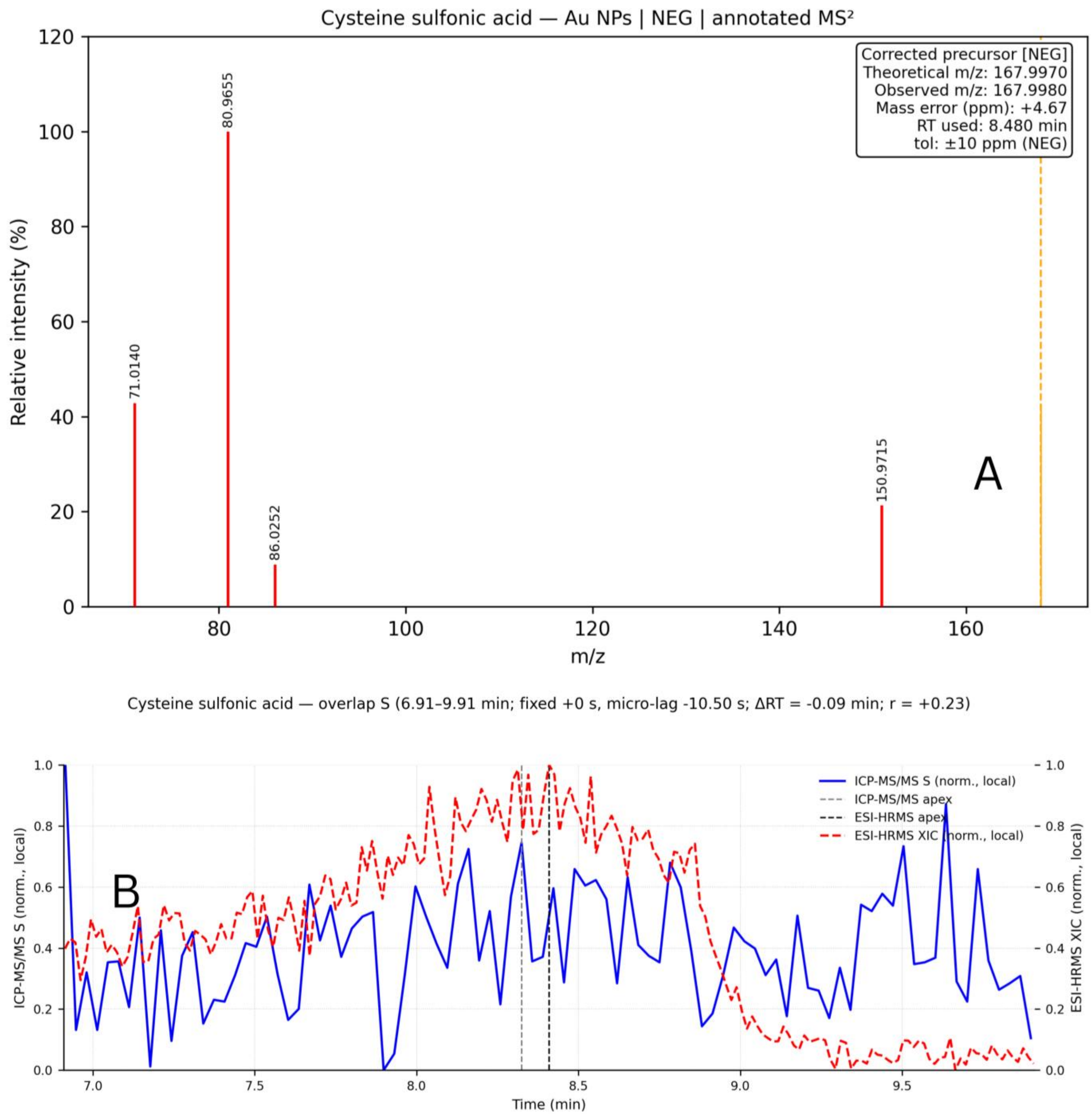


**Figure S38. Cysteine sulfonic acid detected in the Au NP-treated callus in negative ion mode.** (A) MS² confirms the deprotonated precursor at m/z 167.9980 (theoretical 167.9970; +4.9 ppm; RT 8.41 min), with fragment ions characteristic of a fully oxidized cysteine sulfonic group. (B) The S ICP-MS/MS signal co-elutes with the HRMS XIC. This metabolite is unique to Au NP-treated callus and indicates deep oxidation of cysteine and thiol pools.

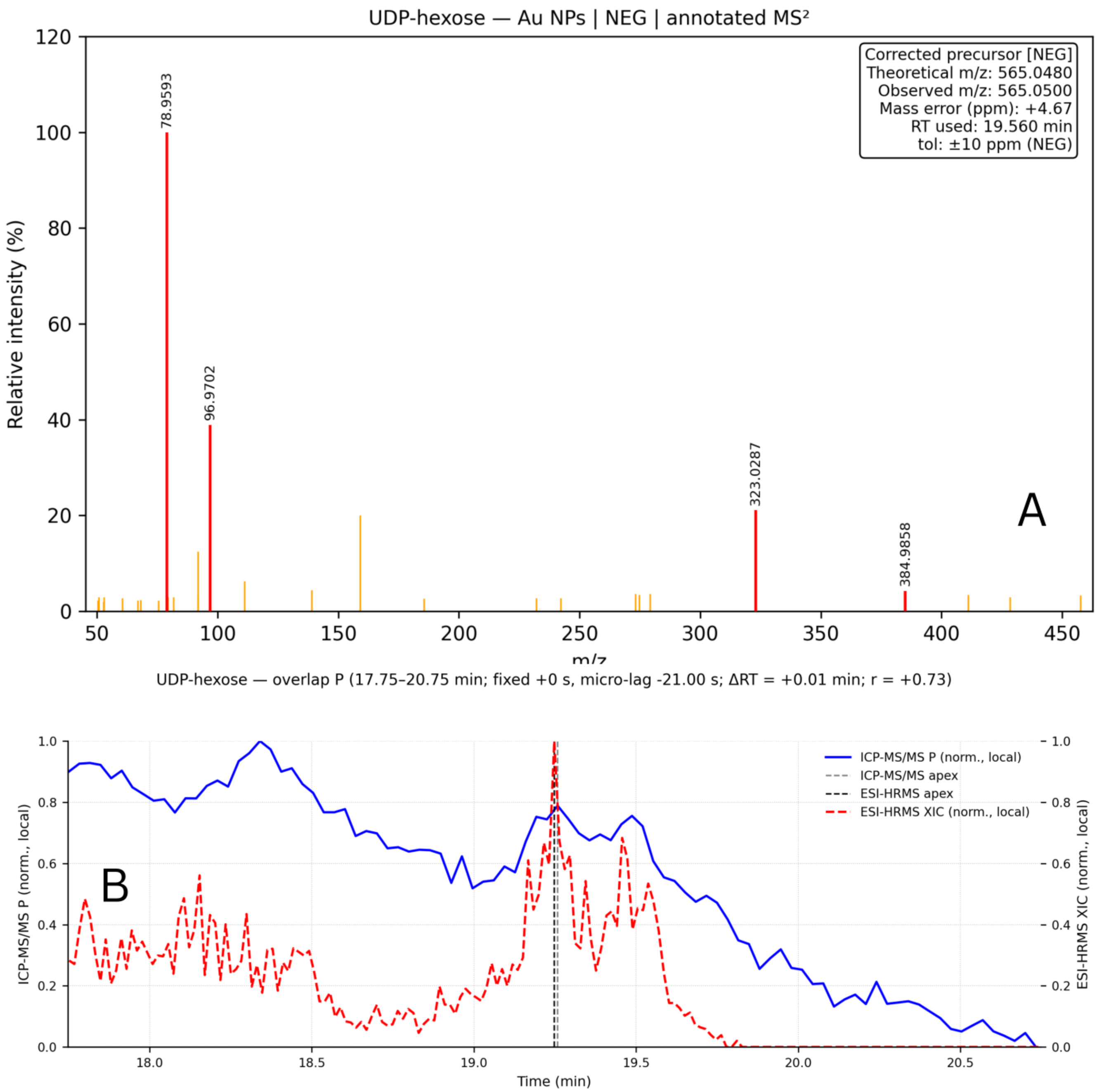


**Figure S39. UDP-hexose detected in Au NP-treated callus in negative ion mode.** (A) The deprotonated precursor at *m/z* 565.0500 (theoretical 565.0480; +4.4 ppm; RT 19.25 min) yields fragments consistent with UDP and hexose moieties. (B) The P ICP-MS/MS signal co-elutes with the HRMS XIC, confirming the presence of a P-rich UDP-sugar. This metabolite is absent in controls, indicating the activation of UDP-sugar metabolism in response to Au NP exposure.

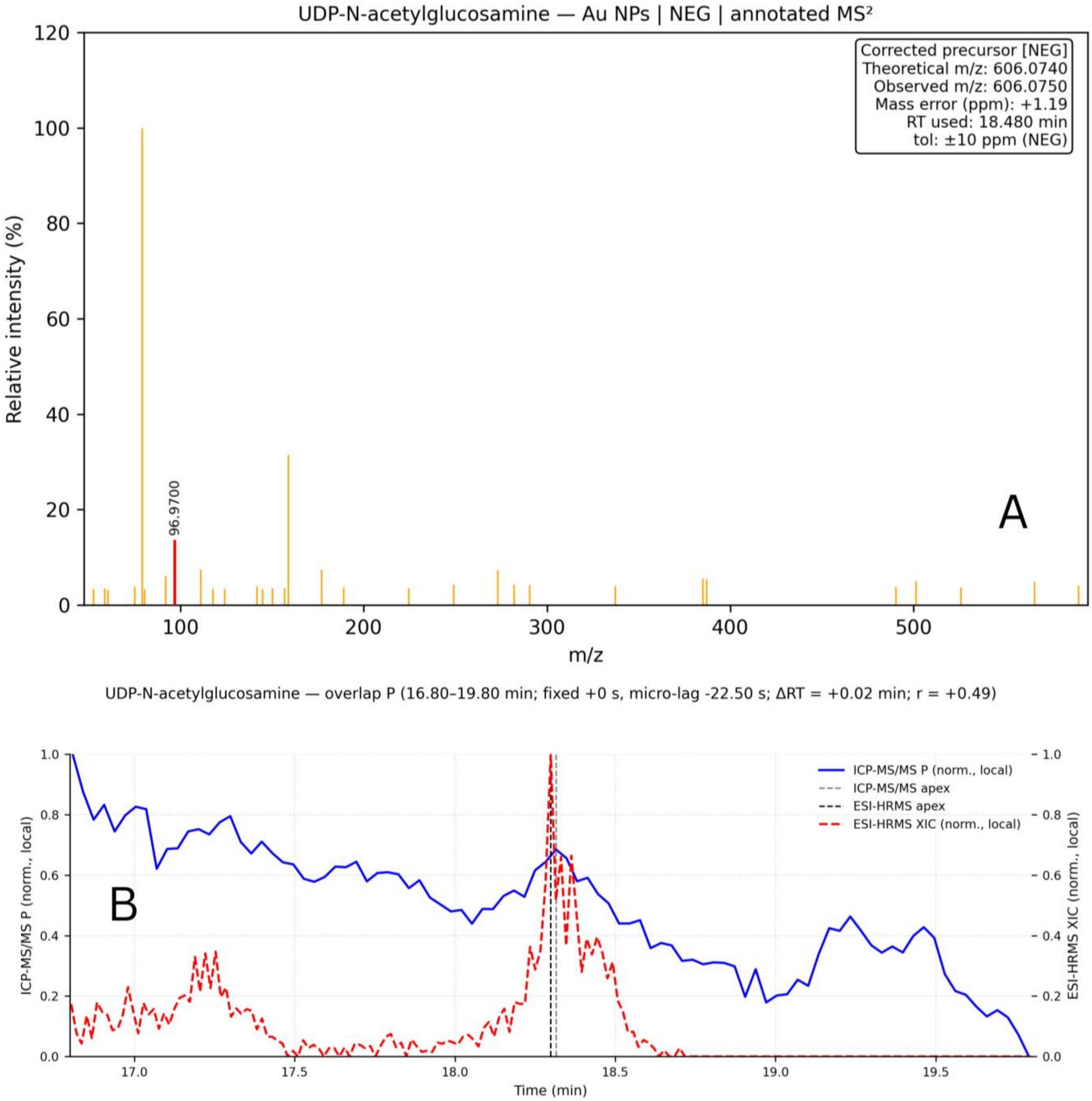


**Figure S40. UDP-N-acetylglucosamine detected in Au NP-treated callus in negative ion mode.** (A) The deprotonated precursor at m/z 606.0750 (theoretical 606.0740; +1.0 ppm; RT 18.30 min) displays MS² fragments characteristic of a UDP-activated N-acetylhexosamine. (B) The P ICP-MS/MS signal overlaps the HRMS XIC. This UDP-sugar is specific to Au NP-treated callus and supports enhanced UDP-sugar pathways linked to cell-wall and glycosylation remodeling.

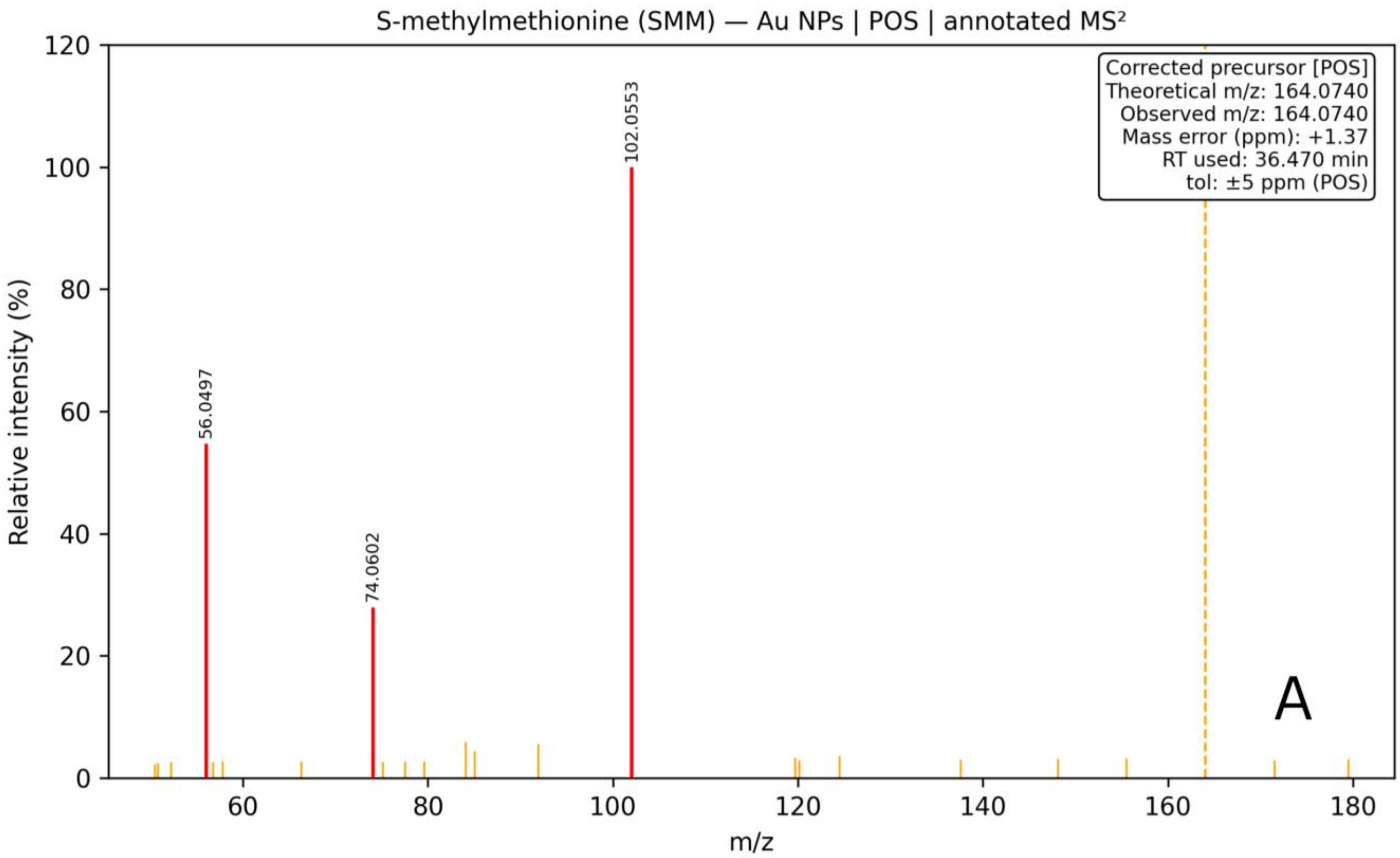


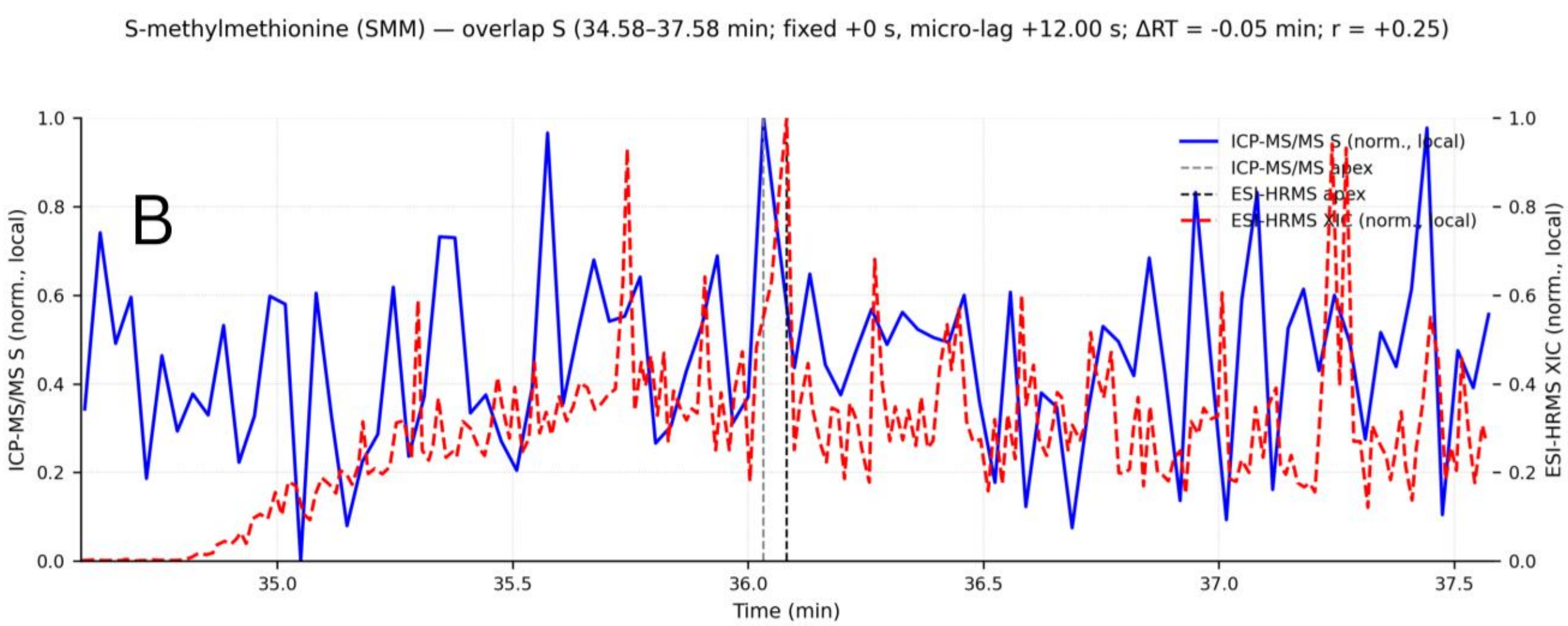


**Figure S41. S-methylmethionine (SMM) detected in Au NP-treated callus in positive ion mode.** (A) The protonated precursor at m/z 164.0740 (theoretical 164.0740; +1.3 ppm; RT 36.08 min) fragments in a way consistent with a methylated methionine derivative. (B) Co-elution of the S ICP-MS/MS signal with the HRMS XIC confirms sulfur content. SMM is unique to Au NP-treated callus and indicates activation of S-methylated sulfur pools and methyl-transfer buffering under NP stress.

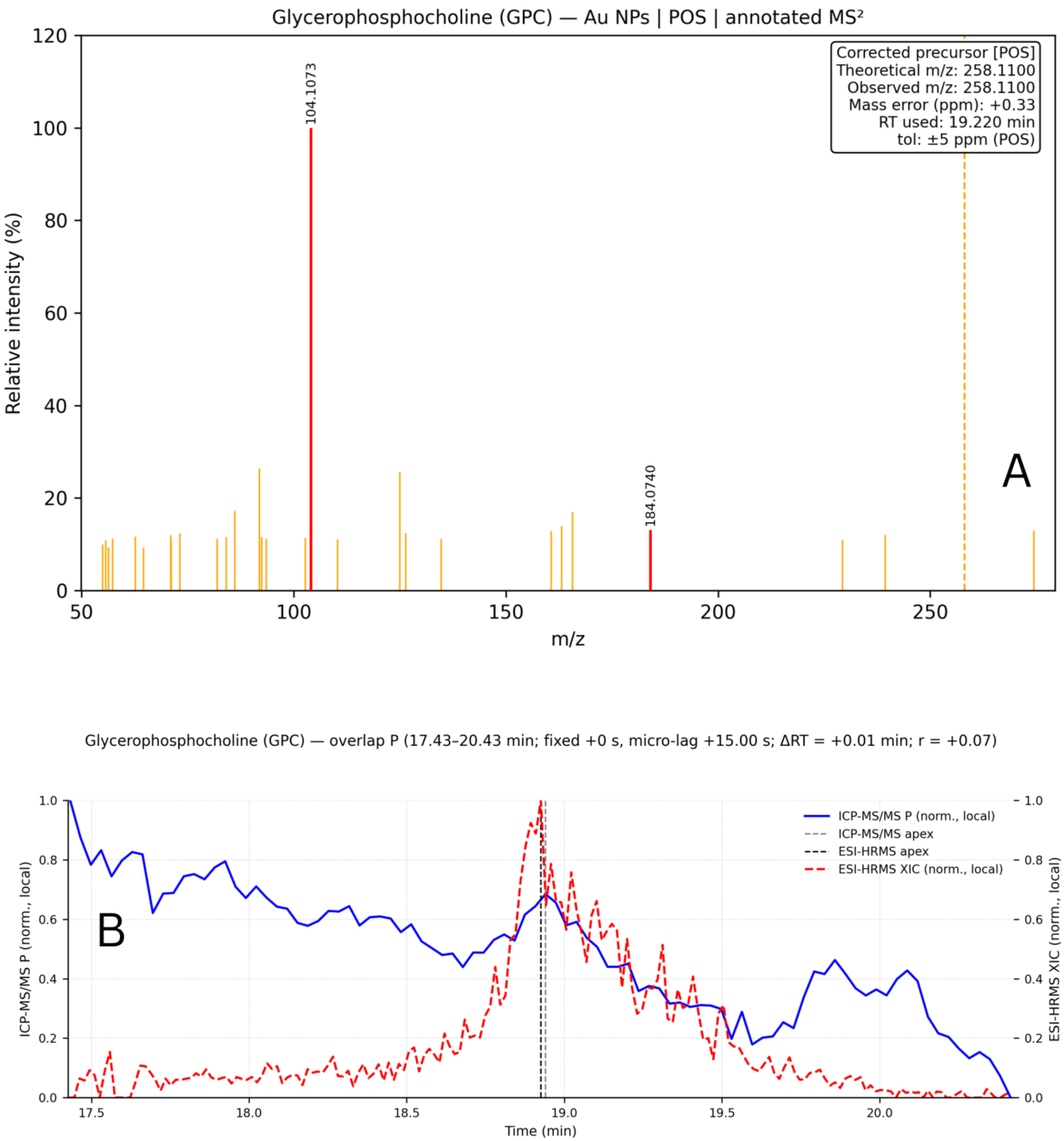


**Figure S42. Glycerophosphocholine (GPC) detected in Au NP-treated callus in positive ion mode.** (A) MS² confirms the protonated precursor at *m/z* 258.1090 (theoretical 258.1100; −2.9 ppm; RT 18.93 min), with fragments typical of a glycerophosphocholine backbone. (B) The P ICP-MS/MS signal co-elutes with the HRMS XIC. GPC appears only in Au NP-treated callus, supporting enhanced phosphocholine turnover and lysophospholipid recycling.

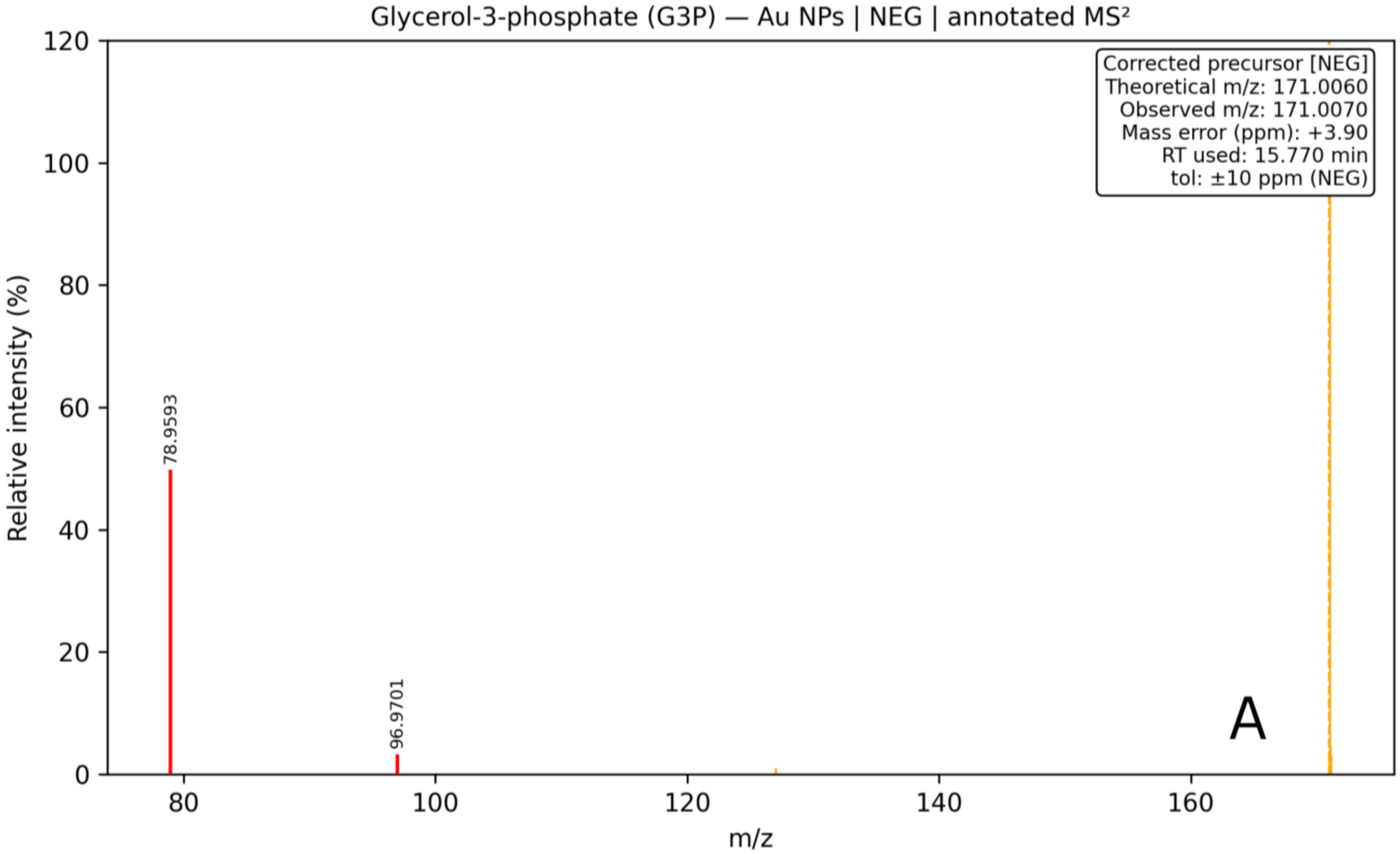


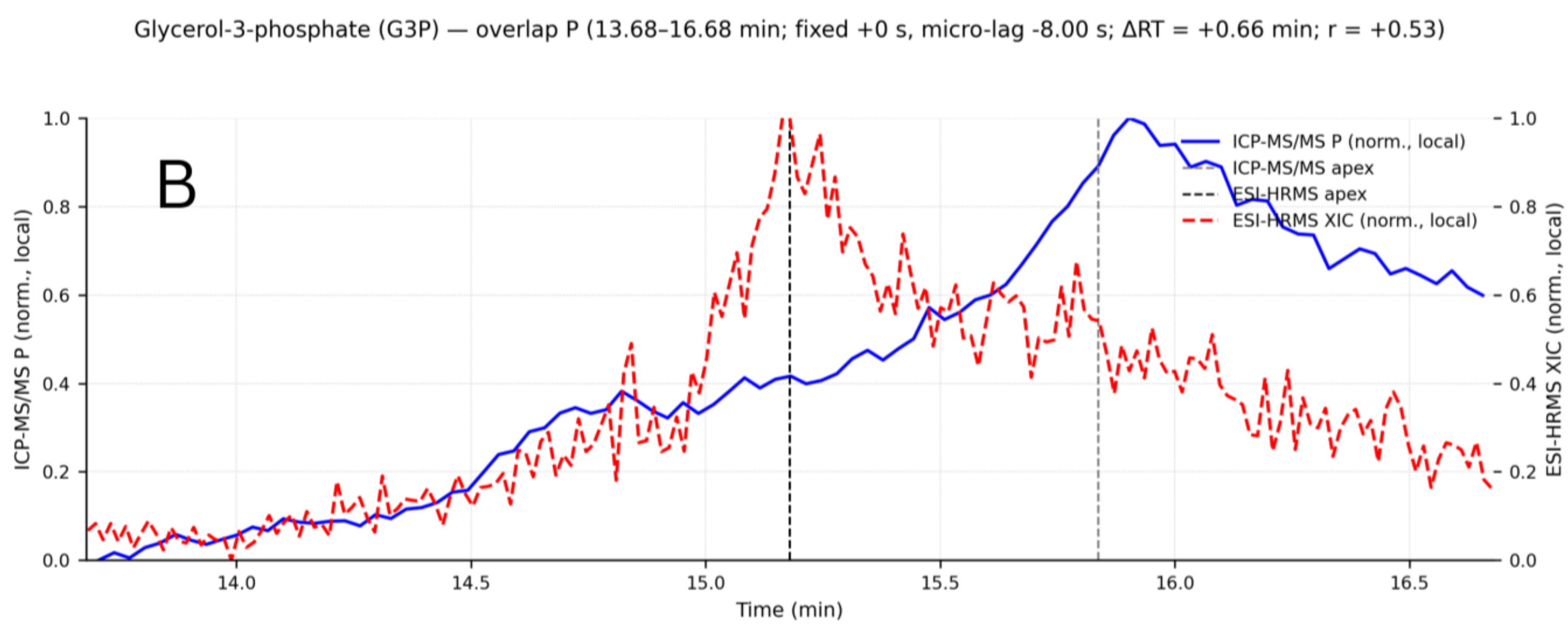


**Figure S43. Glycerol-3-phosphate (G3P) detected in Au NP-treated callus in negative ion mode.** (A) The deprotonated precursor at m/z 171.0070 (theoretical 171.0060; +4.7 ppm; RT 15.18 min) shows the same diagnostic fragments as in the control sample. (B) Co-elution of the P ICP-MS/MS trace with the HRMS XIC confirms a P-containing glycerol phosphate. Detection in both control and Au NP-treated callus indicates that this phosphorylated intermediate remains part of core central-carbon metabolism.

## 3. DISCUSSION

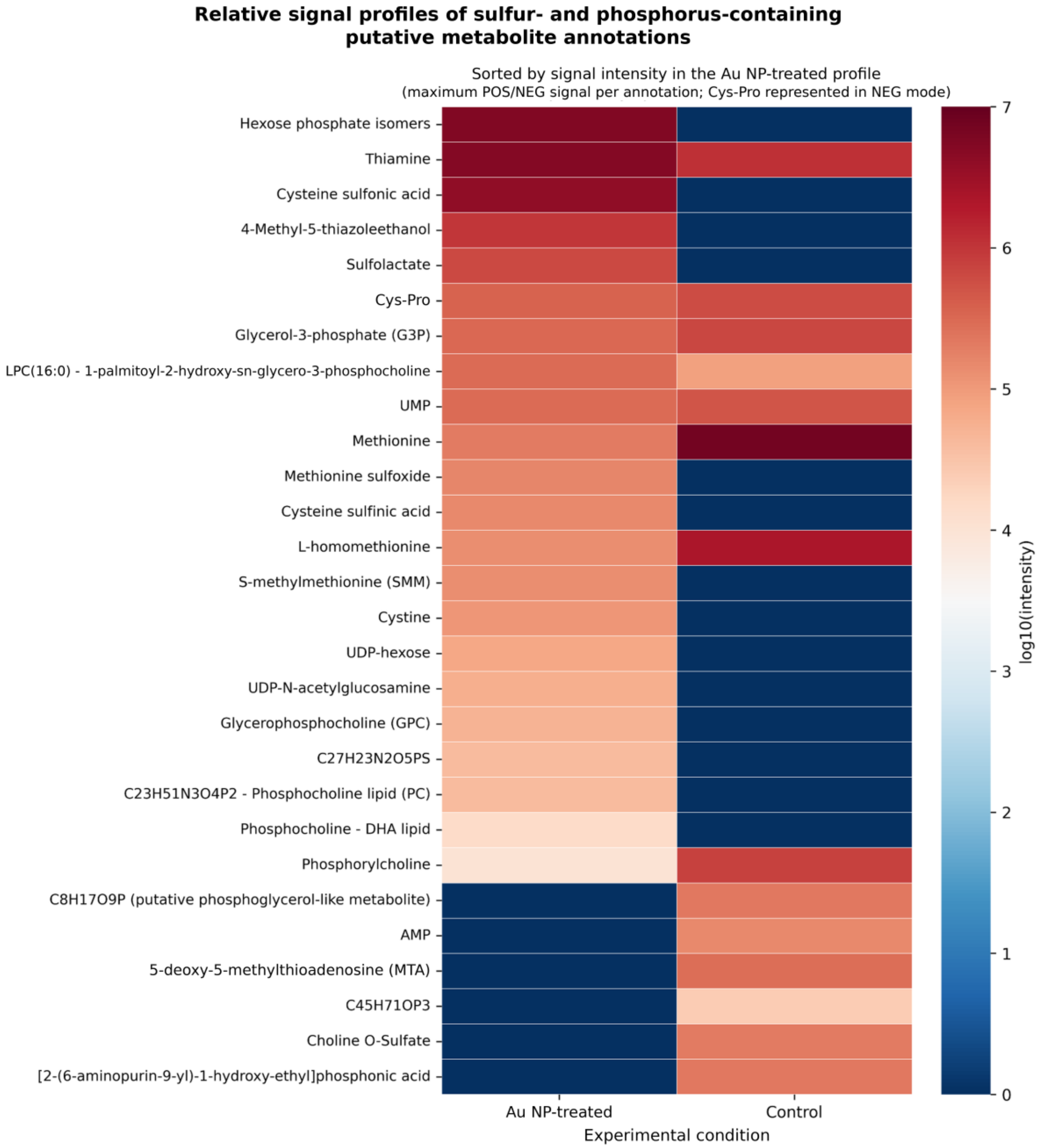


**Figure S44**. Heatmap of the relative LC–HRMS signal intensities ($\log_{10}$-transformed peak areas) of the retained P- and S-containing putative metabolite annotations in the control and Au NP-treated analytical profiles. For annotations detected in both ionization modes, the maximum signal between positive and negative modes was used; Cys-Pro was represented using the negative-ion signal. Blue indicates lower relative signal intensity, and red indicates higher relative signal intensity.

**Table S9.** Relative LC–HRMS signals and descriptive $log_2$ signal ratios for the 28 retained P- and/or S-containing putative metabolite annotations in the control and Au NP-treated profiles.

| Putative metabolite annotation | Descriptive $log_2$ signal ratio (Au NP-treated/control) | Control relative LC–HRMS signal | Au NP-treated relative LC–HRMS signal |
|---|---|---|---|
| Hexose phosphate isomers | --- | ND | 5573041 |
| Cysteine sulfonic acid | --- | ND | 4052256 |
| 4-Methyl-5-thiazoleethanol | --- | ND | 988245 |
| Sulfolactate | --- | ND | 634198 |
| Methionine sulfoxide | --- | ND | 162780 |
| Cysteine sulfinic acid | --- | ND | 148425 |
| S-methylmethionine (SMM) | --- | ND | 134381 |
| Cystine | --- | ND | 113974 |
| UDP-hexose | --- | ND | 70892 |
| UDP-N-acetylglucosamine | --- | ND | 59699 |
| Glycerophosphocholine (GPC) | --- | ND | 53089 |
| C27H23N2O5PS | --- | ND | 41533 |
| C23H51N3O4P2 - Phosphocholine lipid (PC) | --- | ND | 40839 |
| Phosphocholine - DHA lipid | --- | ND | 14894 |
| Thiamine | 2.126263605 | 1153307 | 5035169 |
| LPC:16 - 1-palmitoyl-2-hydroxy-sn-glycero-3-phosphocholine | 1.818315043 | 87746 | 309453 |
| UMP | -0.722572118 | 510635 | 309453 |
| Cys-pro | -0.767503154 | 621211 | 364920 |
| Glycerol-3-phosphate (G3P) | -1.02270261 | 677829 | 333623 |
| L-homomethionine | -4.104683536 | 2311909 | 134381 |
| Methionine | -5.202493353 | 7593308 | 206217 |
| Phosphorylcholine | -6.211546757 | 765530 | 10330 |
| C45H71OP3 | --- | 24397 | 0 |
| AMP | --- | 149533 | 0 |
| Choline O-Sulfate | --- | 203926 | 0 |
| [2-(6-aminopurin-9-yl)-1-hydroxy-ethyl]phosphonic acid | --- | 215656 | 0 |
| C8H17O9P (putative phosphoglycerol-like metabolite) | --- | 222390 | 0 |
| 5-deoxy-5-methylthioadenosina (MTA) | --- | 292309 | 0 |

ND, not detected above the workflow detection and filtering criteria. $Log_2$ signal ratios were calculated only for putative annotations detected in both analytical profiles and are presented descriptively. No inferential

statistical analysis or FDR correction was applied because the comparison comprised one processed pooled analytical profile per experimental condition.

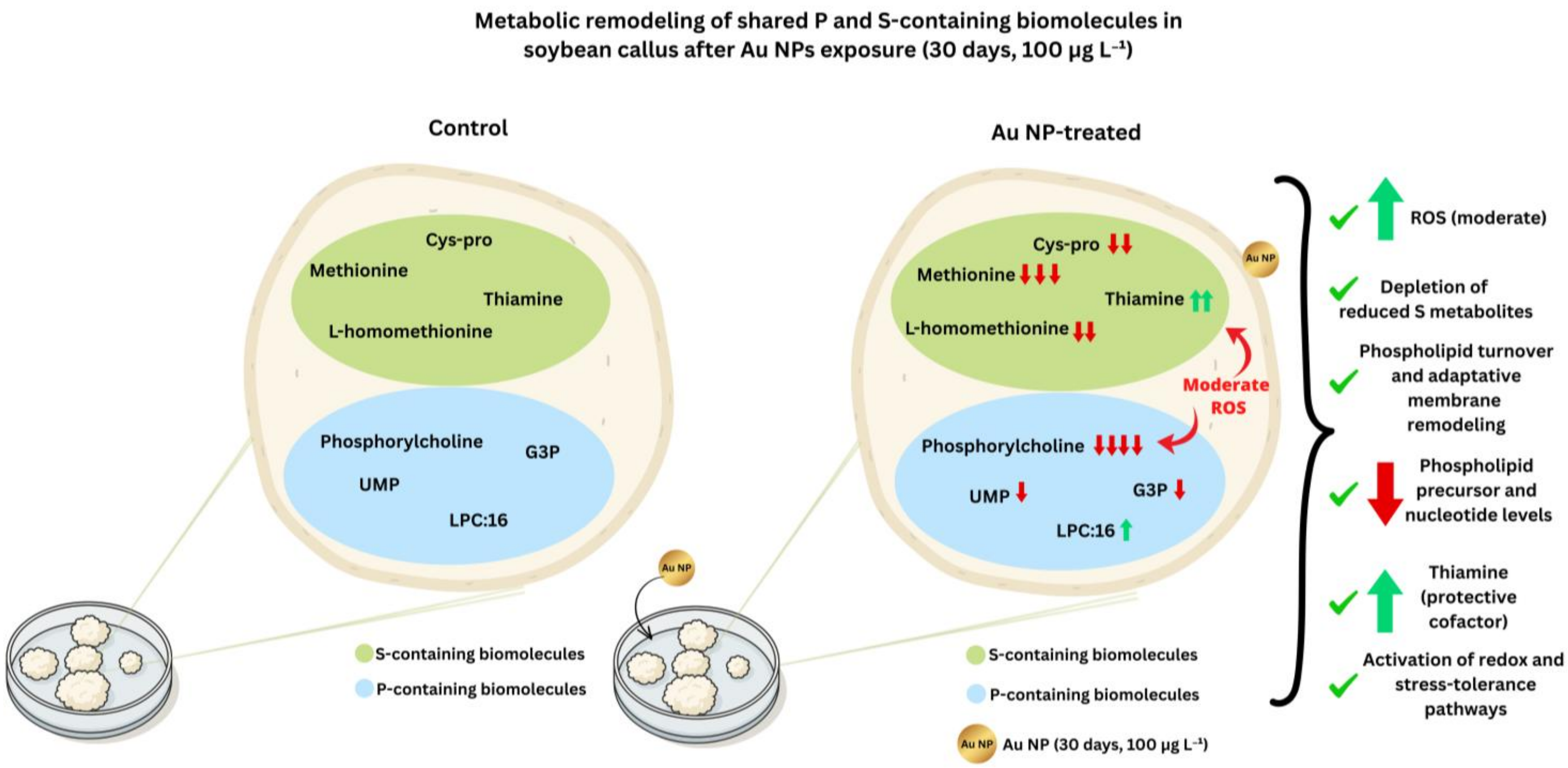


**Figure S45.** Schematic comparison of the relative LC–HRMS signal patterns of shared S- and P-containing putative metabolite annotations in the untreated and Au NP-treated soybean callus profiles. Red and green arrows indicate, respectively, lower and higher relative signals in the Au NP-treated analytical profile compared with the untreated control. These relative signal differences were analytically observed. The pathway-level relationships depicted, including ROS-related responses, membrane effects, phospholipase activity, stress tolerance, and metabolic adaptation, are literature-informed mechanistic hypotheses proposed to contextualize the observed chemical profile; these processes were not directly measured and should not be interpreted as evidence of pathway activation or causality.

**Table S10**. Selected putative metabolite annotations detected only in the Au NP-treated analytical profile and their reported biochemical associations.

| Metabolite | Biochemical class | Primary functions in plants | Possible metabolic association | Supporting references |
|---|---|---|---|---|

| | | | | |
|---|---|---|---|---|
| **4-Methyl-5-thiazoleethanol** | Thiamine (vitamin B1) thiazole-pathway intermediate | Precursor of TPP; redox and energy metabolism; induced by oxidative and metal stress | Its condition-specific detection is consistent with a difference in thiamine-associated metabolism; it does not establish metabolic priming or enhanced stress tolerance | [9–14] |
| **S-Methylmethionine (SMM)** | Sulfur amino acid derivative | Membrane stabilizer; methyl-donor reservoir; regulator of S-homeostasis; precursor in methionine cycles | Its condition-specific detection is consistent with a difference in sulfur and methyl-group metabolism; it does not demonstrate improved stress resilience | [15–21] |
| **PC-like / DHA-like phospholipids & GPC** | Bioactive membrane phospholipids | Modulate membrane fluidity; substrates for phospholipases; precursors of signaling lipids; maintain membrane structure | Their condition-specific detection is consistent with differences in phospholipid-related metabolism; we did not directly measure phospholipase activation or membrane adaptation. | [22–27] |

## Correspondence and Data Access

The computational workflows and source code used in this study have been deposited in Zenodo (DOI: 10.5281/zenodo.20529690). For further information regarding the workflow implementation, data processing procedures, or access to additional materials, please

contact the corresponding author. Processed datasets supporting the findings of this study are provided in the Supporting Information. Raw datasets will be deposited in a public repository upon manuscript acceptance.

REFERENCES


(1) Kato, L. S.; Cerqueira da Silva, V. H.; Campaci de Andrade, D.; Cruz, G.; Pedrobom, J. H.; Raab, A.; Feldmann, J.; Arruda, M. A. Z. Multimodal Chemical Speciation Techniques Based on Simultaneous High Resolution Molecular/Atomic Mass Spectrometry Applied to Online Target/Non-Target Analysis: A Tutorial Review. *Anal. Chim. Acta* **2024**, 343084. https://doi.org/https://doi.org/10.1016/j.aca.2024.343084.

(2) Lange, E.; Tautenhahn, R.; Neumann, S.; Gröpl, C. Critical Assessment of Alignment Procedures for LC-MS Proteomics and Metabolomics Measurements. *BMC Bioinformatics* **2008**, *9* (1), 375. https://doi.org/10.1186/1471-2105-9-375.

(3) Sacks, G. L.; Derry, L. A.; Brenna, J. T. Elemental Speciation by Parallel Elemental and Molecular Mass Spectrometry and Peak Profile Matching. *Anal. Chem.* **2006**, *78* (24), 8445–8455. https://doi.org/10.1021/ac0612170.

(4) Mattusch, J.; Moeller, D.; Gonzalez, M. P. E.; Wennrich, R. Investigation of the Degradation of Arsenobetaine during Its Contact with Natural Zeolites and the Identification of Metabolites Using HPLC Coupled with ICP-MS and ESI-MS. *Anal. Bioanal. Chem.* **2008**, *390* (7), 1707–1715. https://doi.org/10.1007/s00216-008-1881-7.

(5) de Vlieger, J. S. B.; Giezen, M. J. N.; Falck, D.; Tump, C.; van Heuveln, F.; Giera, M.; Kool, J.; Lingeman, H.; Wieling, J.; Honing, M.; Irth, H.; Niessen, W. M. A. High Temperature Liquid Chromatography Hyphenated with ESI-MS and ICP-MS Detection for the Structural Characterization and Quantification of Halogen Containing Drug Metabolites. *Anal. Chim. Acta* **2011**, *698* (1), 69–76. https://doi.org/https://doi.org/10.1016/j.aca.2011.04.053.

(6) Feldmann, J.; Hansen, H. R.; Karlsson, T. M.; Christensen, J. H. ICP-MS As a Contributing Tool to Nontarget Screening (NTS) Analysis for Environmental Monitoring. *Environ. Sci. Technol.* **2024**, *58* (29), 12755–12762. https://doi.org/10.1021/acs.est.4c00504.

(7) Michalke, B. Review about Powerful Combinations of Advanced and Hyphenated Sample Introduction Techniques with Inductively Coupled Plasma-Mass Spectrometry (ICP-MS) for Elucidating Trace Element Species in Pathologic Conditions on a Molecular Level. *Int. J. Mol. Sci.* **2022**, *23* (11). https://doi.org/10.3390/ijms23116109.

(8) Seiko Kato, L. Multimodal LC–HRMS × ICP–MS/MS Data Processing

Workflows (Scripts 1–3 and $MS^2$ Batch Validation). Zenodo June 2026. https://doi.org/10.5281/zenodo.20529691.

(9) Tunc-Ozdemir, M.; Miller, G.; Song, L.; Kim, J.; Sodek, A.; Koussevitzky, S.; Misra, A. N.; Mittler, R.; Shintani, D. Thiamin Confers Enhanced Tolerance to Oxidative Stress in Arabidopsis. *Plant Physiol.* **2009**, *151* (1), 421–432. https://doi.org/10.1104/pp.109.140046.

(10) Chatterjee, A.; Jurgenson, C. T.; Schroeder, F. C.; Ealick, S. E.; Begley, T. P. Biosynthesis of Thiamin Thiazole in Eukaryotes: Conversion of NAD to an Advanced Intermediate. *J. Am. Chem. Soc.* **2007**, *129* (10), 2914–2922. https://doi.org/10.1021/ja067606t.

(11) Goyer, A. Thiamin Biofortification of Crops. *Curr. Opin. Biotechnol.* **2017**, *44*, 1–7. https://doi.org/https://doi.org/10.1016/j.copbio.2016.09.005.

(12) Fitzpatrick, T. B.; Chapman, L. M. The Importance of Thiamine (Vitamin B1) in Plant Health: From Crop Yield to Biofortification. *J. Biol. Chem.* **2020**, *295* (34), 12002–12013. https://doi.org/https://doi.org/10.1074/jbc.REV120.010918.

(13) Atif, M.; Perveen, S.; Parveen, A.; Mahmood, S.; Saeed, M.; Zafar, S. Thiamine and Indole-3-Acetic Acid Induced Modulations in Physiological and Biochemical Characteristics of Maize (Zea Mays L.) under Arsenic Stress. *Sustain.* **2022**, *14* (20). https://doi.org/10.3390/su142013288.

(14) Li, W.; Mi, X.; Jin, X.; Zhang, D.; Zhu, G.; Shang, X.; Zhang, D.; Guo, W. Thiamine Functions as a Key Activator for Modulating Plant Health and Broad-Spectrum Tolerance in Cotton. *Plant J.* **2022**, *111* (2), 374–390. https://doi.org/https://doi.org/10.1111/tpj.15793.

(15) Yi, H.; Ravilious, G. E.; Galant, A.; Krishnan, H. B.; Jez, J. M. From Sulfur to Homoglutathione: Thiol Metabolism in Soybean. *Amino Acids* **2010**, *39* (4), 963–978. https://doi.org/10.1007/s00726-010-0572-9.

(16) HAWKESFORD, M. J.; DE KOK, L. J. Managing Sulphur Metabolism in Plants. *Plant. Cell Environ.* **2006**, *29* (3), 382–395. https://doi.org/https://doi.org/10.1111/j.1365-3040.2005.01470.x.

(17) Takahashi, H.; Kopriva, S.; Giordano, M.; Saito, K.; Hell, R. Sulfur Assimilation in Photosynthetic Organisms: Molecular Functions and Regulations of Transporters and Assimilatory Enzymes. *Annu. Rev. Plant Biol.* **2011**, *62*, 157–184. https://doi.org/10.1146/annurev-arplant-042110-103921.

(18) Li, Q.; Gao, Y.; Yang, A. Sulfur Homeostasis in Plants. *Int. J. Mol. Sci.* **2020**, *21* (23), 1–16. https://doi.org/10.3390/ijms21238926.

(19) Amir, R. Current Understanding of the Factors Regulating Methionine Content in Vegetative Tissues of Higher Plants. *Amino Acids* **2010**, *39* (4), 917–931. https://doi.org/10.1007/s00726-010-0482-x.

(20) Rey, P.; Tarrago, L. Physiological Roles of Plant Methionine Sulfoxide Reductases in Redox Homeostasis and Signaling. *Antioxidants* **2018**, *7* (9). https://doi.org/10.3390/antiox7090114.

(21) Couturier, J.; Chibani, K.; Jacquot, J.-P.; Rouhier, N. Cysteine-Based Redox Regulation and Signaling in Plants. *Front. Plant Sci.* **2013**, *4*, 105. https://doi.org/10.3389/fpls.2013.00105.

(22) Yu, L.; Zhou, C.; Fan, J.; Shanklin, J.; Xu, C. Mechanisms and Functions of Membrane Lipid Remodeling in Plants. *Plant J.* **2021**, *107* (1), 37–53. https://doi.org/https://doi.org/10.1111/tpj.15273.

(23) van der Rest, B.; Boisson, A.-M.; Gout, E.; Bligny, R.; Douce, R. Glycerophosphocholine Metabolism in Higher Plant Cells. Evidence of a New Glyceryl-Phosphodiester Phosphodiesterase. *Plant Physiol.* **2002**, *130* (1), 244–255. https://doi.org/10.1104/pp.003392.

(24) Guo, Q.; Liu, L.; Rupasinghe, T. W. T.; Roessner, U.; Barkla, B. J. Salt Stress Alters Membrane Lipid Content and Lipid Biosynthesis Pathways in the Plasma Membrane and Tonoplast. *Plant Physiol.* **2022**, *189* (2), 805–826. https://doi.org/10.1093/plphys/kiac123.

(25) Nakamura, Y. Plant Phospholipid Diversity: Emerging Functions in Metabolism and Protein-Lipid Interactions. *Trends Plant Sci.* **2017**, *22* (12), 1027–1040. https://doi.org/10.1016/j.tplants.2017.09.002.

(26) Welti, R.; Li, W.; Li, M.; Sang, Y.; Biesiada, H.; Zhou, H.-E.; Rajashekar, C. B.; Williams, T. D.; Wang, X. Profiling Membrane Lipids in Plant Stress Responses. Role of Phospholipase D Alpha in Freezing-Induced Lipid Changes in Arabidopsis. *J. Biol. Chem.* **2002**, *277* (35), 31994–32002. https://doi.org/10.1074/jbc.M205375200.

(27) Hou, Q.; Ufer, G.; Bartels, D. Lipid Signalling in Plant Responses to Abiotic Stress. *Plant. Cell Environ.* **2016**, *39* (5), 1029–1048. https://doi.org/10.1111/pce.12666.
